# Evaluation of objective tools and artificial intelligence in robotic surgery technical skills assessment: a systematic review

**DOI:** 10.1093/bjs/znad331

**Published:** 2023-11-10

**Authors:** Matthew W E Boal, Dimitrios Anastasiou, Freweini Tesfai, Walaa Ghamrawi, Evangelos Mazomenos, Nathan Curtis, Justin W Collins, Ashwin Sridhar, John Kelly, Danail Stoyanov, Nader K Francis

**Affiliations:** The Griffin Institute, Northwick Park & St Marks’ Hospital, London, UK; Wellcome/ESPRC Centre for Interventional Surgical Sciences (WEISS), University College London (UCL), London, UK; Division of Surgery and Interventional Science, Research Department of Targeted Intervention, UCL, London, UK; Wellcome/ESPRC Centre for Interventional Surgical Sciences (WEISS), University College London (UCL), London, UK; Medical Physics and Biomedical Engineering, UCL, London, UK; The Griffin Institute, Northwick Park & St Marks’ Hospital, London, UK; Wellcome/ESPRC Centre for Interventional Surgical Sciences (WEISS), University College London (UCL), London, UK; The Griffin Institute, Northwick Park & St Marks’ Hospital, London, UK; Wellcome/ESPRC Centre for Interventional Surgical Sciences (WEISS), University College London (UCL), London, UK; Medical Physics and Biomedical Engineering, UCL, London, UK; Department of General Surgey, Dorset County Hospital NHS Foundation Trust, Dorchester, UK; Division of Surgery and Interventional Science, Research Department of Targeted Intervention, UCL, London, UK; University College London Hospitals NHS Foundation Trust, London, UK; Division of Surgery and Interventional Science, Research Department of Targeted Intervention, UCL, London, UK; University College London Hospitals NHS Foundation Trust, London, UK; Division of Surgery and Interventional Science, Research Department of Targeted Intervention, UCL, London, UK; University College London Hospitals NHS Foundation Trust, London, UK; Wellcome/ESPRC Centre for Interventional Surgical Sciences (WEISS), University College London (UCL), London, UK; Computer Science, UCL, London, UK; The Griffin Institute, Northwick Park & St Marks’ Hospital, London, UK; Division of Surgery and Interventional Science, Research Department of Targeted Intervention, UCL, London, UK; Yeovil District Hospital, Somerset Foundation NHS Trust, Yeovil, Somerset, UK

## Abstract

**Background:**

There is a need to standardize training in robotic surgery, including objective assessment for accreditation. This systematic review aimed to identify objective tools for technical skills assessment, providing evaluation statuses to guide research and inform implementation into training curricula.

**Methods:**

A systematic literature search was conducted in accordance with the PRISMA guidelines. Ovid Embase/Medline, PubMed and Web of Science were searched. Inclusion criterion: robotic surgery technical skills tools. Exclusion criteria: non-technical, laparoscopy or open skills only. Manual tools and automated performance metrics (APMs) were analysed using Messick's concept of validity and the Oxford Centre of Evidence-Based Medicine (OCEBM) Levels of Evidence and Recommendation (LoR). A bespoke tool analysed artificial intelligence (AI) studies. The Modified Downs–Black checklist was used to assess risk of bias.

**Results:**

Two hundred and forty-seven studies were analysed, identifying: 8 global rating scales, 26 procedure-/task-specific tools, 3 main error-based methods, 10 simulators, 28 studies analysing APMs and 53 AI studies. Global Evaluative Assessment of Robotic Skills and the da Vinci Skills Simulator were the most evaluated tools at LoR 1 (OCEBM). Three procedure-specific tools, 3 error-based methods and 1 non-simulator APMs reached LoR 2. AI models estimated outcomes (skill or clinical), demonstrating superior accuracy rates in the laboratory with 60 per cent of methods reporting accuracies over 90 per cent, compared to real surgery ranging from 67 to 100 per cent.

**Conclusions:**

Manual and automated assessment tools for robotic surgery are not well validated and require further evaluation before use in accreditation processes.

PROSPERO: registration ID CRD42022304901

## Teaser

This systematic review provides a comprehensive evaluation of current objective, manual, automated and artificial intelligence (AI) methods used in robotic technical skills assessment. Many lack full evaluation and AI is in its conceptual stages.

## Introduction

Robotic surgery is increasingly being adopted due to improved vision, dexterity and surgical ergonomics. In selected procedures there is supportive evidence demonstrating non-inferiority and lower morbidity compared to laparoscopy^1–5^. Minimally invasive surgery (MIS) is complex, highly variable and requires technical skill with unfavourable error profiles compared to industrial data^6^. Meanwhile, the addition of new technology into the operating room, with novel technical and non-technical considerations, increases the potential for human error, and therefore patient risk^7^. Of surgical patients, 10–15 per cent in the UK experience adverse events, of which 50 per cent are preventable^8^. Adverse events relating to robotic procedures (10 624) were reported in the USA between 2000 and 2013^9^ while a global independent review on health technology hazards identified a lack of robotic surgical training as one of the top 10 risks to patients^10^. This deficit is being addressed through development and standardization of basic and specialty curricula^11–23^.

Robotic surgical procedures require high levels of experience. Evaluation of performance in surgery is shifting from time- and operative numbers-based assessment towards proficiency-based training and accreditation^24^. To assist this, objective tools are frequently employed but must be fully evaluated if they are to be used as summative, high-stakes assessment instruments. Traditionally, proficiency in surgery was extrapolated from clinical outcomes such as histopathology, morbidity and mortality, yet these are subject to multifactorial influences. Intraoperative performance analysis has proved to be a fruitful area for assessment of performance and intervention delivery^6,25–27^. This facilitates direct formative feedback to guide reduction in proficiency curves, as well as summative assessment^26^. Objective assessments within MIS have demonstrated reliability and clinically relevant validities^6,25–27^, leading to the development of tools to aid this^28^. However, previous studies have highlighted the variability in reporting on the validity and reliability of manual tools^28,29^, which risks undermining truly objective skills assessment. A full appraisal of the literature on objective assessment tools, therefore, is imperative to inform learning and accreditation processes in robotic surgery.

Prior systematic reviews have focused on one aspect of technical skills assessment^30,31^ or combinations of surgical approaches^29^. Other reports provide an overview but lack scope and granularity of type of validity and reliability of assessment or fail to grade the evidence^32^. Finally, given the rapid uptake of robotic techniques and development of artificial intelligence (AI) methods, many reports are now outdated^33–37^, requiring up-to-date evaluation^38–40^.

The aim of this systematic review is to provide an up-to-date and comprehensive evaluation of objective, technical skill assessment tools in robotic surgery.

## Methods

This systematic review followed an *a priori* protocol (PROSPERO registration ID CRD42022304901). The Covidence® platform was used to screen studies, exclude duplications and extract data.

### Search strategy

A systematic search of the literature was conducted in line with the PRISMA guidelines^41^. Ovid Embase/Medline, PubMed and Web of Science databases were searched from conception to 22 February 2022. *Table S1* outlines the full search strategy. Searches were performed independently by two authors using medical subject headings (MeSH) terms for ‘Objective’, ‘Assessment’, ‘Tool’, ‘Error’, ‘Skill’, ‘Robot’ and ‘Surgery’, which were combined with Boolean operators ‘AND’ and ‘OR’. Studies from knowledge of the field and references from relevant articles, including one literature^32^ and four systematic reviews^28,29,31,42^, were additionally screened. Conference proceedings and journal supplement abstracts were considered relevant if meaningful data were available.

### Selection of eligible studies

Four reviewers independently screened, reviewed and extracted data, with the primary investigator reviewing all articles. Disagreements were resolved through discussion with the corresponding author.

Included studies followed the PICO question:

- Population—participants being assessed on robotic technical skill.
- Intervention—an objective technical skill assessment tool or method is developed and/or implemented.
- Comparison—to other tools or measurement of assessment.
- Outcome—validity, reliability, accuracy, impact on the participant.

Exclusion criteria were solely laparoscopic and/or open assessment skills, or failures to retrieve the article or an English translation.

### Data extraction

#### All studies

**Fig. 1:**
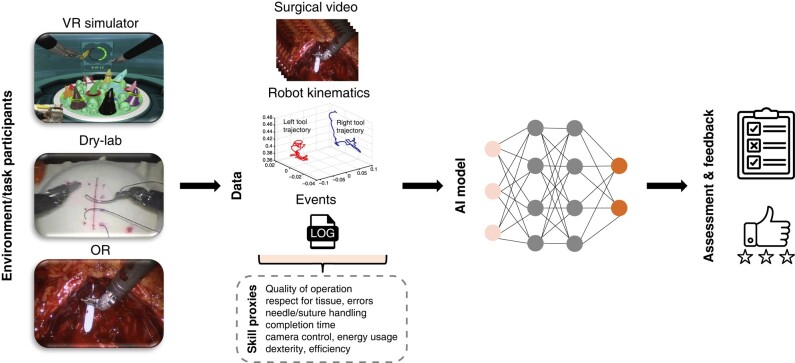
Artificial intelligence framework for surgical skill assessment

Study details including year, country, participant number, participant expertise level and evaluator type were extracted. Identified studies were grouped based on study and tool types into manual, automated performance metrics (APMs) and evaluation of statistical models or AI algorithms. These domains of technical skill assessment were devised using approaches employed by previous reviews and that reflect different assessment methods. The manual domain is human assessment with subgroups that are global rating scales, procedure-specific and error-based tools. APMs are metrics produced by computer software typically in virtual reality (VR) simulators. Finally, AI algorithms are mathematical models implemented to process input data and estimate skill or clinically related outputs, for example, using kinematic data to predict postoperative urinary incontinence^36^ or vision data to predict skill level (*Fig. 1*).

#### Manual and APM studies

Due to the heterogeneity in methods of technical skill assessment, different approaches were applied to facilitate evaluation. Manual tools and automated performance metrics (non-AI articles) were evaluated using Messick's validity concept^43^ and the Modified Educational Oxford Centre for Evidence-Based Medicine (OCEBM) Levels of Evidence (LoE) and Levels of Recommendation (LoR)^44^. Messick's concept views validity as a continuous process and a combination of the classical views of face, content, construct and predictive validity, internal consistency, intra- and inter-rater reliability. Instead of viewing these as separate, five aspects that need to be considered for an assessment tool to be valid were assessed (*Table 1*). Strength of correlational analyses and significance was also extracted using standardized definitions.

**Table 1:** Messick's validity framework adapted from^143,144^

Source of validity	Evidence	Examples
Test content, that is face and content validity	The test's content and the construct it is intended to measure	Delphi methodology/expert consensus development Questionnaires for realism and usefulness
Response process	Analysis of raters, that is how well they respond to the test and steps taken to improve the validity	Rater training/orientation/familiarization Randomization Powered study APMs eliminate rater bias
Internal structure, that is reliability	Degree to which domains and aspects of the tool fit the underlying construct	Intra- and inter-rater reliability, internal consistency APMs eliminated rater subjectivity
Relationship to other variables	Evaluating scores' associations/correlations, whether they are positive or negative, strong or weak, and with other variables including discriminative ability	Concurrent, construct and predictive validity Generalizability of the evidence Learning curves
Consequence	Impact of the assessment	Pass/fail/benchmarking of scores Impact or consequences on participants future/learning

#### Artificial intelligence studies

AI specialists contributed to screening, data extraction and evaluation and a bespoke data extraction template was employed to standardize data capture.

#### Methodological quality assessment

Methodological quality assessment was evaluated using a modifiable Downs–Black checklist (*Table S2*)^45^. Due to study heterogeneity some aspects were not applicable; therefore, taking a pragmatic approach, we modified the score in these circumstances, with a maximum score of 10 available. For AI studies, it was not feasible to apply a relevant methodological quality tool such as the Downs–Black checklist or Medical Education Research Study Quality Instrument (MERSQI)^46^, as most study designs are conceptual.

*Tables 2–4* summarize the main tools in each domain of technical skill assessment and *Supplementary Tables (Tables S3 to S8)* describe each study's analysis. Summaries of the remaining tools can be viewed in *Table S9*.

**Table 2:** Summary of manual assessment tools

Global rating scale tools									
Tool	Study type	Setting	Test content	Response process	Internal structure	Relationship to other variables	Consequences	LoE	LoR
GEARS	46 Observational 12 RCTs	36 Lab 17 OR 5 Lab & OR	Experts developed based on GOALS^145^	All studies (except one^53^) demonstrated response process	*Inter-rater reliability* Strong/Very strong/Excellent/High^47–51,71,82,83,145–156^ Acceptable/Good/Moderate^15,48,51,157,158^ Low/Poor^50,159^ *Intra-rater reliability* Excellent^148^ *Internal consistency* Excellent^105,145,160^ Low^49^	*Concurrent validity* Other GRS tools^49^ Task-/procedure-specific tools^77,80,81,146,161^ Error tools^162^ Virtual reality^146,163–166^ APMs^52,84,105,154,157^ Non-technical skills^167^ *Construct validity* ^47,52,77,82,84,99,105,145,147,148,150,151,153,154,157,161,163,167–174^ *Predictive validity* ^53,162,175^	80–100% = good to excellent (arbitrary definition^73^) Benchmarked using expert scores^167,169^	Level 1b^15,83,169^ Level 2a^82,149,153,159,160,165,166,168,176,177^ Level 2b^47–52,77,80,81,84,99,105,113,145–148,150,151,154–156,161,163,164,167,170,171,174,178^ Level 3 ^53,71,73,152,158,162,172,179–182,282^ Level 4^58,64,175^	Level 1 recommendation
OSATS	20 Observational 3 RCT	19 Lab 3 OR 1 Lab & OR		All but two studies^183,184^ demonstrated response process	*Inter-rater reliability* High/Excellent^90,185,186^ Good/Moderate^86,187–189^ *Intra-rater reliability* Strong^86^ *Internal consistency* Excellent^105NB OSATS/GEARS combo^	*Concurrent validity* Task-/procedure-specific tools^90^ Error tools^190^ Virtual reality^90,191^ APMs^105^ Cognitive load^192^ *Construct validity* ^54,62,105,185,187,188,193,194^ *Predictive validity* ^86^	Hypothesized mean OSATS category scores 3.5 novice and 4.5 expert was in concordance with results of mean^188^ Expert benchmarking^62,184,185^	Level 2a^63,195,196^ Level 2b^54,62,90,105,183,185–188,189,192–194^ Level 3^86,88,184,190,191,197,198^	Level 2 recommendation (NB if combined with OSATS task-specific, it would still be LoR 2)
GOALS	4 Observational 3 RCTs	5 Lab 1 OR 1 Lab & OR	Expert group added 2 domains to GOALS creating GOALS + ^199^	All studies demonstrated response process	*Internal consistency* High^160^	*Concurrent validity* Other GRS tools^160^ Virtual reality^160,200^ *Construct validity* ^54,199,201^	Pass mark defined by contrasting groups method^54^ by experts^92^	Level 1b^200^ Level 2a^160^ Level 2b^54,92,199,201^ Level 4^58^	Level 2 recommendation
R-OSATS	4 Observational	4 Lab	Developed from GOALS and OSATS^202^	All studies demonstrated response process	*Inter-rater reliability* Strong/Very high/Excellent^55,203,204^ Acceptable/Moderate/Good^202,203^ *Intra-rater reliability* Very strong^202^ Moderate/Good^203^	*Concurrent validity with VR* ^204^ *Construct validity* ^202,204^	Modified Angoff method set threshold competency scores per drill^55^	Level 2b^55,202,204^ Level 3^203^	Level 2 recommendation
**Procedure- and Task-Specific Assessment Tools**									
*OSATS Task-specific* Tools are for separate procedures/tasks	5 Observational 2 RCTs	7 Lab	No studies	All demonstrated response process	*Inter-rater reliability* High^90^	*Concurrent validity* Non-simulator APMs^89^ Simulator APMs^90^ *Construct validity* ^62,98^	Pass mark based on experts^62^	Level 2a^63^ Level 2b^62,90,98^ Level 3^88,89,91^	Level 2 recommendation (NB if combined with OSATS GRS, would still be Level 2)
RACE	5 Observational 1 RCT	3 Lab 2 OR 1 Delphi and OR	Delphi consensus^81^	All demonstrated response process	*Inter-rater reliability* Strong/Excellent^50,83^ Good/Moderate^50^ *Intra-rater reliability* Good^81^	*Concurrent validity* GEARS^81^ UVA leak on model^84^ EASE suturing tool^205^ *Construct validity* ^81,84^		Level 1b^83^ Level 2b^50,81,84,205^ Level 3^78^	Level 2 recommendation
*Task-Perform-ance Metrics Tools* Tools are for separate procedures/tasks	1 Observational 2 Delphi and video rating OR videos 2 Delphi and Lab	1 Delphi 1 OR 1 Delphi and OR 2 Delphi and OR	All tools developed through Delphi consensus	All tools demonstrated response process	*Inter-rater reliability* Percentage agreement 0.85–0.96^76,77,93,97^	*Construct validity* ^76,77,93,97^	Anastomotic leak test^93^	Level 2b^76,77,93,97^ Level 4^75^	Level 2 recommendation
**Error Assessment Tools**									
FLS Scoring System	8 Observational 1 RCT 2	8 Lab 1 OR and Lab	None	2 demonstrated response process^169,206^	*Inter-rater reliability* Excellent^207^ *Intra-rater reliability* Excellent^207^ *Internal consistency* Good^207^	*Construct validity* ^169,208–210^ *Concurrent validity with GEARS* ^146^	Expert defined proficiency/pass fail marks^169,207–209^	Level 2a^169^ Level 2b^146,206,208,209^ Level 3^95,207,209,211^	Level 2 recommendation
*Task-Perform-ance Metrics Tools* Tools are for separate procedures/tasks	1 Observational 2 Delphi and video rating OR videos 2 Delphi and Lab	1 Delphi 1 OR 1 Delphi and OR 2 Delphi and OR	All tools developed through Delphi consensus	All tools demonstrated response process	*Inter-rater reliability* Percentage agreement 0.85–0.96^76,77,93,97^	*Construct validity* ^76,77,93,97^	Anastomotic leak test^93^	Level 2b^76,77,93,97^ Level 4^75^	Level 2 recommendation
Generic Error Rating Tool	2 Observational	2 OR	None	Both demonstrated response process		*Concurrent validity* GEARS^162^ Clinical adverse events (presumed intra-op, unclear)^162^ Cognitive task load^192^		Level 2b^192^ Level 3^162^	Level 3 recommendation

**Table 3:** Summary of automated performance metrics

Simulator automated performance metrics									
Tool	Study type	Setting	Test content	Response process	Internal structure	Relationship to other variables	Consequences	LoE	LoR
da Vinci (Surgical) Skills Simulator	28 Observational 11 RCTs	30 Lab 9 OR and Lab	14 studies showing face and content validity^83,164,168,201,204,212–220^ Note three studies stated dVSS more realistic/helpful than dV-T^83,219,220^	Automated metrics inherently no rater bias 11 RCTs (3 not powered)^159,160,165^ 2 Powered non-randomized studies^201,212^ Orientation/standardization of training^201,212,215,216,218,221,222^	Automated metrics inherently consistent 1 study measured consistency with consecutive attempts with excellent reliability^217^	*Concurrent validity* GRS tools^160,163–165,200,204^ Other VR^219^ Error tools^163^ Other training method^223^ *Construct validity* ^163,181,201,204,212–215,221,222,224–226^ *Predictive of console performance in the lab* ^83,194,200,201^ *Predictive of console performance in the OR* ^146,166,201,227^	Pass mark defined ^92,160,166,168,169,194,201,204,210,216,217,225,228–230^	Level 1b^83,200^ Level 2a^159,160,165,166,168,169,177^ Level 2b^146,163,164,201,204,212–217,221–226,228,229^ Level 3^73,92,181,191,194,210^ Level 4^220^	Level 1 recommendation
dV-Trainer	13 Observational 4 RCTs	17 Lab	9 Face/content^83,90,193,218–220,231–233^ Note lack of realism for needle driving^231^	Automated metrics inherently no rater bias 4 RCTs (2 not powered)^165,234^ Orientation/standardization of training^218,232,235,236^	Automated metrics inherently consistent	*Concurrent validity* GRS^90,165,218^ Other VR^219^ *Construct validity* ^218,219,231–233,236,237^ *Predictive of console performance in the lab* ^193,234,238^	Novice proficiency criteria developed^149^ Defined by expert performance^235^ VR index competency score (not benchmarked)^234^	Level 1b^83^ Level 2a^149,165,234,235^ Level 2b^90,193,218,219,231–233,236,237^ Level 3^238,239^ Level 4^220^	Level 2 recommendation
RobotiX Mentor	9 Observational 1 RCT	9 Lab 1 Delphi	All studies showed face/content except two^173,240^ No difference between RXM and dVSS/dV-T^220^ Note limitation of realism of suture^241^	Automated metrics inherently no rater bias Orientation/standardization of training^102,103,173,241–243^ Randomized groups^173^	Automated metrics inherently consistent *Test–retest reliability* Excellent^103^ *Internal consistency of metrics* Fair^243^ Unacceptable to good^143^ Unacceptable to poor^103^	*Concurrent with FRS metrics dry lab* ^244^ *Construct validity* ^103,143,240–244^ *Predictive of console performance in the lab* ^173^	Pass mark defined ^241^ based on competent surgeons^240,244^, with contrasting groups method ^103,43,243^	Level 2a^173^ Level 2b^102,103,143,241–244^ Level 2c^240^ Level 4^220^	Level of recommendation 2
Promis^TM^ hybrid surgical simulator	3 Observational	3 Lab	Face and content^245^	Standardized orientation^245,246^	*Internal consistency* Good^247^	*Construct validity* ^245–247^		Level 2b^245–247^	Level 2 recommendation
Robotic Surgery Simulator (RoSS)	3 Observational 1 RCT	1 Lab 1 Lab and Delphi	Face^248^ and content ^12,249,250^	Automated metrics inherently no rater bias Randomized, powered^12^	Automated metrics inherently reliable	*Construct validity* ^250^ *Predictive of console performance in the lab* ^12^		Level 2a^12^ Level 2b^250^ Level 4^248,249^	Level 2 recommendation
3D hydrogel models—clinically relevant objective/performance metrics (CROMS/CRPMS)	2 Observational	2 Lab	Face and content both studies	Objective metrics Pilot testing^52^		*Concurrent validity with GEARS* ^52,84^ *Construct validity* ^52,84^		Level 2b^52,84^	Level 2 recommendation
da Vinci SimNow^251^	1 RCT	Lab		Automated metrics inherently without rater bias	Automated metrics inherently reliable	*Concurrent validity with da Vinci system event data recorder* *Construct validity*		Level 2a	Level of Recommendation 3
Versius Trainer^172^	1 Observational	Lab		Automated metrics inherently without rater bias	Automated metrics inherently reliable	*Learning curve demonstrated*		Level 3	Level of Recommendation 4
**Non-Simulator Automated Performance Metrics**									
*da Vinci Kinematic and System Event Recorders* NB different terms used that is da Vinci API, dVLogger or da Vinci Systems Events data recorder	15 Observational 1 RCT	10 Lab 6 OR		Automated metrics inherently without rater bias 1 study randomized^251^	Automated metrics inherently reliable	*Concurrent validity* GRS^89,154,157^ VR^251^ Cognitive load^251^ Task evoked pupillary response^252^ R.E.N.A.L. nephrotomy score and intraop data for example EBL^35^ *Construct validity* ^35,37,157,251–256^ *Predictive validity* ^35–37,256^		Level 2a^251^ Level 2b^35,37,154,157,198,252–256^ Level 3^36,89,189,257,258^	Level of Recommendation 2
Electromag-netic motion tracker sensor (TrakStar; Ascension Technologies, USA)	5 Observational	All Lab		Automated metrics inherently without rater bias Standardized orientation^259^ Experts reviewed metrics^260^	Automated metrics inherently reliable	*Construct validity* ^206,259,261,262^		Level 2b^206,259,261,262^ Level 3^260^	Level 2 recommendation
SurgTrak™ Motion tracking^235^	1 RCT	Lab		Automated metrics inherently without rater bias Randomized, powered		*Construct validity*		Level 2a	Level 2 recommendation

**Table 4:** Summary of artificial intelligence studies

Study setting	Participant no.	Tasks/procedure	Data set	Data set size (trials)	Model input	Model output that is estimates/predictions | performance
Simulators (VR)	1–9^124^ 10–19^122,125,126,263^ ≥ 20^107^	Ring and rail^107,122,124,125,263^ Suture sponge^107,122,125^ Camera targeting, peg board, dots and needles, tubes^124^ Endowrist manipulation, needle control and needle driving skills^126^	All private	<50^124,263^ 50–99^122,125,126^	Kinematics^107,122,125,126,263^ Skill-related labels^125,126^ EMG signals^125^ Eye-tracking and EEG signals^124^	Skill level^125,263^ | accuracy 65–100% GEARS^107,126^ | accuracy 69–89% Skill-related labels^122^ | accuracy 48–99%
Dry lab	1–9^118,120,121,127,129–132,134,135,136,198,264–275^ 10–19^105,276–278^ ≥ 20^152^	Suturing^120,121,131,132,264–266,276,277^ Needle passing^118–121,127–132,134,136,137,265,267–270,272,274,275,279^ Knot tying^118,120,121,127,129–132,134,136,137,198,265–270,272,274,277,278^ Transection and dissection^264,276^ Peg transfer^105,152^ Ring transfer^280^	JIGSAWS^110,111,118–121,127–132,134,135,137,265–269,272–275,277,279^	<50^271,274^ 50–99^136^ 100–149^110,111,118–121,127–132,134,135,137,152,198,265–270,272–275,277–279^ ≥ 150^105^	Kinematics^105,118–120,129,134–137,152,198,264–266,269,271,274–278,281^ Force data^105,264^ System event data^264,271,276,281^ Videos^121,127,128,130–132,267,268,270,272,273,279^	Skill level^118,127,134–137,198,265,266,268,269,271,274,276,277,280,281^ | accuracy 46–100% Modified OSATS (JIGSAWS)^111,118–120,127,128,131,132,267,270,272^ | SCC 0.03–0.93 GEARS^152^ | accuracy 52–75%
Operating room	1–9^106,110,111,115^ 10–19^33,112^ ≥ 20^36,114,138^	RARP^33,36,112,114,115,138^ Urethrovesical anastomosis^33,112^ Needle handling/driving^138^ Lymph node dissection^106,110^ Laparoscopic cholecystectomy^108,111^	HeiChole^111^	<50^106,110–112,117^ 50–99^33,114,116^ 100–149^115^ ≥ 150^36,108,138^	Videos^106,108,110–112,116,117,138^ Kinematics^33,36,115^ System event data^33,36,115^ Clinical parameters^114,115^	Skill level^33,108,112,117,138^ | accuracy 67–100% PLACE score^106^ | accuracy 83.3% Modified GOALS^110,111^ | SCC 0.46–0.57 3-Month/6-month urinary continence after RARP^36^ | AUC 0.67–0.74) 1-Year erectile function recovery^114^ | AUC 0.68–0.77

## Results

**Fig. 2:**
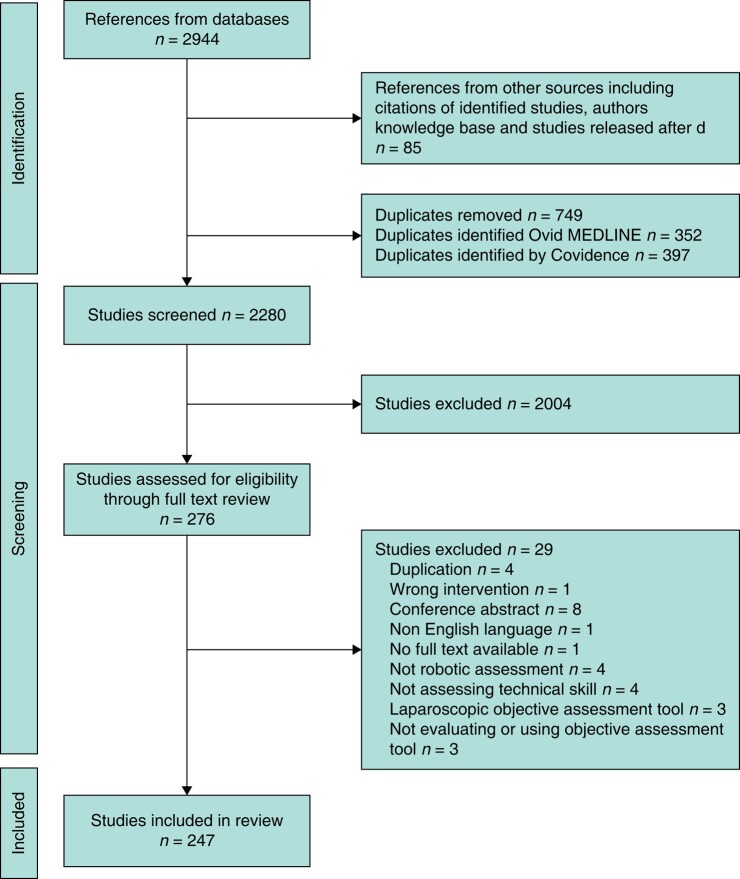
PRISMA flowchart

Two thousand, nine hundred and forty-four studies were identified from searches with 85 identified from additional sources. Seven hundred and forty-nine duplicates were removed. Of 2280 studies that were screened and reviewed, 2033 were excluded with 247 studies undergoing data extraction (*Fig. 2*). Two hundred and twenty-seven studies were classified as observational, including Delphi meetings, experimental, cohort and randomized studies not defined as randomized control trials (RCTs), while a total of 20 RCTs were identified. Of the manual studies, 93 used global rating scales (GRS), 45 procedure- or task-specific tools, 43 error-based, 77 simulator automated performance metrics, 28 non-simulator automated performance metrics and 53 AI studies.

### Global rating scale tools

Eight different GRS tools were identified (*Table 2* and *Table S3*). Global Evaluative Assessment of Robotic Skills (GEARS) was the most utilized assessment method, assessed in 58 studies, including 12 RCTs giving a Level 1 recommendation based on 21 studies reporting excellent reliability and 3 low/poor. Interestingly, crowd-sourced GEARS ratings demonstrated excellent inter-rater reliability^47^, good to strong/excellent inter-observer group reliability compared to experts^48–51^, as well as construct^48,52^ and predictive validity^53^. GEARS (all raters) demonstrated supportive evidence of ‘relationship to other variables’ including concurrent (17 studies), construct (25 studies) and predictive validity (3 studies).

Objective Structured Assessment of Technical Skills (OSATS), Global Operative Assessment of Laparoscopic Skills (GOALS) and Robotic-OSATS (R-OSATS) were used in a total of 34 studies and all received a Level 2 recommendation, despite only one of them being robotic-specific. GEARS has no robust data validating a benchmark for overall and domain scores, whereas GOALS and R-OSATS used the contrasting groups method^54^ and the modified Angoff method^55^, setting competency at 80 per cent and 70 per cent, respectively. All other tools identified have not been thoroughly evaluated with LoR 3 or 4 (*Table S9*).

### Procedure- and task-specific tools

Twenty-six different types of procedure- and task-specific tool were identified in 45 studies (*Table 2*, *Tables S4, S9*). Of these, 22 (48.9 per cent) studies containing 17 (37.8 per cent) different tools used full procedural data^56–77^. With regards to specialty, 21 (46.7 per cent) studies^50,56,57,64,66–74,76,78–84^ assessed 15 urology tools, 9 (20 per cent) studies^58–61,63,75,77,85,86^ assessed 8 general surgery tools, 3 (6.7 per cent) gynaecology tools^58,65,87^, 1 (2.2 per cent) ear, nose and throat^62^, 1 (2.2 per cent) microsurgery^88^, 8 (17.8 per cent) suturing^89–95^, 2 (4.4 per cent) dissection^92,96^, 1 (2.2 per cent) vessel dissection and ligation^97^ and 1 (2.2 per cent) other dry model tasks^98^.

Robotic Anastomosis Competency Evaluation (RACE), Task-Performance Metrics tools and OSATS are the most evaluated tools with a Level 2 recommendation, demonstrating concurrent and construct validity, missing evaluation for predictive and in the consequence domain. One tool, A-OSATS^85^, has been evaluated over all five of Messick's domains, but only in one study and lacking predictive validity. In summary, there are no full procedural tools that have been fully evaluated for robotic surgery.

### Error tools

Three main tools underwent multiple study evaluations (*Table 2*): the Fundamental Laparoscopic Skills (FLS) scoring system, Generic Error Rating Tool (GERT) and Task-Performance Metrics. The most common error method was the cumulative number of errors, reported in 20 of 42 studies (46.5 per cent; *Table S5*). In 13 studies (69.7 per cent) a composite score was created, while a further study defined arbitrary task-specific time penalties. There is substantial variability in the definition and measurement of errors, often missing robust evaluation on validity and multiple tools were only present in single studies. The FLS scoring system gives a composite score and was used in 9 (20.9 per cent) studies. Task-Performance Metrics tools define errors and were used in 5 (11.6 per cent). The GERT tool assesses a surgical task group, error mode, number, description and mechanism of event and was analysed in 2 (4.6 per cent) studies. All three methods reached Level 2 recommendation. FLS and Task-Performance Metrics tools both had evidence of internal structure and relationship to other variables, with excellent reliability, concurrent and construct validity. There were no reports on predictive validity or benchmarking of these tools.

### Simulator automated performance metrics

Ten different simulators were identified (*Table S6*, *Table 3*). Automated performance metrics in simulation environments have been thoroughly evaluated with 39 (50.6 per cent) studies on da Vinci Skills Simulator (dVSS), 17 on (22.1 per cent) dV-Trainer (dV-T) and 9 (11.7 per cent) on RobotiX Mentor. Sixteen (76.2 per cent) of the 20 RCTs in this review involved simulators. These three simulators have been validated in all five Messick domains exhibiting concurrent and construct validity. dVSS and dV-T training also predicted better performance on the console in operative and dry model performances. In addition, more comprehensive evaluation in the consequence domain has been carried out for all three when compared to other assessment tools. Current evidence favours dVSS at Level 1 recommendation. dV-T, RobotiX Mentor, Promis hybrid surgical simulator, Robotic Surgery Simulator (RoSS) and 3D hydrogel models with ‘Clinically Relevant Objective or Performance Metrics (CROMS/CRPMS)’ all receive Level 2 recommendation. The Versius trainer from CMR Surgical has currently been evaluated at Level 4 recommendation. Simulators^99–101,283^ unlikely to be in wide usage were identified and excluded from *Table 3*. Notably the vast majority of studies (70; 90.9 per cent) looked at basic skills, with only 6 (7.8 per cent) reviewing procedure-specific VR^52,84,99,102–104^.

### Non-simulator automated performance metrics

Of the 28 included studies (*Table 3* and *Table S7*), 16 used da Vinci Application Programming Interface (API) kinematic and system event data, with 6 (21.4 per cent) from the operating room and all within urology. Kinematic and system event data from the da Vinci systems are the most evaluated APMs on the console achieving Level 2 recommendation. Despite two other APM tools having the same LoR, da Vinci API data evaluation is arguably more useful as concurrent, construct and predictive validity has been demonstrated. The only study^105^ looking at non-kinematic data, instrument vibration and forces, showed construct and concurrent validity, with a LoR 3. No study has yet fully validated non-simulator APM data, primarily missing evaluation in the consequence domain.

### Artificial intelligence

Fifty-three AI studies were identified (*Table 4* and *Table S8*). The range of participating surgeons across the AI studies varied from 1^106^ to 77^107^ (median = 8). Most studies employed the publicly available JHU-ISI Gesture and Skill Assessment Working Set (JIGSAWS)^109^, which features 139 trials from eight surgeons suturing, knot-tying, and needle-passing exercises with kinematic data.

The most common level grouping was between expert and novice surgeons; however, there was significant heterogeneity in how this was defined. In most cases, expertise was defined by a surgeon's caseload with wide variability, for example 50 to over 2500 cases^33,105^. Other studies assigned assessment scores to group surgeons above a predefined threshold as experts and below as novices^108^.

Forty-one (77.4 per cent) studies evaluated their AI models using data obtained from simulators or dry lab simulations, while 12 (22.6 per cent) studies used data collected from real surgical procedures. The most frequently used dry lab data set (24/41; 58.5 per cent) was JIGSAWS.

Studies that used real surgical data included procedures such as lymph node dissection^110^, laparoscopic cholecystectomy^108,111^, robotic assisted radical prostatectomy (RARP) urethrovesical anastomosis^112^, and phases^36,113–115^, gastrectomy^116^ and thyroid surgery^117^. RARP procedures were the most common (7/12), with Chen *et al.*^33^ utilizing the largest data set.

The majority approached skill assessment as a classification task (28/53; 52.8 per cent), with the aim of predicting the participant skill level. Twenty (37.7 per cent) studies estimated an assessment score (numerical regression) that corresponds to an assessment tool. Notably, only three^118–120^ attempted to estimate the individual domains of the tool, with the remainder predicting the total score.

A few studies adopted a different approach to assess skill; ranking performance^121^, estimating the operating field clearness^116^, using stylistic behaviour labels^122^ and linking skill levels to clinical outcomes in RARP^36,113,115,123^.

Of the 53 studies, 20 (37.7 per cent) utilized video data, 29 (54.7 per cent) used kinematics, 7 (13.2 per cent) employed system events and 3 (5.6 per cent) used force data. Furthermore, a few others utilized clinical parameters such as BMI and prostate -specific antigen (PSA)^114^, eye-tracking and electroencephalography (EEG) signals, electromyography data (EMG) and galvanic skin response (GSR)^124^, surgical gesture sequences^114^ and stylistic behaviour components^122,125,126^. Among these studies, 33 (62.3 per cent) used a single input modality (for example, video only), while 20 (37.7 per cent) utilized two or more input modalities.

Twenty-six (49.1 per cent) studies used classic machine learning methods, with support vector machine (SVM) being the most common (13/26 (50 per cent)). Most used APMs as input. Twenty-seven (51 per cent) employed deep learning methods, with 19 (35.8 per cent) using convolutional neural networks (CNN). Video-based deep learning methods used a CNN to extract visual features, which are then either fed to a temporal model^110,111,127–131^ or to a simple classifier/regressor^108,112,116,131,132^. Kinematic-based deep learning approaches use either temporal convolutional networks (TCN)^110,133,134^ or recurrent neural network (RNN)^129,135^ or a combination of the two^119,136,137^. Notably, deep learning approaches have gained popularity in surgical skill assessment (*Fig. S1*).

To evaluate their developed methods, most studies utilized the accuracy metric and Spearman's correlation coefficient (SCC). The accuracy rates and SCC for the models tested on real surgical data ranged from 67 per cent to 100 per cent and 0.41 to 0.64, respectively, but were inferior to simulator/dry-lab data; nearly 60 per cent of classification methods reported accuracy above 90 per cent; while only one study^111^ out of 10 reported SCC over 0.90.

## Discussion

This systematic review comprehensively analysed the current development and evaluation status of objective technical skills assessment tools in robotic surgery. Despite the plethora of publications, it is evident that full evaluation according to Messick's concept is sparse. This may explain the notable lack of reports showing their implementation within day-to-day practice or curricula. Many manual tools are lacking in scope and are arguably unsuitable to be used as summative tools at their current validation status. Emerging evidence in AI has reached the first in-human studies, but these are predominantly conceptual and require full validation. The current review suggests that research efforts should be focused on validating and implementing existing instruments rather than seeking any further robotic surgery assessment methods.

GEARS and VR simulators offer clear opportunities for formative and summative assessment within the basic skills curricula. Simulator studies demonstrated VR participants outperforming controls or an improvement in post-VR curriculum assessments in the operative and laboratory setting. GEARS has not been formally benchmarked and given that it is likely the best manual annotation GRS tool to use with AI models warrants further focused evaluation. Meanwhile, given that AI studies often use OSATS or modified GOALS, efforts are necessary to inform the computer science and surgical community to utilize GEARS instead for robotic global technical ratings. Chen *et al*.^28^ highlighted gaps in the assessment domains of generic robotic skills assessments for GEARS, which provides an opportunity for modification and re-evaluation. VR simulators allow safe transference of basic skills and have defined competency benchmarks before progression to console training, broadly speaking a score between 80 and 90 per cent.

Procedure-specific VR and 3D-printed hydrogel models provide high-fidelity simulation allowing an opportunity for standardized, safe progression to clinical training. These platforms avoid possible ethical, religious and moral issues that can prevent the use of cadaver or live animals. Only six studies were identified evaluating procedure-specific VR, confirming the need for further development and evaluation of different operative VR and 3D model tasks. However, additional issues including training access and the financial implications of these platforms remain unstudied.

Procedure-specific tools can potentially act as excellent formative and summative assessments often with higher reliability than GRS. Three tools (OSATS task-specific, RACE and Task-Performance Metrics) had the highest LoR; however, importantly there are no reports demonstrating predictive validity or benchmarking full procedural tools. Task-Performance Metrics were all developed through Delphi consensus as proficiency-based progression (PBP) assessment tools and had high reliability through trained expert raters undergoing reliability ‘checks’. The tools’ structure includes phases and subtasks for each procedure and can be commended for including operation-specific error metrics. Their intended application is within proficiency-based training, which aims to benchmark phases or modules before moving on to the next stage. However, these among other tools are not yet publicly available, precluding research including external validation efforts.

It is evident that there is a paucity of procedure-specific tools ready for implementation into robotic training curricula. They also lack scope, with the majority in urology, and so as a surgical community it is imperative to both develop tools missing for key operations and fully evaluate existing ones.

Error tools identified in this review typically used cumulative number of errors and have not been fully evaluated within clinical settings. A key aspect in a surgeon's learning curve is to understand the ‘what, where, when, how, why’ and corrective mechanisms of an error, which no current study has reported. Granular methods of surgeons’ technical performance and errors are necessary to train AI, combined with global rating scales and procedure-specific tools, to fully understand the complexities of any operation. Tools should combine each aspect with full comprehensive evaluation before implementation into training curricula. As demonstrated in this review, reliability can most likely be improved through expert, trained raters and quality assurance processes.

APMs and AI are emerging and promising tools to guide training and assessment in robotic surgery. APMs can be considered truly objective, yet need further focused evaluation to understand and benchmark important metrics for construct and predictive validity. While AI models performed well when analysing intraoperative surgical skill data, they generally perform better on simulator/dry lab.

A significant proportion of the AI models tested on simulated data achieved accuracy rates above 90 per cent, while some models tested on real surgical data demonstrated perfect classification performance of surgical skill levels. Despite this, AI-based skill assessment is still in its conceptual stage with four broad areas that need to be addressed: data sets, manual annotation, AI model evaluation and integration into clinical practice.

For the field of automated surgical skill assessment to advance, it is critical to assess models on real surgical data. Additionally, it is crucial to gather data from high-fidelity simulations tasks so AI models can be evaluated for benchmarking and comparison of different methods. Efforts must focus on collecting large, publicly available, diverse data sets, including surgeons with differing levels of expertise and different robotic platforms with matched clinical outcome data. Utilizing diverse data sets will ensure AI models are unbiased and can generalize effectively on unseen surgeons and tasks.

Identified AI studies used different ways to evaluate their methods making direct comparisons challenging and reducing external validity. Testing models on the JIGSAWS data set has highlighted the performance gap between cross-validation schemes such as Leave-One-User-Out (LOUO) and more relaxed schemes such as Leave-One-Super-Trial-Out (LOSO). However, before automated skill assessment can be used in clinical practice it must first be ensured the models can generalize to unseen surgeons. To achieve this, evaluation should be performed with cross-validation schemes (for small data sets), or with large external test sets containing trials from unseen surgeons from different hospitals to ensure generalizability^138–140^. LOSO still remains useful in situations where the performance of a specific surgeon is tracked for proficiency curve analyses.

To achieve integration into clinical practice, it is essential that models can provide not only accurate predictions but also clear, understandable justifications for their decisions that clinicians can trust. As the role of AI in healthcare continues to expand there is increasing awareness of the potential pitfalls and the need for guidance to avoid them^141^, including a recent statement from the World Health Organization^142^.

Increasingly detailed and informative feedback beyond simple scores or skill level labels can help to personalize surgical training. Although there have been some efforts to develop explainable AI models and feedback mechanisms^111,127,128,133^, more research is needed to fully address these issues, focusing on developing methods that are more transparent and interpretable, for example written reports and error-detection capabilities to provide more informative context-specific feedback. Indeed, research is needed investigating human factors with educational specialists to elucidate the best way for skill assessment to be presented and when.

To credential surgeons as competent for independent practice, blinded expert video rating is considered an essential part of accreditation^21^. This requires fully evaluated objective summative assessment tools. Often, surgeons undergoing robotic training are already credentialed, adding additional challenges to standardizing pathways and ensuring patient safety. Undoubtedly, there are many routes to competency and now also emergent robotic systems to consider.

This review has highlighted many assessment domains, with their advantages, disadvantages and future research needs (*Table 5*). To achieve implementation of validated and reliable tools into curricula, collaboration between surgical societies is required. Through expert consensus and large, multicentre, international studies, single tools for each procedure should be developed and fully evaluated. Only then, should they be implemented within curricula as formative and summative tools, or in the evaluation of APMs and AI.

**Table 5:** Summary of all assessment domains

Assessment domain	Advantages	Disadvantages	Further research
Global Rating Scales	Quick to fill in. GEARS is capable of strong validity and reliability. Likely good formative tools for generic technical skill.	Subjective, risk of low reliability. Miss granularity of operative steps; therefore, not to be used solely for summative assessment in procedural training. No evidence used in day-to-day formative/summative assessment or incorporated into curricula.	Benchmarking alongside procedure-specific tools. Incorporation into curricula. Expert consensus on tool to use for training AI, and a standardized method for example experts who are trained with reliability tests.
Procedure-specific	Valid and reliable often as it is easy to agree on what steps have been done that is binary. Useful formative and summative assessments.	Potential to miss how well the surgeon performs an operation. Currently only a few tools and predominantly within urology. No fully evaluated or benchmarked tools exist for robotics.	Benchmarking with GRS tools. Development and full evaluation of more tools within different specialties. Incorporation into training curricula.
Error methods	Evidence of validity and reliability. Important part of formative assessment for the surgeon to improve and summative to indicate competency.	Difficult to define certain aspects for example how and why it occurs. Detailed error analysis takes time consuming retrospective video analysis.	Combine with GRS and procedure-specific operative analysis including benchmarking. Further evaluation of existing tools within MIS. AI recognition of near-misses or pre-errors, errors, and critical errors, through manual annotation.
Automated performance metrics	Promising objective tools which have some validity evidence. No concerns re: reliability or bias. Simulators have been thoroughly evaluated and are a good tool for basic skills transfer to the console.	Simulator APMs for procedure-specific simulation are lacking development and evaluation. Non-simulator APMs—current understanding is limited as to their meaning for formative/summative assessment and patient outcome. Data are protected by industry. Requires expertise in computer science collaborating with industry and clinicians; therefore, used only in research currently.	Simulator evaluation on procedure-specific tasks. Evaluation of emerging robotic platforms VR and APMs. Evaluation of APM data in the clinical setting. Creation of more open data sets or perhaps registries should be considered. Availability of APM data sets depends on collaborating with industry to ensure it can be publicly available.
Artificial intelligence	Promising initial results, with the potential to transform formative and summative assessment, particularly if evaluated with APMs.	In its infancy. Ensuring AI is correctly assessing performance requires highly reliable, manually annotated videos, which is time-consuming, particularly given the numbers needed to train then test. Current results are from small data sets. More challenging to evaluate in the clinical setting with more variability, for example camera and patient movements. External validity of methods to surgeons outside of the research data set. Risk of blocking innovation in the future?	Open data sets/registries. Evaluation with manually annotated/rated videos to help train AI, alongside APMs. Development of more complex laboratory data sets to initially evaluate models, then transfer into multispecialty clinical setting.

### Limitations

This comprehensive review standardized data extraction with Messick's concept and modified OCEBM guidelines. Nevertheless, due to marked study heterogeneity this was difficult at times, and was particularly evident when utilizing the OCEBM guidance, with previous systematic reviews disagreeing on studies’ LoE. Not only this, but some studies have a higher LoE, despite demonstrating less validity evidence than others. It is likely that guidelines require updating as surgical data science evolves. The application of methodological quality tools was found to be impractical for assessing AI studies, primarily as most are in their conceptual stage of development. Future research should focus on developing and piloting a new AI-specific study quality assessment tool.

## Conclusion

A large number of manual, automated and artificial intelligence tools in robotic surgery exist. There is huge variability in approach to assessment and the level of evaluation among all domains of robotic technical skill assessment, with few having been well validated. In addition, there is a lack of scope and most tools are presently only used within the research setting, despite the unmet need for both objective formative and summative tools to inform learning and accreditation, respectively. Collaboration between surgical societies, AI scientists and industry, with large high-quality studies and open data sets, appears the most efficient way forward to aid diffusion and implementation of objective assessment tools in clinical practice to enhance training and patient safety.

## Supplementary Material

znad331_Supplementary_Data

## Data Availability

Data for this review can be reproduced on reasonable request to the corresponding author.
