# Supplementary material for: Evaluation of objective tools and artificial intelligence in robotic surgery technical skills assessment: a systematic review
Source: Br J Surg. 2023 Nov 10;111(1):znad331. doi: 10.1093/bjs/znad331 (PMC10771126; doi:10.1093/bjs/znad331)
Supplement: znad331_Supplementary_Data [file znad331_supplementary_data.docx]

Evaluation of objective tools and artificial intelligence in robotic surgery technical skills assessment: A systematic review

**Authors:** Matthew WE. Boal^1,2,5^ MBChB (ORCID 0000-0002-7288-3354), Dimitrios Anastasiou^2,7^MEng, Freweini Tesfai^1,2^ MD/MSc, Walaa Ghamrawi^1^ MD, Evangelos Mazomenos^2,7^PhD, Nathan Curtis^3^PhD, FRCS, Justin W. Collins^4,5^MD, FRCS, Ashwin Sridhar^4,5^FRCS, John Kelly^4,5^FRCS, Danail Stoyanov^2,6^PhD, Nader K. Francis^1,5,8^ PhD, FRCS (ORCID 0000-0001-8498-9175)

**Affiliations**

1. The Griffin Institute, Northwick Park & St Marks’ Hospital, London, UK
2. Wellcome/ESPRC Centre for Interventional Surgical Sciences (WEISS), University College London (UCL), UK
3. Dorset County Hospital NHS Foundation Trust, Williams Avenue, Dorchester, UK
4. University College London Hospitals NHS Foundation Trust, UK
5. Division of Surgery and Interventional Science, Research Department of Targeted Intervention, UCL, UK
6. Computer Science, UCL, UK
7. Medical Physics and Biomedical Engineering, UCL, UK
8. Yeovil District Hospital, Somerset Foundation NHS Trust, UK

**Corresponding author.** Professor Nader Francis, The Griffin Institute, Y Block, Northwick Park & St Mark’s Hospital, London, HA1 3UJ. 02088693265, [n.francis@griffininstitute.org.uk](mailto:n.francis@griffininstitute.org.uk)

ORCID 0000-0001-8498-9175

**Supplementary Materials - Index**

| **Supplementary Figure**  Figure S1: Number of Machine and Deep Learning Studies 2011 to 2023  **Supplementary Tables** | *page 3* |
| --- | --- |
| Table S1: Database Search  Table S2: Modifiable Downs Black Checklist  Table S3: Global Rating Scale Tools Studies | *page 4*  *page 5*  *page 6-31* |
| Table S4: Procedure and Task-Specific Tools Studies | *page 32-44* |
| Table S5: Error-Based Tools studies | *page 45-56* |
| Table S6: Simulator Automated Performance Metrics | *page 57-78* |
| Table S7: Non-Simulator Automated Performance Metrics | *page 80-89* |
| Table S8: Artificial Intelligence Studies  Table S9 Summary of all other tools identified in assessment domains  Table S10: PRISMA Checklist | *page 90-92*  *page 93-95*      *page 96-99* |
|  |  |
| **References** | *page 100-118* |

**Supplementary Figure**

Figure S1: Number of Machine and Deep Learning Studies 2011 to 2023


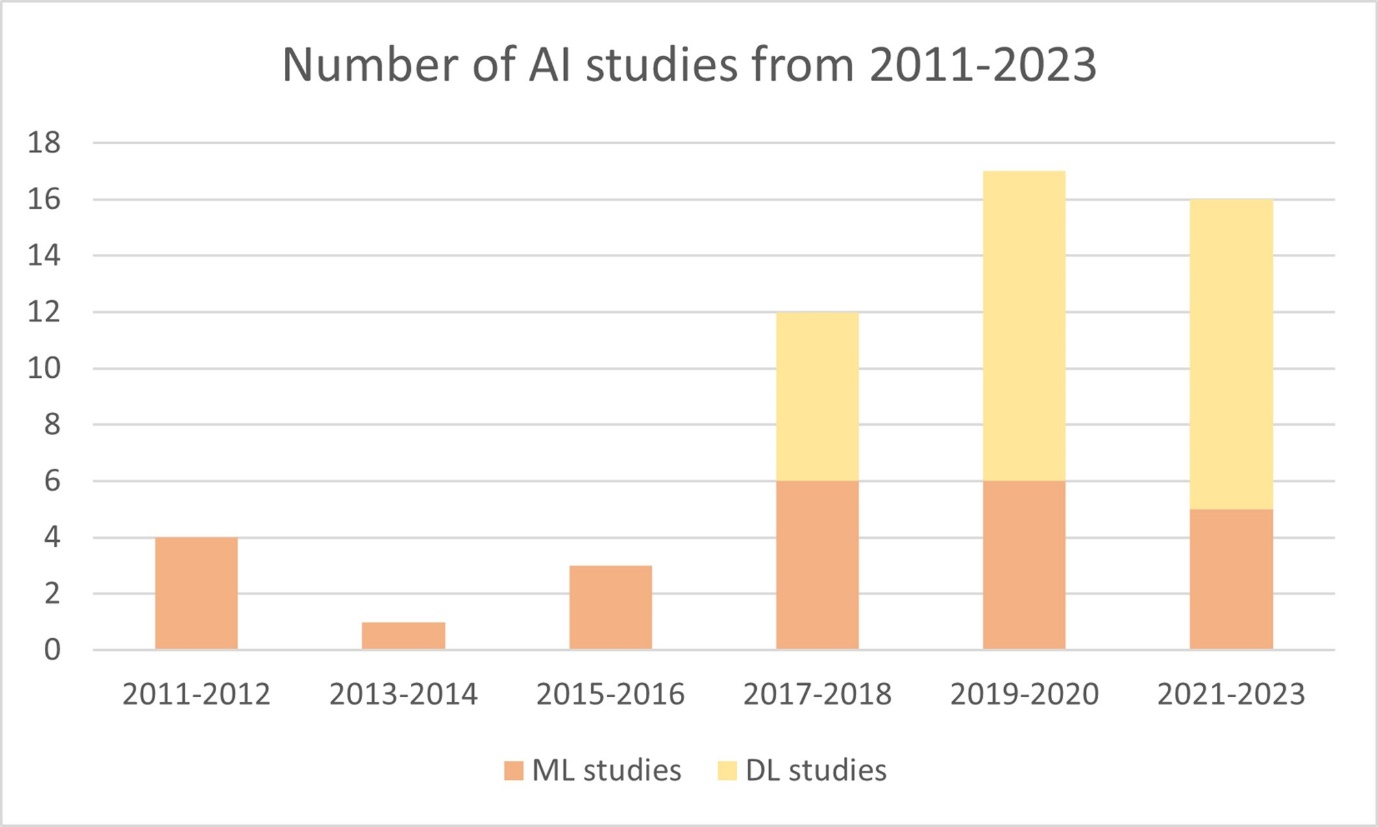


**Supplementary Tables**

Table S1: Database search

| Embase <1974 to 2022 February 22> and Ovid MEDLINE(R) ALL <1946 to February 22, 2022> = 1173 results minus duplicates  1 exp Robotics/ or exp Robotic Surgical Procedures/ or exp Laparoscopy/ or exp Minimally Invasive Surgical Procedures/ 853199  2 assess*.mp. [mp=ti, ab, hw, tn, ot, dm, mf, dv, kf, fx, dq, nm, ox, px, rx, ui, sy] 9730829  3 evaluat*.mp. [mp=ti, ab, hw, tn, ot, dm, mf, dv, kf, fx, dq, nm, ox, px, rx, ui, sy] 10171413  4 evaluate.mp. [mp=ti, ab, hw, tn, ot, dm, mf, dv, kf, fx, dq, nm, ox, px, rx, ui, sy] 3406091  5 exp Evaluation Study/ 342402  6 exp Validation Study/ 203268  7 (valid* or evaluat* or assess*).mp. [mp=ti, ab, hw, tn, ot, dm, mf, dv, kf, fx, dq, nm, ox, px, rx, ui, sy] 17934841  8 (objective or formative or summative).mp. [mp=ti, ab, hw, tn, ot, dm, mf, dv, kf, fx, dq, nm, ox, px, rx, ui, sy] 5060841  9 2 or 3 or 4 or 5 or 6 or 7 or 8 20097140  10 exp Clinical Competence/ or exp Simulation Training/ 178973  11 exp Motor Skills/ 108444  12 skill*.mp. [mp=ti, ab, hw, tn, ot, dm, mf, dv, kf, fx, dq, nm, ox, px, rx, ui, sy] 581119  13 surgical error.mp. or exp Medical Errors/ 274224  14 exp Near Miss, Healthcare/ 471  15 (proficien* or competen*).mp. [mp=ti, ab, hw, tn, ot, dm, mf, dv, kf, fx, dq, nm, ox, px, rx, ui, sy] 577834  16 10 or 11 or 12 or 13 or 14 or 15 1384816  17 1 and 9 and 16 20181  18 exp Robotic Surgical Procedures/ 28884  19 9 and 16 and 18 1525  20 remove duplicates from 19 1173  Web of Science 23/02/22 782 results  TS=("objective assessment" OR objective) AND TS=("robotic surgical procedures" OR "robotic surgical procedure" OR "robotic surgical skill" OR robot* OR "robotic assisted" OR "robotic-assisted") AND (((((TS=("objective assessment")) OR TS=(formative)) OR TS=(summative)) OR TS=(assess*)) OR TS=(evaluat*)) OR TS=(analys*) AND ((((TS=(skill)) OR TS=("robotic surgical skills")) OR TS=(competen*)) OR TS=(proficien*))  Pubmed 24/02/22 637 results  (((((((((((((((((robot*)) OR ("robotic-assisted")) OR ("robotic assisted")) OR ("robot assisted")) OR ("robot-assisted")) OR (laparoscop*)) OR ("laparoscopic assisted")) OR ("laparoscopic-assisted"))) OR ("minimally-invasive")) OR ("minimally invasive")) OR ("minimal access surgery")) OR ("minimal-access surgery")) AND ((evaluat*) OR (assess*))) AND ((((skill*) OR (proficien*)) OR (perform*)) OR (competen*))) AND ((train*) OR (education)) ) AND (objective) AND (tool*) |
| --- |

Table S2: Methodological quality assessment checklist modifiable Downs Black checklist^45^

|  | **Yes/No/UTD/NA** |
| --- | --- |
| **Is the hypothesis/aim/objective of the study clearly described?** |  |
| **Are the main outcomes to be measured clearly described in the introduction or methods section?** |  |
| **Are the characteristics of the patients included in the study clearly described?** |  |
| **Are the interventions (assessment) of interest clearly described?** |  |
| **Are the main findings of the study clearly described?** |  |
| **Does the study provide estimates of the random variability in the data for the main outcomes?** |  |
| **Have actual probability values been reported (e.g.0.035 rather than <0.05) for the main outcomes except where the probability value is less than 0.001?** |  |
| **External validity- Were the subjects asked to participate in the study representative of the entire population from which they were recruited?** |  |
| **Were the statistical tests used to assess the main outcomes appropriate?** |  |
| **Were the main outcome measures used accurate (valid and reliable)?** |  |
| **Modified DB checklist score (out of 10)** |  |

| Table S3: Global Rating Scale Tools | | | | | | | | | | | | | | | | | | | | | | | | |  |
| --- | --- | --- | --- | --- | --- | --- | --- | --- | --- | --- | --- | --- | --- | --- | --- | --- | --- | --- | --- | --- | --- | --- | --- | --- | --- |
| Study | Assessment Tools Compared | | Study type | | Participant  No. | | Expertise level | Setting | | Raters | | Task | | Test  Content | | Response process | | Internal Structure | | Relationship other variables | Consequence | | | L  O  E |  |
| GEARS | | | | | | | | | | | | | | | | | | | | | | | | |  |
| Goh 2012^145^ | GEARS | | Observational - Non-randomised experimental study | | 29 | | 25 Novices (PGY 4-6), 4 Experts (30+ robotic cases) | Operating theatre | | Expert | | RARP- SV dissection | | Expert consensus | | Trained surgeon, observer and trainee | | Excellent internal consistency ^Cα^ Excellent inter-rater reliability ^ICC^ | | Construct** |  | | | 2b |  |
| Hung 2013^163^ | GEARS & dVSS | | Observational - Non-randomised experimental study | | 49 | | 38 Novice/trainees (<30 cases), 11 Experts (30+ robotic cases) | Laboratory setting | | Expert | | In-vivo porcine suturing | |  | | Oriented, expert raters | |  | | Construct*** Strong correlation ^Sp^ with dVSS |  | | | 2b |  |
| Chen 2014^179^ | GEARS | | Observational - Non-randomised experimental study | | N/A | | Intermediate; Expert | Laboratory setting | | Expert (9 surgeons); Crowd sourced (409 Amazon Mechanical Turks & 67 Facebook users) | | Robotic suturing | |  | | Blinded expert raters Qualification and attention questions for inclusion as rater | |  | |  |  | | | 3 |  |
| Liang 2014^176^ | GEARS | | Randomised controlled trial | | 50 | | 40 Medical students, 10 Residents | Laboratory setting | | Expert | | 4 Dry tasks | |  | | Randomisation Blinded expert raters` Mentors oriented | |  | | No significant difference between intervention (20 mins mentoring) and control (no mentoring) groups overall |  | | | 2a |  |
| Ramos 2014^164^ | GEARS & dVSS | | Observational - Non-randomised experimental study | | 36 | | 24 Novices (medical students/surgical interns, no cases as primary surgeon) ; 12 Experts (Attendings and fellows 30+ cases as primary surgeon) | Laboratory setting | | Expert | | 3 Dry & VR tasks by Mimic technologies | |  | | Blinded expert raters | |  | | Construct*** Concurrent - Moderate correlation^Sp^ with dVSS |  | | | 2b |  |
| Kiely 2015^160^ | GEARS & GOALS + | | Randomised controlled trial | | 23 | | Residents and attendings | Laboratory setting | | Expert | | Pre- and post-test vaginal cuff suturing (dry model) | |  | | Blinded expert raters  Randomised, did not reach sample size | | High internal consistency ^K^ GEARS and GOALS+ | | Concurrent- No significant difference between GOALS+ and GEARS on multivariate analysis. |  | | | 2a |  |
| Aghazadeh 2015^148^ | GEARS | | Observational - Non-randomised experimental study | | 47 | | 9 Experts (residents and urologists >30 robotic cases primary surgeon), 14 Intermediate (Trainees with 5-29 cases), 24 Novices (<5 cases) | Laboratory setting | | Experts and self-evaluation (3 GEARS scores) | | In-vivo porcine task | | Expert consensus Live porcine tissue | | Blinded expert raters | | Excellent intra-rater consistency ^Cα^  Very strong inter-rater reliability^Sp^ | | Construct*** |  | | | 2b |  |
| Holst 2015^48^ | GEARS | | Observational - Non-randomised experimental study | | 12 | | 12 operative videos of varying skill levels | Laboratory setting | | Expert (7 experienced robotic surgeons), Crowd sourced (487 Amazon Mechanical Turk TM) (1 expert and 50 Crowd workers per video) | | Live porcine bladder closure | |  | | Qualification and attention questions for inclusion as crowd rater. Blinded raters | | Excellent inter-observer reliability ^Cα^ between groups  Good inter-rater reliability ^Cα^ between surgeon graders | |  |  | | | 2b |  |
| Hung 2015^99^ | GEARS & novel AR/VR simulator | | Randomised controlled trial | | 42 | | 15 Novices (no surgical training), 13 Intermediates (<100 robotic cases/any surgical training), 14 Experts (100+) | Operating theatre; Laboratory setting | | Expert | | 1. VR needle driving  2. In-vivo porcine robotic partial nephrectomy | |  | | Blinded expert rater | | Strong to very strong inter-rater reliability^Sp^ | | Construct** Concurrent - VR task and in-vivo on GEARS domains and total score**- *** |  | | | 2b |  |
| Valdis 2015^168^ | GEARS & dVSS | | Randomised controlled trial | | 19 | | Surgical trainees | Laboratory setting | | Expert | | Ex-vivo porcine harvest of internal thoracic artery and mitral annuloplasty | |  | | Blinded expert rater Randomised study | |  | | Construct*** dVSS VR intervention group, outperformed control group VR group comparable to experts after curriculum |  | | | 2a |  |
| Whitehurst 2015^149^ | GEARS | | Observational-prospective randomised study | | 20 | | Novice; Other: Robotic naive: 7 residents, 8 fellows, 5 attending surgeons | Laboratory setting | | Expert | | In-vivo porcine cystostomy closure (final test task) | |  | | Randomised groups  Blinded expert raters Raters completed workshop to improve agreement | | Excellent inter-rater reliability^Cα^ | |  |  | | | 2a |  |
| Chowriappa 2015^82^ | GEARS & Urethrovesical anastomosis evaluation score | | Randomised controlled trial | | 52 | | Novices (all residents or fellows <25 hours of robotic experience) | Laboratory setting | | Expert | | UVA | |  | | Randomised, powered study Blinded expert raters | | Inter-rater reliability GEARS 0.72 and UVA tool 0.68 (UTD statistical test used) | | Construct*-** Intervention outperformed control  Concurrent validity with UVA score |  | | | 2a |  |
| Raza 2015^81^ | GEARS & RACE | | Observational - Non-randomised experimental study | | 28 | | 10 Novices, 10 Beginners, 8 Experts | Laboratory setting | | Expert | | UVA | |  | | Blinded expert raters | |  | | Concurrent -Strong correlation ^Sp^ with RACE |  | | | 2b |  |
| Volpe 2015^73^ | GEARS | | Observational - Non-randomised experimental study | | 10 | | Expert; Other: Fellows | Operating theatre | | Expert | | RARP | |  | | Blinded expert raters | |  | |  | Arbitrarily defined good to excellent scores as 80-100% | | | 3 |  |
| Holst 2015^47^ | GEARS (modified) | | Observational - Non-randomised experimental study | | 5 | | 3 urology residents and 2 faculty | Laboratory setting | | Experts (3 surgeons), Crowd sourced (65 after exclusion Amazon.com Mechanical Turk TM) | | 5 dry lab robotic suturing videos | |  | | Blinded expert raters Qualification inclusion criteria as crowd rater | | Excellent inter-rater reliability^Cα^ | | Construct - no p value |  | | | 2b |  |
| Carter 2015^177^ | GEARS (modified) | | Randomised controlled trial | | 41 | | Residents | Laboratory setting | | Peer feedback | | Tubes VR exercise | |  | | Blinded peer feedback | |  | | Intervention group who received formative peer reviewed feedback using GEARS had significantly improved dVSS VR scores and outperformed control group |  | | | 2a |  |
| Gomez 2016^105^ | GEARS/OSATS combination | | Observational - Non-randomised experimental study | | 13 | | 8 Novice robotic surgeons, 5 Experienced robotic (50-2400 cases) | Laboratory setting | | Expert (4 experienced surgeons) | | 3 Dry lab tasks | |  | | Blinded expert raters Standardised method of rating | | Excellent internal consistency ^Cα^ Strong inter-rater reliability ^ICC^ | | Construct**  Negative correlation with vibration/forces and completion time*-** |  | | | 2b |  |
| Stefanidis 2016^58^ | GEARS, GOALS & CAT | | Observational - Non-randomised experimental study | | 32 | | Expert | Operating theatre | | Expert | | Lap/Robotic cholecystectomy and colectomy Lap /Robotic hysterectomy | |  | | Blinded expert raters | |  | | Identified technical deficiencies ranging from 35% decision making to 58% bimanual dexterity |  | | | 4 |  |
| Sanchez 2016^150^ | GEARS | | Observational - Non-randomised experimental study | | 15 | | 5 Novices (no robotics), 5 Intermediates (formal da Vinci console training), 5 Experts (15+ cases of robotic surgery) | Laboratory setting | | Not specified | | Robotic continuous suturing | |  | | Blinded raters | | High inter-rater reliability ^K^ | | Construct- Experts vs intermediates** and intermediates vs novice* |  | | | 2b |  |
| Aghazadeh 2016^146^ | GEARS, FIRST score (modified FLS metric) & dVSS | | Observational - Non-randomised experimental study | | 21 | | 17 Trainees (urology residents, fellows), 4 Experts (attending physicians) | Operating theatre; Laboratory setting | | Expert | | Endopelvic fascia dissection (RARP) | |  | | Live expert assessment then blinded expert video/audio review | | Strong inter-rater reliability ^Sp^ | | Concurrent-  GEARS & dVSS Very Strong ^Sp^  GEARS & FIRST score Very Strong ^Sp^ |  | | | 2b |  |
| Valdis 2016^169^ | GEARS | | Randomised controlled trial | | 40 | | Surgical trainees | Laboratory setting | | Expert | | Ex vivo-porcine harvest of internal thoracic artery and mitral valve annuloplasty | |  | | Blinded rater Randomised, powered study | |  | | Construct* but post training curriculum most had no significant difference between trainee and expert group |  | | | 1b |  |
| Powers 2016^49^ | GEARS (modified) | | Observational - Non-randomised experimental study | | 5 | | 5 resident and attending surgeons submitting 14 video clips | Operating theatre | | Experts (3 surgical content experts); Crowd sourced (30 Amazon Mechanical Turk raters) | | RAPN- Renal Hilar Dissection | |  | | Blinded video review of experts and crowd Trained both groups with GEARS use Crowd were selected after Human intelligence tasks and 95% approval rating | | Strong inter-rater reliability between average expert and crowd ratings ^P^  Low internal consistency of experts ^ICC^ | |  |  | | | 2b |  |
| Ghani 2016^64^ | GEARS & PACE | | Observational - Non-randomised experimental study | | 10 | | Surgeons providing videos | Operating theatre | | Crowd sourced: 23 peer surgeons, 784 Amazon Mechanical Turks | | RARP | |  | | Blinded raters | |  | |  |  | | | 4 |  |
| Ghani 2016^50^ | GEARS & RACE | | Observational - Non-randomised experimental study | | 12 | | Expert (MUSIC surgeons) | Operating theatre | | Expert (4 peer reviewers per video from 25 surgeons); Crowd sourced (Amazon Mechanical Turk from 30-55 reviewers) | | 1. One of four parts of RARP (bladder, apical dissection, nerve sparing, UVA) 2. UVA | |  | | Blinded raters: 2 minute introduction but no standardized training | | Low inter-rater reliability ^Kα^ amongst peer reviewers  Strong positive inter-rater reliability ^P^ peers and crowd sourced for GEARS and RACE  Both reviewer groups agreed on the rank order of bottom scoring videos, not the top. | |  | Pass or fail for surgeon question at end of video- subjective, not benchmarked, i.e.not assessment of GEARS | | | 2b |  |
| Bur 2017^151^ | GEARS | | Observational - Non-randomised experimental study | | 29 | | 20 residents, 5 fellows, 4 attending surgeons | Laboratory setting | | Expert (2 expert and 2 junior) | |  | |  | | Calibration of ratings | | Excellent inter-rater reliability^ICC^ | | Construct***  Weak evidence of concurrent validity - negative margins also related to expertise level, no correlational analysis |  | | | 2b |  |
| Ghani 2017^53^ | GEARS | | Observational - Non-randomised experimental study | | 29 | | Surgeons | Operating theatre | | Crowd sourced (C-SATS 285 raters providing 867 video ratings) | | RARP | |  | |  | |  | | Predictive validity- Higher GEARS = lower urethral catheter replacements***(OR=0.45), readmission rates** (OR=0.54) |  | | | 3 |  |
| Goldenberg 2017^180^ | GEARS | | Observational - Non-randomised experimental study | | 3 | | Expert surgeons | Operating theatre | | Expert (5 content experts); Crowd sourced (C-SATS 2142 crowd workers) | | Robotic assisted radical cystectomy (RARC) - Ureteral-ileal anastomoses | |  | | Blinded, trained crowd-raters used GEARS  Experts completed a subjective questionnaire | |  | | No predictive validity- No significant correlation between mean GEARS score and clinically significant uretero-ileal stricture Of note questionnaire by expert surgeons did not predict either |  | | | 3 |  |
| Ross 2017^167^ | GEARS | | Observational - Non-randomised experimental study | | 36 | | 22 Novices (medical students), 14 Intermediate/Expert surgeons | Laboratory setting | | Expert | | Dry lab UVA simulation | |  | | Expert rater | |  | | Construct*  Concurrent validity with NOn-Technical Skills for Surgeons (NOTSS) tool | Benchmarked using mean expert scores (2.9, presumably per domain, =pass ) | | | 4 |  |
| Vargas 2017^153^ | GEARS | | Randomised controlled trial | | 35 | | Experts, Novices (medical students) | Laboratory setting; Other: dVSS and live porcine | | Expert (4 experienced surgeons) | | 4 dVSS tasks until proficiency (intervention) vs no further training (control) Post test robotic suturing - live porcine model | |  | | Blinded expert raters x2 Randomised groups Allocation concealment | | Strong inter-rater reliability ^P^ | | Construct*** No difference in intervention vs control group mean GEARS and task times |  | | | 2a |  |
| Vernez 2017^51^ | GEARS | | Observational - Non-randomised experimental study | | 25 | | Medical students | Laboratory setting | | Expert (2 per clip); Crowd sourced (C-SATS 767 crowd assessments for robotic suturing) | | Robotic suturing Other tasks non robotic and different ax tools used (OSATS/GOALS) | |  | | Blinded crowd and expert raters Crowd trained to use tools and inclusion criteria to be allowed to rate | | Good inter-rater reliability Expert vs Crowd ^Cα^ Strong inter-rater reliability between experts ^P^ | |  |  | | | 2b |  |
| Dubin 2017^165^ | GEARS, dV-T & dVSS | | Randomised controlled trial | | 65 | | 42 Residents, 13 Fellow, 10 Attendings. 57% no previous robotic experience | Laboratory setting | | Crowd sourced (C-SATS verified raters, over 50 per video) | | dV-T and dVSS VR task- Ring and Rail 1 | |  | | C-SATS verified raters, 50 or more | |  | | Concurrent - Strong correlation GEARS total scores with dVT and dVSS^Sp^ GEARS separate domain correlations varied from very weak (efficiency and master workspace range) to very strong (efficiency and time to complete) |  | | | 2a |  |
| Mills 2017^181^ | GEARS & dVSS | | Observational - Non-randomised experimental study | | 10 | | Experts (attending robotic surgeons) | Operating theatre; Laboratory setting | | Expert | | 2 intra-operative clips | |  | | Blinded expert raters | |  | | No correlation between dVSS score and intraoperative GEARS score i.e. dVSS lacking predictive validity, GEARS no concurrent validity |  | | | 3 |  |
| Brown 2017^152^ | GEARS | | Observational - Non-randomised experimental study | | 37 | | Novice; Intermediate; Expert | Laboratory setting | | Expert | | Peg board transfer | |  | | Blinded raters | | Excellent inter-rater reliability in all GEARS domains^ICC^ | |  |  | | | 3 |  |
| Goldenberg 2017^162^ | GEARS & GERT | | Observational - Non-randomised experimental study | | 1 (24 cases) | | Expert | Operating theatre | | Expert | | RARP | |  | | Blinded expert rater | |  | | Concurrent- strong inverse correlation to GERT ^Sp^  Predictive validity- Higher GEARS score in the urinary continent group*. Total GEARS score, substep analysis of bladder neck dissection and UVA are independently predictive of continence OR 0.55, 95% CI 0.33-0.91 |  | | | 3 |  |
| Hung 2017^80^ | GEARS & Proficiency score | | Observational - Non-randomised experimental study | | 21 | | Robotic trainees - 11 residents, 10 fellows | Operating theatre | | Expert (Fellows, MIS faculty and Surgical Techs) | | RARP & RAPN | |  | | Orientation period trainees and raters. Expert raters x23 | |  | | Concurrent - Very strong overall association of GEARS with Proficiency Score ^Sp^ |  | | | 2b |  |
| Monda 2018^170^ | GEARS | | Observational - Non-randomised experimental study | | 24 | | 4 Beginners (medical students/interns), 14 Intermediate (residents), 3 Advanced (fellows), 3 Experts (attendings) | Laboratory setting | | Expert | | Robotic-Assisted Laparoscopic Partial Nephrectomy (RALPN) Training 3D silicone model | |  | | Blinded expert raters | |  | | Construct * - ** |  | | | 2b |  |
| von Rundstedt 2018^147^ | GEARS | | Observational - Non-randomised experimental study | | 22 | | 16 Trainees (residents and fellows), 6 Experts (faculty) | Laboratory setting | | Expert | | Ex-vivo porcine intracorporeal bowel anastomosis | |  | | Blinded expert raters | | Strong inter-rater reliability ^Sp^ | | Construct* |  | | | 2b |  |
| Hung 2018^157^ | GEARS & dVLogger | | Observational - Non-randomised experimental study | | 20 | | 10 Novices (<100 robotic cases) and 10 Experts (100+ cases) | Operating theatre | | Expert | | RARP (Seminal vesicle dissection and anterior UVA) | |  | | Blinded, independent raters | | Good inter-rater reliability ^ICC^ | | Construct***  Concurrent- GEARS with Kinematics very weak to moderate association ^Sp^ Event metrics no statistical association |  | | | 2b |  |
| Oh 2018^154^ | GEARS & dVLogger | | Observational - Non-randomised experimental study | | 11 | | 1 chief resident, 4 advanced robotics and lap fellows, 6 faculty attendings | Operating theatre | | Expert (4 expert), Crowd sourced (C-SATS 820 raters, each video 32-33 ratings) | | UVA | |  | | Blinded raters | | Very strong inter-rater reliability^Sp^ for Crowd and Expert average score with range moderate to v. strong in GEARS domains | | Concurrent/Construct- Total GEARS score discriminated APMs between higher and lower performing groups. |  | | | 2b |  |
| Guni 2018^161^ | GEARS & Task-specific checklist: robotic suturing | | Observational - Non-randomised experimental study | | 28 - 39 | | 22 Novices (no operative experience), 13 Experts (>50 RARPS) videos needle driving. 18 and 10 respectively participated for knot tying, 39 novices final checklist validation | Laboratory setting | | Expert | | UVA synthetic dry lab model | |  | | Independent expert rater | |  | | Construct - GEARS for needle driving**  - Task-specific checklist for needle driving and knot tying**  Concurrent-  Strong GEARS correlation with needle driving of checklist ^Sp^ Strong to very strong correlation ^Sp^ GEARS and checklist overall scores by expert observer for both sets of novice videos  No significant correlation of GEARS with Knot tying |  | | | 2b |  |
| Hoogenes 2018^83^ | GEARS, RACE & dVSS | | Randomised controlled trial | | 39 | | 23 Junior trainees (medical students and junior residents PGY 1-2), 16 Experienced trainees (senior residents PGY 3-5 and fellows) | Laboratory setting | | Expert | | UVA 3D printed model | |  | | Blinded expert raters  Randomised, powered study | | Excellent inter-rater reliability ^ICC^ | | dVSS group had higher GEARS (p=0.09) and UVA scores* |  | | | 1b |  |
| Goldenberg 2019^285^ | GEARS | | Observational - Non-randomised experimental study | | Retrospective from Goldenberg 2017 1 surgeon 24 cases | |  | Operating theatre | | Expert | | RARP | |  | | Blinded expert rater | |  | | Binary regression model including mean GEARS score independently predictive of continence at 3 months (OR 0.55, p=0.02, AUC 0.75) | Youden Index (optimal tradeoff between sensitivity and specificity) =0.35. Therefore, reverse engineered the regression formula to solve the required individual operative GEARS score for a predicted 65% probability of that patient being continent at 3 months, inputting their age, BMI and prostate volume into the formula. | | | 3 |  |
| Addison 2020^182^ | GEARS | | Observational - Non-randomised experimental study | | 68 | | 68 cases by 1 department of fellowship trained surgeons | Operating theatre | | Crowd sourced (C-SATS) | | Elective Robotic Sleeve Gastrectomy | |  | | Blinded raters, trained and regularly evaluated to determine rating reliability | |  | | No correlation of GEARS with operative time |  | | | 3 |  |
| Almarzouq 2020^166^ | GEARS | | Randomised, prospective study | | 14 | | Novice (robotic naïve residents) | Operating theatre; Laboratory setting | | Crowd sourced (C-SATS, 50 per video) | | RARP (bladder mobilization (all), UV anastomosis (senior residents)) compared with nine dVSSS tasks | | Construct validity from previous research guided choice of VR tasks | | Blinded raters  Randomised, powered study | |  | | Concurrent -Strong correlation GEARS efficiency domain during "energy & dissection" task on dVSS correlated strongly with GEARS efficiency during bladder mobilisation ^Sp^ . Moderate to Strong correlation GEARS force sensitivity in two VR tasks correlate with the same domain in bladder mobilization^Sp^. Very Strong correlation Total GEARS for two VR tasks with total GEARS for UVA ^Sp^ |  | | | 2a |  |
| Bendre 2020^178^ | GEARS | | Observational - Non-randomised experimental study | | 11 | | 8 residents, 3 faculty | Laboratory setting | | Crowd sourced (C-SATS 30-34 per video) | | 3D printed silicon model- Dismembered pyeloplasty | |  | | Blinded crowd raters with 30-34 reviewers each video | |  | | No construct- attendings vs. junior residents (p=0.066).  Poor to moderate correlation (UTD statistical test) of level of training with GEARS scores 1st and 2nd attempt respectively. |  | | | 2b |  |
| Raison 2020^155^ | GEARS | | Observational - Non-randomised experimental study | | 43 | | Novice (minimal open/lap experience and no robotic experience) | Laboratory setting | | Expert | | VR vs. Dry lab training assessments: - Endowrist manipulation & clutching  - Suturing - UVA dry model assessment | |  | | Expert raters | | Excellent inter-rater reliability ^ICC^ | | Greater increase in GEARS for Dry lab group vs VR* |  | | | 2b |  |
| Sarcona 2020^175^ | GEARS | | Observational - Non-randomised experimental study | | 4 | | Fellowship trained urologists | Operating theatre | | Crowd sourced (C-SATS) | | 125 cases prostate, bladder, upper tract, other | |  | |  | |  | | Predictive validity- C-SATS GEARS scores strongly negatively correlated with Total number of complications and Clavien 3+ complications ^P^ |  | | | 4 |  |
| Timberlake 2020^171^ | GEARS | | Observational - Non-randomised experimental study | | 25 | | 4 Novices (residents), 6 Experts (fellows and faculty) | Laboratory setting | | Expert | | Robotic pyeloplasty-silicone model | |  | | Blinded expert raters | |  | | GEARS- Construct** Time - Construct* Concurrent GEARS and Anastomotic leak plus construct of Anastomotic leak: Flow rates also differed between experts and novices* |  | | | 2b |  |
| Satava 2020^15^ | GEARS & Task-specific metrics | | Randomised controlled trial | | 99 | | Novices (residents, fellows, attendings- less than 5 robotic cases), Experts (attendings, minimum 50 robotic cases primary surgeon) | Laboratory setting | | Expert | | Pre- and post-test avian model reflecting FRS tasks Groups:  - Experimental- physical FRS dome, dVT and dVSS VR FRS - Control (normal local curriculum) | |  | | Randomised, powered study  Blinded expert raters Raters had calibration scoring sessions prior to assessment to harmonize approach | | GEARS- Moderate IRR (percent-agreement)  Task-specific metrics tool -High IRR | |  |  | | | 1b |  |
| Witthaus 2020^84^ | GEARS, RACE & Clinically Relevant performance Metrics (CRPMS) | | Observational - Non-randomised experimental study | | 14 | | 9 Novices (caseload <50), 5 Experts (caseload >500) | Laboratory setting | | Expert | | Nerve Sparing RARP 3D printed hydrogel model | |  | | Blinded expert raters | |  | | GEARS Construct** and  GEARS Concurrent Strong to very strong inverse correlation of Total GEARS and force sensitivity domain and nerve forces of CRPMS^Sp^ |  | | | 2b |  |
| Fukuoka 2021^156^ | GEARS | | Observational - Non-randomised experimental study | | 9 videos | | Varying skill levels | Operating theatre | | Expert and novice 42 urologists; Crowd-sourced: 19 paramedics, 73 medical students and 28 non-medics | | UVA | |  | | Blinded reviewers Perfect score video watched first as benchmark | | Strong inter-rater reliability ^F^ between urologist group compared to other groups | |  |  | | | 2b |  |
| Yu 2021^158^ | GEARS & assistant OSATS (aOSATS) | | Observational - Non-randomised experimental study | | 36 | | 14 console surgeons, 22 bedside assistants | Operating theatre | | Expert | | RARP (92 videos) | |  | | Blinded expert raters | | GEARS and aOSATS acceptable inter-rater reliability ^Cα^ | | Concurrent validity- Weak correlation GEARS and aOSATS^Sp^ |  | | | 3 |  |
| Raison 2021^173^ | GEARS & The RobotiX Mentor | | Randomised controlled trial | | 26 | | Novice (no experience in robotic surgery) | Laboratory setting | | Not specified | | RARP cadaver task (summative task at the end of study) | |  | | Blinded rater | |  | | Construct** Higher GEARS scores in VR training groups compared to control |  | | | 2a |  |
| Kelly 2021^159^ | GEARS, dVSS & dVLogger | | Randomised controlled trial | | 34-41 | | Surgeons: Junior, Senior and Attending | Operating theatre; Laboratory setting | | Expert (7 surgeons); Crowd sourced (C-SATS) | | 1. 5 groups different warm up curricula, either one of or all 4 tasks on dVSS VR Each of the 5 groups had bimonthly or monthly attempts for 6 sessions and 2. Operating room surgery - 434 recorded,  GEARS ratings of 45 videos  347 with kinematics included | |  | | Blinded experts and C-SATS Random allocation | | Poor inter-rater reliability between experts ^ICC^ | | 1.. Kinematics and events metrics not significantly associated with GEARS score or warm-up  2. Type of robot Xi vs Si no difference in GEARS score |  | | | 2a |  |
| Ghazi 2021^52^ | GEARS & Clinically Relevant Objective Metrics (CROMS) | | Observational - Non-randomised experimental study | | 43 (30 videos assessed with GEARS) | | 27 Novices (1-30 upper tract robotic cases), 16 Experts (>150) | Laboratory setting | | Expert; Crowd sourced (C-SATS) | | Robot-assisted partial nephrectomy (RAPN) simulation 3D model | |  | | Blinded raters | |  | | Construct*** GEARS and CROMS (except PSM)  Concurrent- strong to highly correlation^P^ between CROMS (exc. PSM) and total GEARS |  | | | 2b |  |
| Butterworth 2021^172^ | GEARS (modified) | | Observational - Non-randomised experimental study | | 17 | | All senior surgeons. 4 Novices (<5 robotic procedures), 8 Intermediates (5-29), 5 Experts (30 +) | Laboratory setting | | Expert | | Versius, CMR training programme: Wet lab core skills | |  | | 2 expert raters scored most Trained to use GEARS | |  | | Construct (no p value) |  | | | 3 |  |
| Chow 2021^71^ | GEARS | | Observational - Non-randomised experimental study | | 12 | | Novice (residents) | Laboratory setting | | Expert (Five blinded fellowship trained robotic surgery faculty members) | | Ex vivo porcine simulation RAPN | |  | | Blinded expert raters | | Excellent inter-rater reliability ^ICC^ | | Mean GEARS scores improved with each session** correlating with residents' confidence and time improving |  | | | 3 |  |
| Gómez Ruiz 2022^77^ | GEARS &Task Performance Metrics: RA-LAR | | Observational - Non-randomised experimental study | | 14 | | Colorectal surgeons from ESCP working group- 5 Novice (<30 RA-LAR procedures), 9 Experienced (30+ RA-LAR procedures) | Operating theatre | | Expert (2 consultant colorectal surgeons >50 robotic resections) | | Robotic anterior resection | |  | | Blinded, independent, expert raters  Trained to use both tools until using consistently with IRR >0.8 | | IRR (agreement/agreement+disagreements)  Task performance metrics IRR 0.94 and GEARS IRR 0.49, | | Construct- Task performance metrics* -** with better discrimination (58-64%) than GEARS (15-41%) of higher performers. |  | | | 2b |  |
| Tarr 2022^174^ | GEARS | | Observational - Non-randomised experimental study | | 26 | | 17 Trainees s(PGY 5-7), 9 Experts (fellowship trained, routinely performing robotics) | Laboratory setting | | Expert | | Robotic Sacrocolpopexy (simulation model) | |  | | Blinded expert raters Standardised method for assessment including human factors specialist | |  | | Construct** |  | | | 2b |  |
| OSATS | | | | | | | | | | | | | | | | | | | | | | | | |  |
| Hernandez 2004^189^ | OSATS | | Observational - Non-randomised experimental study | | 13 | | 6 Experienced group (>100 lap chole and clinical practice intracorporeal knot tying), 7 non-experienced (<50 lap choles and no advanced lap experience) | Laboratory setting | | Expert | | Synthetic small bowel anastomoses (Limbs and Things) | |  | | Blinded expert raters | | Good inter-rater reliability ^Cα^ | | OSATS scores improved with successive attempts** No construct validity when comparing groups, OSATS higher in experienced but not significant (p=0.39) | Competent 24 or more out of 40 in OSATS | | | 2b |  |
| Korets 2011^193^ | OSATS (modified) | | Observational - Non-randomised experimental study | | 16 | | Urology residents: 10 Novices (<50 robotic cases), 6 Intermediates (50+) | Laboratory setting | | N/A | | Dry model- Ring rail and knot tying | |  | | Blinded rater | |  | | Construct* Baseline evaluation no difference in groups, 2nd post-training evaluation: OSATS higher in group 1 & 2 (training curriculum) compared to group 3 (no VR curriculum) |  | | | 2b |  |
| Curry 2012^187^ | | | OSATS | | Observational - Non-randomised experimental study | | 12 | 8 Residents, 4 Experts (practicing robotic surgeons) | Laboratory setting | Expert | | Module 1- 4 dry lab tasks (suturing, manipulation, dissection, transection) Module 2 patient cart set up Module 3 ex-vivo porcine TORS | |  | | Expert raters | | Fair to moderate inter-rater reliability ^CK^ | | Construct*** | | |  | 2b | |
| Lee 2012^197^ | | | OSATS (modified) | | Observational - Non-randomised experimental study | | 3 | >5 years experience, 4 months and none | Laboratory setting | Not specified | | In-vivo rat femoral artery microsurgical anastomoses | |  | | Independent rater | |  | | Concurrent- OSATS weakly correlated^Sp^ with anastomosis patency* and quality** | | |  | 3 | |
| Tunitsky 2013^54^ | | | OSATS, GOALS & modified Surgical Skill Index | | Observational - Non-randomised experimental study | | 21 | 12 Trainees (residents and fellows), 9 Experts | Laboratory setting | Expert | | Ureteral Anastomosis Simulation Model | |  | | Expert raters Powered study | |  | | Construct- procedural experts vs robotic (p=0.05) or trainees**, but no difference between robotic experts and trainees (p=0.06)  GOALS distinguished expert level better than OSATS | | |  | 2b | |
| Egi 2013^90^ | | | OSATS (GRS and task-specific) | | Observational - Non-randomised experimental study | | 12 | Intermediate; Expert | Laboratory setting | Expert | | Bench model suturing | |  | | Blinded expert raters | | High inter-rater reliability ^Cα^  OSATS GRS 0.891  OSATS checklist 0.803 | | Concurrent: Moderate correlation OSATS suturing score to dV-T suture sponge^Sp^ | | |  | 2b | |
| Alemozaffar 2014^188^ | | | OSATS | | Observational - Non-randomised experimental study | | 20 | 10 Novices (<10 RARP), 10 Experts (>50 RARP) | Laboratory setting | Expert | | RARP ex-vivo porcine model | |  | | Blinded expert raters Powered study | | Good inter-rater reliability^Cα^ | | Construct*** | | | Quality of nerve sparing, anastomosis.  Hypothesised mean OSATS category scores 3.5 novice and 4.5 expert- concordance with results of mean OSATS | 2b | |
| Vogell 2014^191^ | | | OSATS | | Observational - Non-randomised experimental study | | 8 | Residents | Operating theatre | Expert | | Robotic vaginal cuff closure | |  | | Blinded expert raters | |  | | Concurrent OSATS and predictive validity dVSS VR: Strong correlation between rate of change in OR OSATS scores and total amount of simulation ^P^ | | |  | 3 | |
| Tarr 2014^183^ | | | OSATS | | Observational - Non-randomised experimental study | | 165 pre-test, 99 post-test | Residents | Laboratory setting | Expert | | 4 dry model tasks (ROSATS curriculum) | |  | |  | |  | | OSATS scores improved** -*** except transection (p=0.21).  Weak negative correlation with task-specific errors ^P^ (no p value).  Moderate to high positive correlation with a task-specific Likert scale ^P^ | | |  | 2b | |
| Sobel 2016^62^ | | | OSATS (GRS & task-specific) | | Observational - Non-randomised experimental study | | 16 | PGY 2- 6 with no previous standardised robotic training, 4 expert robotic surgeons (who currently perform TORS) | Laboratory setting | Expert | | Ex-vivo porcine model TORS. 2 tasks: robotic radical tonsillectomy and base of tongue (BOT) resection | |  | | Blinded expert rater | |  | | Construct**pre- curriculum  Comparable scores post-curriculum between trainees and experts | | | Composite pass score developed based on expert metrics | 2b | |
| Willems 2016^88^ | | | OSATS (GRS and task-specific) | | Observational - Non-randomised experimental study | | 2 | No experience in microsurgery/robotics | Laboratory setting | Independent rater | | Simulation microvascular anastomosis (classic microsurgery vs robotic) | |  | | Independent rater  Powered study | |  | | Manual outperformed robotic anastomosis* | | |  | 3 | |
| Hogg 2016^86^ | | | OSATS (modified) | | Observational - Non-randomised experimental study | | 133 | Expert | Operating theatre | Expert (international clinical fellows) | | Robotic pancreaticojejunostomy during a pancreaticoduodenectomy | |  | | Blinded expert raters Trained to use OSATS | | Intra-rater reliability: Strong to very strong ^Sp^  for OSATS and PJV for both graders  Inter-rater reliability ^Sp^: Moderate for OSATS  Weak for PJV | | Predictive validity - Higher PJV/OSATS scores significantly correlated with lower rate POPF*  - Lower PJV score significantly associated with increased rate POPF* - Subjective expert prediction no correlation to POPF | | |  | 3 | |
| Vedula 2016^198^ | | | OSATS | | Observational - Non-randomised experimental study | | 18 | 14 novices (surgical trainees) and 4 experts (attendings) | Laboratory setting | Expert | | close incision: suture throw, and surgeon's knot | |  | | Expert rated | |  | | Construct- Yes | | | Arbritrary cut offs >22 expert, 15-22 intermediate, 14 or below novice | 3 | |
| Gomez 2016^105^ | | | OSATS/GEARS combination | | Observational - Non-randomised experimental study | | 13 | 8 Novice robotic surgeons, 5 Experienced robotic (50-2400 cases) | Laboratory setting | Expert (4 experienced surgeons) | | Dry model (156 videos): Peg transfer, needle pass, suturing | |  | | Blinded expert raters Standardised method of rating | | Excellent internal consistency ^Cα^ Strong inter-rater reliability^ICC^ | | Construct**  Negative correlation with vibration/forces and completion time*-*** | | |  | 2b | |
| Knab 2017^184^ | | | OSATS | | Observational - Non-randomised experimental study | | UTD | Surgical fellows | Operating theatre; Laboratory setting | Expert | | Dry model suturing and specialty-specific anastomoses | |  | |  | |  | | Improved OSATS scores with more attempts*** | | | 3 experts used to obtain average "gold standard" metrics | 3 | |
| Tam 2017^185^ | | | OSATS | | Observational - Non-randomised experimental study | | 15 | Surgical oncology fellows | Laboratory setting | Expert | | Dry biotissue models (pancreatico-, gastro-, hepaticojejunostomy | |  | | Blinded expert raters | | Excellent inter-rater reliability^ICC^ | | Construct* on first attempts | | | Proficiency benchmarked by 3 experts | 2b | |
| Rice 2019^186^ | | | OSATS | | Observational - Non-randomised experimental study | | 36 | 6 residents, 19 HPB fellows and 11 attendings | Laboratory setting | Expert (2 expert ratings); Crowd sourced (premedical students) | | 3 dry model anastomosis drills  performed in Whipple's (HJ/GJ/PJ) | |  | | Crowd trained and tested on use of OSATS to improve selection  Blinded expert raters | | Excellent inter-rater reliability of selected crowd correlation with experts^ICC^ | |  | | |  | 2b | |
| Moncayo 2020^196^ | | | OSATS | | Randomised crossover study design | | 12 | Novice | Laboratory setting | Senior reviewers | | Dry model drills "Transfer the Plot (TP)" and "Thread the Rings (TR)" | |  | | Randomised study  Independent expert raters | |  | | OSATS scores better overall and for 5/6 domains in robotics vs laparoscopy* | | |  | 2a | |
| Chen 2021^192^ | | | OSATS | | Prospective single armed study non randomised, compared to RCT of laparosopy | | 2 | Experts (>50 RTG and LTG experience) | Operating theatre | Expert | | Laparoscopic and Robotic Total Gastrectomy | |  | | Blinded, trained expert rater | |  | | RTG vs LTG***: - higher OSATS score - lower blood loss - higher extra perigastric LN yield  Moderate correlation between total OSATS and cognitive task load scores^Sp^  No significant difference in post op morbidity | | |  | 2b | |
| Ahmad 2021^194^ | | | OSATS | | Observational - Non-randomised experimental study | | 30 | Senior Fellows - Experience ranked none to high | Laboratory setting | Not specified | | 19 biotissue drills (Inanimate Reality- IR) | |  | | Blinded raters | |  | | Construct*** of combined scoring system including OSATS i.e. correlation between Performance and Experience rank ^P^ | | |  | 2b | |
| Zwart 2021^195^ | | | OSATS | | Randomised controlled trial | | 60 | 36 expert surgeons, 24 residents (pooled analysis 2 RCTs) | Laboratory setting | Expert | | Robotic vs 3D/2D laparoscopy pancreatico- and hepatico-jejunostomy biotissue models- 220 anastomoses (80 were robotic) | |  | | Randomised  Blinded, trained rater | |  | | Robotic OSATS higher ratings than lap groups***and shorter operative time | | |  | 2a | |
| Hutchinson 2022^190^ | | | OSATS | | Observational - Non-randomised experimental study | | 8 (JIGSAWS) | Self-proclaimed Expert (>100 hours robotic experience), Intermediate (10-100), Novice (<10 hours) or defined by OSATS scores | Laboratory setting | Expert | | JIGSAWS data set | |  | | Independent expert rater from JIGSAWS data set | |  | | Concurrent validity- Negative correlation between OSATS and procedural error frequency (r= -0.51, p<0.001, UTD statistical test used) in suturing.  No correlation for needle passing | | |  | 3 | |
| Willuth 2022^63^ | | | OSATS (GRS and task-specific) | | Randomised crossover study | | 40 | Novice (medical students) | Laboratory setting | Expert | | Ex vivo porcine cholecystectomy (Lap and robotic) | |  | | Randomised Blinded raters | |  | | Robotic OSATS total (combined), OSATS GRS and OSATS task-specific scores higher than lap* | | |  | 2a | |
| GOALS | | | | | | | | | | | | | | | | | | | | | | | | | |
| Hung 2012^200^ | | | GOAL+ and dVSS | | Randomised controlled trial | | 24 | Robotic surgery trainees | Laboratory setting | Expert | | 3x ex vivo porcine tasks (bowel resection, cystostomy repair, partial nephrectomy) | |  | | Blinded expert, oriented raters  Randomised, powered study | |  | | Concurrent - Strong correlation GOALS and overall dVSS metrics ^Sp^, range of moderate to strong correlations for separate dVSS metrics and GOAL+ domains ^Sp^. | | |  | 1b | |
| Hung 2012^199^ | | | GOAL+ | | Observational - Non-randomised experimental study | | 46 | 24 Novices (no robotic experience), 9 Intermediate (1-99 robotic cases), 13 Experts (100+) | Laboratory setting | Expert | | RAPN ex-vivo porcine simulation | |  | | Blinded expert raters | |  | | Construct- Experts vs. intermediates** and novices*** | | |  | 2b | |
| Tunitsky 2013^54^ | | | GOALS, OSATS & modified Surgical Skill Index | | Observational - Non-randomised experimental study | | 21 | 12 Trainees (residents and fellows), 9 Experts | Laboratory setting | Expert | | Ureteral Anastomosis Simulation Model | |  | | Expert raters Sample size calculation | |  | | Construct - procedural experts vs. robotic experts* and trainees**. Robotic experts vs trainees (p=0.05)  GOALS better classifier than OSATS and modified SSI in this study | | | GOALS- Pass mark defined by contrasting groups method of standard setting (20/25 - 80%) | 2b | |
| Vaccaro 2013^92^ | | | GOALS | | Randomised controlled trial | | 18 | Residents | Laboratory setting | Expert | | Ex vivo chicken model dissection and suturing | |  | | Blinded expert raters Randomised, powered study | |  | | No significant difference between intervention (simulator training) and control group | | | Arbitrary pass/fail set by experts: based on score (15+) and if moderator had to intervene | 2b | |
| Culligan 2014^201^ | | | GOALS | | Observational - Non-randomised experimental study | | 23 | 14 Study surgeons (attendings, no prior robotic experience), 4 Controls (surgeons), 5 Experts (robotic surgeons) | Operating theatre; Laboratory setting | Expert | | Live porcine and human hysterectomy | |  | | Blinded expert raters | |  | | Construct*** | | |  | 2b | |
| Kiely 2015^160^ | | | GOALS+ , GEARS and dVSS | | Randomised controlled trial | | 23 | Residents and attendings | Laboratory setting | Expert | | Pre- and post-test vaginal cuff suturing (dry model) | |  | | Blinded expert raters  Randomised, did not reach sample size | | High internal consistency ^K^ GOALS+ 0.84, GEARS 0.85 | | Concurrent -Yes Moderate correlation GOALS+ with dVSS VR suture sponge 1 ^Sp^  No significant difference between GOALS+ and GEARS on multivariate analysis | | |  | 2a | |
| Stefanidis 2016^58^ | | | GOALS, GEARS and CAT | | Observational - Non-randomised experimental study | | 32 | Expert | Operating theatre | Expert | | Lap/robotic cholecystectomy and colectomy  Lap/robotic hysterectomy | |  | | Blinded expert raters | |  | |  | | |  | 4 | |
| Other Global Rating Scale Tools | | | | | | | | | | | | | | | | | | | | | | | | | |
| Liu 2018^205^ | | | ARCS | | Observational - Non-randomised experimental study | | 15 | Surgeons: Novice (0 robotic procedures), Intermediate (1-100), Experienced (>100) | Laboratory setting | Expert | | 3 tasks - ex vivo porcine | | Consensus development by 3 expert da Vinci Surgical System professional trainers | | Blinded expert raters | | Moderate to high inter-rater reliability in all domains^K^ | | Construct in 5/6 domains*-** (excluding basic energy pedal skills) | | |  | 2b | |
| Tunitsky 2013^54^ | | | modified Surgical Skill Index, GOALS & OSATS | | Observational - Non-randomised experimental study | | 21 | 12 Trainees (residents and fellows), 9 Experts | Laboratory setting | Expert | | Ureteral Anastomosis Simulation Model | |  | | Expert raters (live assessment by one of two) Powered study | |  | | Construct validity for procedural experts vs robotic* and trainees**, but no difference between robotic experts and trainees (p=0.3) | | |  | 2b | |
| Siddiqui 2014^202^ | | | R-OSATS | | Observational - Non-randomised experimental study | | 105 | 83 residents, 9 fellows, 13 faculty. | Laboratory setting | Expert | | 5 dry drills (tower transfer, rollercoaster, big dipper, train tracks, figure of eight) | |  | | Blinded orientated expert raters | | Acceptable to good inter-rater reliability^Cα^ Very strong intra-rater reliability^Sp^ | | Construct** | | |  | 2b | |
| Polin 2016^203^ | | | R-OSATS | | Observational - Non-randomised experimental study | | 60 videos | Not specified | Laboratory setting | Expert (3 surgeons); Crowd sourced (448 Amazon Mechanical Turk raters) | | 5 dry lab tasks 1) Tower Transfer 2) Rollercoaster 3) Big Dipper 4) Train tracks (continuous suture) 5) Figure of 8 suture | |  | | Blinded, trained crowd raters who pass qualification criteria Blinded expert reviewers | | Expert evaluator: Moderate to good Intra-rater reliability^ICC^  Inter-rater reliability: Crowd evaluators strong correlation with experts^P^  Bootstrapping revealed minimum crowdworker assessments per video is 15 to maintain high correlation with experts | |  | | |  | 2b | |
| Siddiqui 2016^55^ | | | R-OSATS | | Observational - Non-randomised experimental study | | 57 | 35 inexperienced (PGY1-2) junior residents, 22 senior experienced (9 fellows, 13 faculty) | Laboratory setting | Expert | | 5 dry tasks (tower transfer, rollercoaster, big dipper, train tracks, figure of eight) | |  | | Raters calibrated | | Very high inter-rater reliability (0.92-0.98 rWGindex) | |  | | | Modified Angoff method with content experts to set threshold competency scores of 14/20 minimum cutoff score per drill | 2b | |
| Newcomb 2018^204^ | | | R-OSATS & dVSS | | Observational - Non-randomised experimental study | | 30 | 19 residents, 7 fellows, 4 faculty | Laboratory setting | Expert; Crowd sourced (3 C-SATS expert reviewers) | | 5 VR and Dry lab equivalent tasks | |  | | Blinded expert raters | | Excellent inter-rater reliability ^ICC^ | | Construct: VR drills between residents and fellows and residents and faculty**. No significant difference between fellows and faculty.  Concurrent: VR and Dry lab model equivalent showed strong correlation with overall score of VR and R-OSATS^Sp^. Moderate to strong correlation in individual drills ^Sp^ | | |  | 2b | |
| Gerull 2019^206^ | | | RO-SCORE & dVSS | | Observational - Non-randomised experimental study | | 31 | Novice (residents with no robotic experience) | Operating theatre; Laboratory setting | Expert | | "Participated in a live robotic-assisted laparoscopic surgical (RALS) case" | |  | | Expert evaluator, not blinded | |  | | Average scores improved from pre- to post VR curriculum RO-SCORES*** Correlated with reduction in NTLX (National Aeronautics and Space Administration-Task Load Index) domains*** | | |  | 3 | |
| Selber 2014^207^ | | | SARMS | | Observational - Non-randomised experimental study | | 10 | Plastic surgeons with varying experience | Laboratory setting | Expert | | Mircrosurgical anastomosis of synthetic 3mm vessel | |  | | Blinded expert raters | | Good to excellent internal consistency ^Cα^ Good inter-rater reliability ^CK^ | | Construct* | | |  | 3 | |

| Supplementary Table Table S4: Procedure- and Task-Specific Tools Procedure- and Task-Specific Tools | | | | | | | | | | | | | |
| --- | --- | --- | --- | --- | --- | --- | --- | --- | --- | --- | --- | --- | --- |
| Study | Assessment Tools Compared | Study type | Participant  No. | Expertise level | Setting | Raters | Task | Test  Content | Response process | Internal Structure | Relationship other variables | Consequence | L  O  E |
| Schmidt 2022^85^ | Anastomosis-OSATS  (A-OSATS) | Other: Delphi then video analysis. | 19 Delphi  36 to 40 OR videos | 8 Novices (0 MIS anastomoses), 24 Intermediate (1-10), 8 Expert (11+). | Operating theatre; Other: Delphi consensus | Expert | 41 minimally-invasive stapled intestinal anastomoses performed 27 lap vs 14 robotic | Delphi consensus and thorough literature review | Blinded expert raters, each rated the video twice. Randomised | Strong intra rater reliability^Sp^ Excellent interrater reliability ^ICC^ | Concurrent: very strong correlation with OSATS GRS^Sp^  Construct* | Arbitrary based on previous studies ≤18 considered novices, 19-27 intermediates, ≥28 experts | 2a |
| Hussein 2018^56^ | Cystectomy Assessment and Surgical Evaluation  (CASE) | Other: Delphi process | 10 | Intermediate; Expert | Operating theatre; Other: Delphi consensus | Expert | Robotic-assisted radical cystectomy (RARC) | Delphi consensus | Expert panel for content validity Blinded raters for reliability | Inter-rater reliability- Yes (linearly weighted percent agreement 0.68-0.81) | Failed to show construct validity: experts outperformed trainees but not with statistical significance |  | 2b |
| Stefanidis 2016^58^ | Competency Assessment Tool (CAT)  Multiple ops | Observational - Non-randomised experimental study | 32 | Expert | Operating theatre | Expert | Lap and robotic cholecystectomy and colectomy Lap and robotic hysterectomy |  | Blinded expert raters |  | Identified technical deficiencies ranging from 35% decision making to 58% bimanual dexterity |  | 4 |
| Petz 2016^59^ | Competency Assessment Tool (CAT)  For colorectal and basic robotic skills | Consensus meeting | UTD | Expert | Other: Consensus meeting | N/A | Robotic colorectal surgery | Weakly yes- not assessed formally |  |  |  |  | 4 |
| Moloney 2021^87^ | Competency Assessment Tool (CAT)  For Sentinel Lymph Node Dissection (SLND) | Other: Delphi then video analysis. | 35 | Expert | Other: Delphi consensus | Expert | SLND in endometrial cancer | Face/Content- Delphi consensus using hierarchical task analysis | Blinded expert raters | High internal consistency ^Cα^ | Contrast validity*** |  | 3 |
| Vanstrum 2021^96^ | Dissection Assessment for Robotic Technique  (DART) | Observational - Non-randomised experimental study | 14 Delphi, 10 blinded reviewers of 46 videos | Trainees (<100 robotic cases), experts (>100 cases)- referenced | Operating theatre; Other: Delphi consensus | Expert | Robotic Pelvic Lymph Node or Seminal Vesical Dissections (RARP) | Delphi consensus | Blinded reviewers with training round and 2 consensus building session prior to rating | Fair to Very Good ^CK^ inter-rater reliability  3-point Likert scale seemed to be more reliable compared to 5 point | Construct*** |  | 2b |
| Panteleimonitis 2018^60^ | Global Assessment Scale (GAS) | Observational - Non-randomised experimental study | 3 | Surgeons formally lap trained in colorectal surgery, no robotics | Operating theatre | Expert | Robotic anterior resection |  | Filled immediately at the end of each case  Blinded summative assessment after this |  |  | CUSUM score target score of 5 is equitable to independent/competent performance  In this study, ten supervised cases sufficient until these 3 surgeons able to perform robotic anterior resection | 3 |
| Eddahchouri 2022^61^ | Minimally-Invasive Esophagectomy (MIE) | Delphi consensus | 31 | Expert (23 and 27 1st and second rounds) | Delphi consensus | N/A |  | Delphi consensus |  |  |  |  | 4 |
| Suh 2011^89^ | OSATS (task-specific) | Observational - Non-randomised experimental study | 15 | Novice (Medical students) | Laboratory setting | Expert | Robotic suturing |  | Experienced rater |  | Learning curve: OSATS scores improved with training***.  Concurrent: correlated with improved kinematics |  | 3 |
| Menhadji 2013^98^ | OSATS (task-specific) | Observational - Non-randomised experimental study | 39 | Residents (PGY 2-5) | Laboratory setting | Trained instructor | 5 dry model tasks (rings on a peg, thread the loops, cut the line, needle driving, suturing/knot tying) |  | Trained rater |  | Construct* |  | 2b |
| Egi 2013^90^ | OSATS (GRS & task-specific) | Observational - Non-randomised experimental study | 12 | Intermediate; Expert | Laboratory setting | Expert | Bench model suturing |  | Blinded expert raters | High inter-rater reliability ^Cα^  OSATS GRS 0.891  OSATS checklist 0.803 | Concurrent: Moderate correlation OSATS suturing score to dV-T suture sponge^Sp^ |  | 2b |
| Sobel 2016^62^ | OSATS (GRS & task-specific) | Observational - Non-randomised experimental study | 16 | PGY 2- 6 with no previous standardised robotic training, 4 expert robotic surgeons (who currently perform TORS) | Laboratory setting | Expert | Ex-vivo porcine model TORS. 2 tasks: robotic radical tonsillectomy and base of tongue (BOT) resection |  | Blinded expert rater |  | Construct**pre- curriculum  Comparable scores post-curriculum between trainees and experts | Composite pass score developed based on expert metrics | 2b |
| Willems 2016^88^ | OSATS (GRS and task-specific) | Observational - Non-randomised experimental study | 2 | No experience in microsurgery/robotics | Laboratory setting | Independent rater | Simulation microvascular anastomosis (classic microsurgery vs robotic) |  | Independent rater  Powered study |  | Manual outperformed robotic anastomosis* |  | 3 |
| Møller 2020^91^ | OSATS (task-specific) | Randomised cross-over study | 22 | 1 intern, 12 residents, 7 fellows, 2 attendings. None had robotic experience | Laboratory setting (Operative theatre doing lab tasks) | Expert | 3D Conventional laparoscopy vs robotic suturing dry skin models |  | Blinded expert raters |  | Failed to show construct validity |  | 3 |
| Willuth 2022^63^ | OSATS (GRS & task-specific) | Randomised controlled trial | 40 | Novice (medical students) | Laboratory setting | Expert | Ex vivo porcine cholecystectomy (Lap and robotic) |  | Randomised, powered study Blinded raters |  | Robotic total OSATS scores, GRS and task specific OSATS scores higher in robotic than lap* |  | 2a |
| Ghani 2016^64^ | Prostatectomy Assessment and Competency Evaluation  (PACE) | Observational - Non-randomised experimental study | 10 | Surgeons providing videos | Operating theatre | Crowd sourced: 23 peer surgeons -328 ratings, 784 Amazon Mechanical Turks- 2715 ratings | RARP |  | Blinded peer and crowd sourced raters | Inter-rater reliability i.e. correlation between peer and crowd groups (r=0.75) |  |  | 4 |
| Hussein 2017^66^ | Prostatectomy Assessment and Competency Evaluation  (PACE) | Other: Delphi process | 56 | 28 trainees, 28 attending surgeons | Operating theatre; Other: Delphi consensus | 12 Expert surgeons for Delphi, expert raters then for videos | RARP | Delphi consensus | Blinded expert raters | Inter-rater reliability- Overall Fair to Excellent ^ICC^ | Construct in all domains*-*** |  | 2b |
| Hogg 2016^86^ | Pancreaticojejunostomy-Specific Variables (PJVs) part of the Pancreaticojejunostomy score card | Observational - Non-randomised experimental study | 133 | Expert | Operating theatre | Expert (International clinical fellows) | Robotic pancreaticojejunostomy during a pancreaticoduodenectomy |  | Blinded expert raters | Strong to very strong Intra-rater reliability ^Sp^ Moderate Inter-rater reliability  OSATS and V.Weak for PJV  ^Sp^ | Predictive validity - Higher PJV/OSATS scores significantly correlated with lower rate POPF  - Lower PJV score significantly associated with increased rate POPF |  | 3 |
| Hussein 2017^79^ | Pelvic Lymphadenectomy Assessment and Completion Evaluation (PLACE) | Other: Delphi process | 11 | Expert | Operating theatre; Other: Delphi consensus | Expert | Pelvic lymph node dissection (PLND) in radical cystectomy for bladder cancer | Content- Delphi process | Blinded expert raters | Low to Moderate Inter-rater reliability ^K^ | Concurrent: LN clearance score strong positive correlation with LN count ^Sp^ |  | 2b |
| Hung 2017^80^ | Proficiency Score | Observational - Non-randomised experimental study | 21 | Robotic trainees - 11 residents, 10 fellows | Operating theatre | Expert (Fellows, MIS faculty and Surgical Techs) | RARP & RAPN | Delphi consensus  Expert opinion based on European Association of Urology Robotic Section and literature | Orientation period trainees and raters. Expert raters | Fair to Excellent inter-rater reliability ^ICC^ | Concurrent validity PS very strong overall association with GEARS ^Sp^ Construct PS*** |  | 2b |
| Beulens 2019^67^ | PRostatectomy video Observation To Evaluate and Score Technical skill (PROTEST) | Other: Delphi consensus | 5 to 18 | Expert | Delphi consensus | Expert | RARP | Delphi  consensus |  |  |  |  | 4 |
| Raza 2015^81^ | Robotic Anastomosis Competence Evaluation  (RACE) | Observational - Non-randomised experimental study | 28 | 10 Novices, 10 Beginners, 8 Experts | Laboratory setting | Expert | UVA | Delphi consensus | Blinded expert raters | Good inter-rater reliability ^ICC^ and test-retest (intra-rater) reliability ^ICC^ | Construct*  Concurrent - Strong correlation with GEARS^Sp^ |  | 2b |
| Hoogenes 2018^83^ | Robotic Anastomosis Competence Evaluation  (RACE) | Randomised controlled trial | 39 | 23 Junior trainees (medical students and junior residents PGY 1-2), 16 Experienced trainees (senior residents PGY 3-5 and fellows) | Laboratory setting | Expert | UVA 3D printed model |  | Blinded expert raters. Randomised, powered study | RACE & GEARS Excellent inter-rater reliability  ^ICC^ | dVSS intervention group had higher RACE scores* |  | 1b |
| Khan 2019^78^ | Robotic Anastomosis Competence Evaluation  (RACE) | Observational - Non-randomised experimental study | 6 | Fellowship trainees | Operating theatre | Expert: 1 fellowship trained urologist | UVA |  | Expert rater |  | RACE scores improved over time**  Suture speed associated with RACE score*  No significant association with urinary continence i.e. no predictive validity |  | 3 |
| Witthaus 2020^84^ | Robotic Anastomosis Competence Evaluation  (RACE) | Observational - Non-randomised experimental study | 14 | 9 Novices (caseload <50), 5 experts (caseload >500) | Laboratory setting | Expert; Other: CRPMS | Nerve Sparing RARP 3D printed hydrogel model |  | Blinded expert raters |  | Construct** both RACE & GEARS  Concurrent: Strong inverse correlation RACE scores UVA leak rate^Sp^ |  | 2b |
| Ghani 2016^50^ | Robotic Anastomosis Competence Evaluation  (RACE) | Observational - Non-randomised experimental study | 12 | Expert (MUSIC surgeons) | Operating theatre | Expert (4 peer reviewers per video from 25 surgeons); Crowd sourced (Amazon Mechanical Turk from 30-55 reviewers) | UVA |  | Blinded raters | Moderate and poor inter-rater reliability amongst peer reviewers for RACE and GEARS respectively ^Kα^   Strong positive inter-rater reliability peers and crowd sourced RACE and GEARS respectively ^P^ |  |  | 2b |
| Haque 2022^208^ | RACE &  End-To-End Assessment of Suturing Expertise (EASE) | Observational - Non-randomised experimental study | 6 | 8 training videos, then 39 test videos Experts (100+ robotic cases) and trainee (<100 cases) | Delphi consensus and Operating theatre | 5 Expert Surgeons and 1 educational psychologist | UVA | Delphi consensus with content validity index employed for agreement | Blinded, independent, trained raters  Alignment sessions for reliability | Moderately high inter-rater reliability of EASE^ICC and prevalence-adjusted bias-adjusted Kappa^ | Construct of EASE*-***  Concurrent: Strong correlation RACE and EASE^Sp^ |  | 2b |
| Lovegrove 2017^68^ | Robotic-Assisted Partial Nephrectomy (RAPN) Training Tool | Observational - Non-randomised experimental study | 13 | Expert surgeons | Other: Healthcare Failure Mode and Effect Analysis (HFMEA) | Expert | Robotic-Assisted Partial Nephrectomy (RAPN) | Content-Yes through Healthcare Failure Mode and Effect Analysis (HMFEA) |  |  |  |  | 4 |
| Chow 2021^71^ | Robotic-Assisted Partial Nephrectomy- Specific Scoring System | Observational - Non-randomised experimental study | 12 | Novice (residents) | Laboratory setting | Expert (Five blinded fellowship trained robotic surgery faculty members) | Ex vivo porcine simulation RAPN |  | Blinded expert raters |  | Learning curve: Mean RAPN Specific Scoring System improved over the sessions* |  | 3 |
| Davis 2010^72^ | RARP 11 steps A+ to C rating | Observational - Non-randomised experimental study | 5 | 4 fellows, 1 staff surgeon- 178 cases | Operating theatre | Expert | RARP |  |  |  |  |  | 4 |
| Lovegrove 2016^70^ | RARP Assessment Score | Observational - Non-randomised experimental study | 15 | Other: Robotic surgery trainees | Operating theatre | Expert | RARP | Consensus using Healthcare Failure Mode and Effect Analysis (HMFEA) | Expert mentors Blinded expert raters | No to Poor Inter-rater reliability ^CK^ | Construct- not demonstrated | Learning curve plateaus defined for 5/17 steps of RARP | 3 |
| Lovegrove 2017^69^ | RARP Assessment Score | Observational - Non-randomised experimental study | 15 | Other: Fellows | Operating theatre | Expert | RARP |  |  |  |  |  | 4 |
| Volpe 2015^73^ | RARP procedure-specific scoring scale including Generic Dedicated Scoring Criterion | Observational - Non-randomised experimental study | 10 | Expert | Operating theatre | Expert | RARP | RARP procedure-specific scoring tool developed by expert panel | Blinded expert raters |  | Construct- significantly (no p value provided) | Mean score of 10 or over = pass (safe) | 3 |
| Frederick 2017^65^ | Robotic Hysterectomy Assessment Score (RHAS) | Other: Canadian Task Force classification III (retrospective cohort) | 5 for the expert panel 52 videos | Videos: 7 Novice, 20 advance, 25 Expert. Delphi: 5 Experts | Operating theatre; Other: Delphi method evaluation | Expert | Robotic Hysterectomy | Delphi consensus | Experts | Good inter-rater reliability across 5 out of 6 domains ^ICC^ except for colpotomy | Construct validity** |  | 2b |
| Vaccaro 2013^92^ | Robotic OSATs  (rOSATS) | Randomised controlled trial | 18 | Residents | Laboratory setting | Expert | Ex vivo chicken model dissection and suturing |  | Blinded expert raters Randomised Powered study |  | No significant difference between intervention (simulator training) and control group |  | 3 |
| Iqbal 2022^74^ | Scoring for Partial Nephrectomy  (SPaN) | Other: Delphi process | 10 | Novice; Expert; Other: Trainees and attending surgeons | Other: Delphi process | Expert | Robotic-assisted partial nephrectomy | Delphi consensus | Blinded expert raters | Inter-rater reliability over 75% all domains (linearly weighted percent agreement on 15 videos) | No construct validity- Higher, non-significant scores in expert group |  | 2b |
| Tou 2020^75^ | Task-Performance Metrics: RA-LAR | Other: Delphi process | 18 | 3 surgeons and a behavioral scientist created metrics, 18 experts involved in consensus. | Other: Delphi | Expert; Other: 18 | Robotic anterior resection | Delphi process |  |  |  |  | 4 |
| Gómez Ruiz 2022^77^ | Task-Performance Metrics: RA-LAR | Observational - Non-randomised experimental study | 14 | Colorectal surgeons from ESCP working group- 5 Novice (<30 RA-LAR procedures), 9 Experienced (30+ RA-LAR procedures) | Operating theatre | Expert (2 consultant colorectal surgeons >50 robotic resections) | Robotic anterior resection |  | Blinded, independent, expert raters  Trained to use both tools until using consistently with IRR >0.8 | IRR (agreement/agreement+disagreements)  Task performance metrics IRR 0.94 and GEARS IRR 0.49, | Higher performing experienced (below the median) surgeons performed more procedural steps (not significant)  Construct Task performance metrics* -** with better discrimination (58-64%) than GEARS (15-41%) of higher performers.  Higher performing experienced surgeons had fewer critical* and total errors* than novice group Higher performing experienced surgeons outperformed novices in critical errors** and total errors** at critical step of rectal dissection/transection "Moderately strongly" negative correlation of operative experience and total errors (r=-0.517, p=0.028) |  | 2b |
| Mottrie 2021^76^ | Task-Performance Metrics: RARP | Other: Delphi consensus & Observational study | 19 | Expert | Operating theatre; Other: Delphi consensus | Expert | RARP- Operative video review of 12 expert (>500 RARPS) and 12 novice (<10 RARPS) videos | Face & Content - Delphi Consensus agreement | Expert robotic surgeons were trained to use the tool until inter-rater reliability >0.8, blindly scored RARP full case videos (12 very experienced and 12 novice videos) | High inter-rater reliability 0.85 (IRR calculated by number of agreements/no. of agreements + disagreements) | Construct*-** |  | 2b |
| Puliatti 2021^93^ | Task-Performance Metrics: Robotic Suturing and Knot Tying | Other: Delphi consensus & Observational study | 13 | Delphi: 13 experts. Wet ex-vivo lab: 10 experienced, 9 novice urology surgeons | Laboratory setting; Other: Delphi consensus | Expert | Ex-vivo chicken anastomosis model | Delphi consensus | Blinded expert rating Trained until inter-rater reliability >0.8 | Strong inter-rater reliability( IRR=agreements/agreements+disagreements) | Construct*** | Anastomotic leakage assessed, not evaluation of the tool | 2b |
| Puliatti 2021^97^ | Task-Performance Metrics: Robotic Vessel Dissection/Coagulation/Clipping | Other: Delphi consensus & Observational study | 4 to 16 | Robotic surgeon experts and behavioural scientist. | Laboratory setting; Other: Delphi consensus | Expert | Ex-vivo chicken model | Delphi consensus | Blinded expert rating Trained until inter-rater reliability >0.8 | Strong inter-rater reliability ^P^ | Construct** |  | 2b |
| Chang 2003^94^ | Task-specific checklist | Observational - Non-randomised experimental study | 8 | Attending surgeons- no clinical robotic experience | Laboratory setting | Surgeon observers | Intracorporeal knot tying |  | Blinded raters | Strong inter-rater reliability (stated as over 0.8 UTD test) | Robot knot tying time and scores significantly surpassed laparoscopy* |  | 3 |
| Singh 2018^95^ | Task-specific checklist: "Task Progression Score" | Observational - Non-randomised experimental study | 8 | 1 consultant, 7 Higher surgical trainees | Laboratory setting | Expert | Suturing robotic vs laparoscopic |  |  |  | Technical performance superior in robotic suturing with better progression* and error scores* when under time pressure, but not when self-paced  Robotic suturing no difference in leak rate |  | 3 |
| Guni 2018^161^ | Task-specific checklist: Robotic suturing | Observational - Non-randomised experimental study | 28 - 39 | 22 Novices (no operative experience), 13 Experts (>50 RARPS) videos needle driving. 18 and 10 respectively participated for knot tying, 39 novices final checklist validation | Laboratory setting | Expert | UVA synthetic dry lab model | 2. Content- Created by expert panel | Independent expert rater | 2. Acceptable to good intra-rater reliability (Cronbach's alpha 0.752 knot tying & 0.887 needle driving) | Construct validity**  Concurrent validity: Strong correlation needle driving and GEARS^Sp^ No significant correlation of GEARS with Knot tying  Strong to very strong correlation GEARS and checklist overall scores by expert observer for both sets of novice videos^Sp^ |  | 2b |
| Chowriappa 2015^82^ | UVA evaluation score | Randomised controlled trial | 52 | Novices (all residents or fellows <25 hours of robotic experience) | Laboratory setting | Expert | UVA | Weak face validity - Created by one expert surgeon | Randomised, powered study Expert raters | Inter-rater reliability GEARS 0.72 UVA 0.68 (UTD statistical test) | Concurrent validity with GEARS.  Construct*-** intervention group outperformed control. |  | 2a |

| Table S5: Error-Based Tools | | | | | | | | | | | | |  |
| --- | --- | --- | --- | --- | --- | --- | --- | --- | --- | --- | --- | --- | --- |
| Study | Ax Tools Compared | Study type | Participant  No. | Expertise level | Setting | Raters | Task | Test  Content | Response process | Internal Structure | Relationship other variables | Consequence | L  O  E |
| Korets 2011^193^ | Arbitrary time penalties for named errors | Observational - Non-randomised experimental study | 16 | Residents: 10 novice (<50 robotic cases), 6 intermediate (50+) | Laboratory setting | N/A | Dry model- Ring rail and knot tying |  |  |  |  |  | 4 |
| Cho 2013^237^ | DV index score including number of task-specific errors to calculate accuracy (defined task-specific errors in needle control and suturing/knot tying) | Randomised controlled trial | 11 | Expert (Certified General Surgeons, no robotic experience) | Laboratory setting | N/A | da Vinci dry lab exercises pre and post curriculum test |  | Randomised groups |  | Construct*- yes significantly better DV index scores in the VR intervention group (p=0.028) | DV index competency score = 10/time x accuracy (errors used to help compute accuracy) Although no defined pass/fail benchmark | 2a |
| Abdelaal 2020^286^ | Error time = sum of all times ring or surgical tool touches the rail | Randomised controlled trial | 12 | Novice | Laboratory setting |  | Ring rail task dry model |  |  |  |  |  | 4 |
| Hutchinson 2022^190^ | Executional and Procedural Errors in tasks and gestures rubric (based on Human Reliability Analysis) | Observational - Non-randomised experimental study | 8 (JIGSAWS) | Self-proclaimed Expert (>100 hours robotic experience), Intermediate (10-100), Novice (<10 hours) or defined by OSATS scores | Laboratory setting | Expert | JIGSAWS data set: - Suturing - Needle Passing |  |  |  | Construct validity of executional error frequencies in suturing Concurrent validity-  Highly significant negative correlation between OSATS and procedural error frequency (r= -0.51, p<0.001 r= -0.51, p<0.001, UTD statistical test used) in suturing. No correlation for needle passing Highly significant (p<0.001) positive correlation of executional errors and time (r=0.837) in suturing No significant correlation or construct validity with needle passing |  | 3 |
| Arain 2012^210^ | FLS scoring system (modified) | Observational - Non-randomised experimental study | 53 | 47 resident, 3 fellows, 5 attendings (2 drop out) | Laboratory setting |  | 9 dry model tasks (5 from FLS curriculum) |  |  | Excellent inter-rater reliability ^ICC^ Excellent test-retest reliability ^Cα^ and Good internal consistency ^Cα^ |  | Expert derived performance levels All trainees reached proficiency | 3 |
| Dulan 2012^211^ | FLS scoring system (modified) | Observational - Non-randomised experimental study | 12 | 4 Novices (medical students), 8 Expert- (surgeons performed 15-600 robotic cases) | Laboratory setting | Expert | 9 dry model tasks (5 from FLS curriculum) |  |  |  | Construct*** | Expert data used to define proficiency levels per task | 2b |
| Dulan 2012^212^ | FLS scoring system (modified) | Observational - Non-randomised experimental study | 2 | Novice; Expert | Laboratory setting | Not specified | 9 dry model tasks (5 from FLS curriculum) |  |  |  | Construct*** | Pass/Failed proficiency defined (mean expert ratings - 2SD) | 3 |
| Bric 2014^213^ | FLS scoring system | Observational - Non-randomised experimental study | 17 | 14 Novices data assessed out of 24 (medical students), 3 experienced robotic surgeons (FLS tasks only) | Laboratory setting | Not specified | FLS tasks - pegboard transfer and intracorporeal knot tying |  |  |  | Construct*** for knot tying |  | 2b |
| Aghazadeh 2016^146^ | FLS scoring system | Observational - Non-randomised experimental study | 21 | 17 Trainees (urology residents, fellows), 4 Experts (attending physicians) | Operating theatre; Laboratory setting | Expert | Fundamental inanimate robotic skills tasks [FIRST] |  |  |  | Predictive of console performance in the OR: Very strong correlation with intra-operative GEARS score ^Sp^ |  | 2b |
| Suh 2016^214^ | FLS scoring system | Observational - Non-randomised experimental study | 15 | Other: 12 medical students, 3 residents | Laboratory setting | Experienced medical fellow | Robotic suturing whilst being distracted to different levels |  |  |  | Distraction leads to measurement of worse performance in robotic suturing using objective measures EMG/Kinematic and errors and subjective NASA-TLX score*-*** |  | 3 |
| Valdis 2016^169^ | FLS scoring system | Randomised controlled trial | 40 | Other: Surgical trainees | Laboratory setting | Expert | Ex vivo-porcine harvest of internal thoracic artery and mitral valve annuloplasty |  | Blinded rater Randomised, powered study |  | Construct ** expert vs control. Intervention group reached expert defined proficiency level | Proficiency level defined by experts (score=max time-expert pooled time-errors | 2a |
| Hutchins 2018^209^ | FLS scoring system | Observational - Non-randomised experimental study | 16 | 10 residents, 6 fellows/faculty | Laboratory setting |  | 2 FLS tasks - Peg transfer  - Precision cutting |  | Randomised and blinded assignment of participants |  | Learning curve demonstrated both groups improved with additional attempts  No Construct: non-significant higher scores with more experienced group |  | 2b |
| Singh 2018^95^ | FLS Scoring system (modified) | Observational - Non-randomised experimental study | 8 | 1 consultant, 7 Higher surgical trainees | Laboratory setting | Expert | Intracorporeal suture robotic vs laparoscopic |  |  |  | Technical performance superior in robotic suturing with better progression* and error scores* under time pressure | Leak and knot tensile strength | 3 |
| Alshuaibi 2019^247^ | FRS general and task-specific metrics (overall score =100−M×errors in major metrics*−m/10×errors in minor metrics) | Observational - Non-randomised experimental study | 37 | 8 Experts (>50 robotic cases), 11 Intermediates (5-50), 18 Beginners (<5) | Laboratory setting | Expert | FRS dome simulation and dry |  | Expert rater |  | Concurrent validity: No correlation to Very Strong between RXM simulator score and dry model equivalent ^P^ |  | 2b |
| Hung 2013^163^ | Fundamental Inanimate Robotic Skills Tasks (FIRST) composite score: efficiency/precision + time to complete - error penalties = overall score | Observational - Non-randomised experimental study | 49 | 38 Novice/trainees (<30 cases), 11 experts (30+ robotic cases) | Laboratory setting | Expert | 1. FIRST (Horizontal mattress, Pattern Cut, Dome & Peg transfer, Needle driving) | Face previously in Goh study but no text for it, supplement section of journal |  |  | Construct***  Concurrent validity: Strong negative correlation with VR ^Sp^  Very strong negative correlation with GEARS ^Sp^ |  | 2b |
| Altok 2018^287^ | Generic and RARP-specific errors: Likert scale 0-5 How easy or difficult a mistake is to correct | Observational - Non-randomised experimental study | 100 | 6 staff, 94 trainees | Operating theatre | Expert | RARP |  |  |  |  |  | 4 |
| Volpe 2015^73^ | Generic Dedicated Scoring Criterion (GDSC) | Observational - Non-randomised experimental study | 10 | Expert; Other: Fellows | Operating theatre | Expert |  | Face/content- developed by expert panel |  |  | Construct- “significant” (no p value) | 10 out of 16 or more considered safe | 3 |
| Goldenberg 2017^162^ | Generic Error Rating Tool (GERT) | Observational - Non-randomised experimental study | 1 | 1 Surgeon | Operating theatre | Expert | RARP |  | Blinded expert rater |  | Concurrent: GERT strong inverse correlation to GEARS ^Sp^  Strong correlation to total adverse events ^Sp^  No predictive validity with urinary continence |  | 3 |
| Chen 2021^192^ | Generic Error Rating Tool (GERT) | Other: Prospective single armed study non-randomised, compared to RCT of laparoscopy | 144 (48 Robotic total gastrectomy, 96 laparoscopic total gastrectomy) | 2 Experts >50 RTG and LTG experience. | Operating theatre | Expert | Robotic vs Laparoscopic Total Gasterectomy |  | Blinded, trained, expert rater |  | RTG vs LTG lower number of average errors*** No predictive validity  Moderate correlation between errors and task load scores (r=0.51, p<0.001). Spearmans or Pearsons- UTD which |  | 2b |
| Nisky 2014^264^ | Kinematics endpoint error | Observational - Non-randomised experimental study | 16 | 10 Novice nonmedical users no da Vinci; 6 Expert (experienced robotic surgeons) | Laboratory setting | Automated |  |  |  |  | Construct*** |  | 2b |
| Chandra 2010^250^ | ProMIS(TM) hybrid surgical simulator Task-specific discrete errors technique/accuracy/knot placement and tightness | Observational - Non-randomised experimental study | 29 | 20 Novices (<50 laparoscopic hours), 9 Substantial lap/robotic experience (50+) | Laboratory setting | Expert | Intracorporeal suturing |  | Blinded expert raters Powered study | Good internal consistency between metrics of ProMIS ^Cα^ | Robotics outperformed laparoscopic attempts in novice group*** |  | 2b |
| Davis 2010^72^ | RARP 11 steps rating A* to C - minor and major procedure-specific corrections | Observational - Non-randomised experimental study | 5 | 4 fellows, 1 staff surgeon- 178 cases | Operating theatre | Expert | RARP |  |  |  |  |  | 4 |
| Tou 2020^75^ | Task-Performance Metrics for RA-LAR- errors and critical errors | Other: Delphi process | 18 | 3 surgeons and behavioural scientist created metrics  18 experts involved in consensus. | Other: Delphi | Expert | Anterior resection | Yes |  |  |  |  | 4 |
| Gómez Ruiz 2022^77^ | Task-Performance Metrics for RA-LAR- errors and critical errors | Observational - Non-randomised experimental study | 14 | Colorectal surgeons from ESCP working group- 5 Novice (<30 RA-LAR procedures), 9 Experienced (30+ RA-LAR procedures) | Operating theatre | Expert 2 consultant colorectal surgeons >50 robotic resections | Robotic anterior resection |  | Blinded expert raters Trained until inter-rater reliability >0.8 | Inter-rater reliability 0.94 (IRR calculated by number of agreements/no. of agreements + disagreements)  GEARS IRR 0.49, | Construct*-  Higher performing experienced surgeons had fewer critical* and total errors* than novice group Higher performing experienced surgeons outperformed novices in critical errors** and total errors** at critical step of rectal dissection/transection  Moderate to strong negative correlation of operative experience and total errors (r=-0.517, p=0.028) |  | 2b |
| Mottrie 2021^76^ | Task-Performance Metrics for RARP- errors and critical errors | Other: Delphi consensus & Observational study | 19 to 24 | 19 Experts in consensus. Study 2 - 12 very experienced surgeons (>500 RARPs), 12 novice surgeons | Operating theatre; Other: Delphi consensus with Operative video review of 12 expert (>500 RARPS) and 12 novice (<10 RARPS) videos | Expert | RARP | Delphi consensus agreement | Blinded expert raters Trained until inter-rater reliability >0.8 | Inter-rater reliability 0.85 (IRR calculated by number of agreements/no. of agreements + disagreements) | Construct- Significant difference between "very experienced surgeons (VES)" group and novice urology surgeons, performing ~4% more steps and 72% fewer errors*  5x more errors between top performing and bottom performing very experienced group** |  | 2b |
| Puliatti 2021^97^ | Task-Performance Metrics for Robotic Vessel Dissection/Coagulation/Clipping -errors and critical errors | Observational - Non-randomised experimental study | 4 -16 at different stages | Novice; Expert; Other: Robotic surgeon experts and behavioural scientist. | Laboratory setting; Other: Delphi consensus and task completion | Expert | Ex vivo chicken model | Yes- Delphi consensus | Blinded expert raters Trained until inter-rater reliability >0.8 | Strong inter-rater reliability 0.96  (IRR calculated by number of agreements/no. of agreements + disagreements) | Construct** Experts 155% fewer performance errors  Errors positively correlated with time taken in novice group (r=0.619, p=0.014) |  | 2b |
| Puliatti 2021^93^ | Task-Performance Metrics of Robotic Suturing and Knot Tying skills- errors and critical errors | Other: Delphi consensus and observational study | 13 | Novice; Expert; Other: Delphi: 13 experts. Wet ex-vivo lab: 10 experienced, 9 novice urology surgeons | Laboratory setting; Other: Delphi consensus | Expert | Ex-vivo chicken anastomosis model | Delphi consensus | Blinded expert raters Trained until inter-rater reliability >0.8 | Strong inter-rater reliability 0.92  (IRR calculated by number of agreements/no. of agreements + disagreements) | Construct*** - 74% more errors by novices | Anastomotic leakage tested | 2b |
| Walker 2017^263^ | Task-specific error checklist | Observational - Non-randomised experimental study | 8 | Other: 7 PGY 2-5 and 1 first year fellow |  | Expert | Female pelvis simulation model- Bleeding simulation |  | Expert raters |  |  |  | 4 |
| Rice 2019^186^ | Task-specific error checklist: 3 errors- damaged material, broken suture, air knot. | Observational - Non-randomised experimental study | 36 | 6 residents, 19 fellows and 11 attendings | Laboratory setting | 2 Expert ratings, The Crowd- premedical students | 3 dry model anastomosis drills  performed in Whipple's (HJ/GJ/PJ) |  |  | Good to excellent inter-rater reliability of Total Errors ^ICC^ |  |  | 3 |
| Chang 2003^94^ | Task-specific error checklist: Error score calculator- step checklist with defined errors -developed through review of 30 archived recordings | Observational - Non-randomised experimental study | 8 | Attending surgeons- no clinical robotic experience | Laboratory setting | Surgeon observers | Intracorporeal knot tying |  | Examiner blinded | Inter-rater reliability 0.8 (unknown statistical test) | Strong correlation error scores to longer completion times^P^ |  | 3 |
| Stegemann 2013^12^ | Task-specific errors | Randomised controlled trial | 53 | 9 medical students, 26 residents, 10 fellows, 8 practicing surgeons. All with no robotic experience | Laboratory setting | Expert | 3 dry tasks (Ball placement, suture pass, fourth arm manipulation) |  | Blinded trained raters Randomised, powered study | Inter-rater reliability 0.7 (unknown statistical test) | Construct* Intervention group who undertook FSRS VR curriculum outperformed control group |  | 2a |
| Foell 2013^219^ | Task-specific errors | Observational - Non-randomised experimental study | 37 | Novices: 7 Junior and 12 senior resident, 15 fellows, 3 staff surgeons | Laboratory setting | N/A; Other: Dry model not specified otherwise on dVSS | 2 dry models (ring transfer and needle passing) |  |  |  | Improved mean errors pre- to post course** |  | 2b |
| Tarr 2014^183^ | Task-specific errors | Observational - Non-randomised experimental study | 165 pre test, 99 post test | Residents | Laboratory setting | Expert | ROSATS curriculum 4 dry tasks |  |  |  | Participants' errors reduced**  Weak negative correlation with OSATS^P^ (no p value) |  | 2b |
| Harris 2017^288^ | Task-specific errors | Observational - Non-randomised experimental study | 120 | Medical students | Laboratory setting |  | Dry lab tasks- ring wire, knot tying |  | Powered study |  | Moderate correlation of errors to jerk (technically and APM calculated) (r=0.52, p<0.001) |  | 3 |
| Knab 2017^184^ | Task-specific errors | Observational - Non-randomised experimental study | UTD | Surgical fellows | Operating theatre; Laboratory setting | Expert | Dry model suturing and specialty-specific anastomoses |  |  |  | Reduced errors with practice*** | 3 experts used to obtain average "gold standard" metrics | 3 |
| Tam 2017^185^ | Task-specific errors | Observational - Non-randomised experimental study | 15 | Surgical oncology fellows | Laboratory setting | Expert | Dry biotissue models (pancreatico-, gastro-, hepaticojejunostomy |  | 2 Blinded expert raters | Strong inter-rater reliability^Sp^ | Construct* | Proficiency benchmarked by 3 experts | 2b |
| Willuth 2022^63^ | Task-specific errors: intra-op complications | Other: Randomised crossover study | 40 | Medical students | Laboratory setting | Expert | Ex-vivo porcine cholecystectomy (Lap and robotic) |  | Unclear if blinded rating |  | Robotic less complications ** |  | 3 |
| Elhage 2015^289^ | Task-specific errors: Number and mode | Observational - Non-randomised experimental study | 6 | 5 consultants, 1 senior fellow (<2 to > 5 years Robotic experience, 2 frequent and 4 infrequent users) | Laboratory setting | Expert | UVA model (Dacron vascular grafts) - previously validated |  | Blinded expert - video review |  | Moderate correlation number of errors and time to complete robotic anastomosis ^Sp^ Lap highest errors**  Construct validity- most experienced Lap surgery committed no greater number of errors for this approach than others.  Open - less mean errors 0.3 compared to robotic 0.9** |  | 3 |
| Satava 2020^15^ | Task-specific errors: Number of errors | Randomised controlled trial | 99 | Residents, fellows, attendings | Laboratory setting | Expert | 5 robotic taks: -knot tying -suturing -4th arm cutting -Puzzle piece dissection -vessel energy |  | Blinded raters |  | After training, performance improved for task errors between 47.4% and 55.3%. Nevertheless there was no significant difference between groups at post-test for error.  All groups significantly improved to post-test, no significant difference between groups in error or time |  | 3 |
| Ahmad 2021^194^ | Task-specific errors: Number of errors | Observational - Non-randomised experimental study | 30 | Senior Fellows - Experience ranked none to high | Laboratory setting | Not specified | 19 biotissue drills | Yes | Blinded raters |  | Concurrent validity: Pre-test error scores in one task inversely correlated to OSATS* |  | 3 |
| Lendvay 2013^238^ | Task-specific errors: Number of errors ("technical and cognitive") | Randomised controlled trial | 51 | PGY 1-6, surgical fellows, faculty | Laboratory setting | Not specified | 2 dry model tasks from FLS curriculum |  |  |  | Significant reduction in errors in suturing after one warm up session* |  | 3 |
| Guru 2015^290^ | Tool-based metrics | Observational - Non-randomised experimental study | 10 | 2 Beginners ,5 Competent and Proficient, Expert 3 | Laboratory setting | Not specified | 5 Dry lab tasks |  |  |  | Construct** |  | 2b |
| Chowriappa 2015^82^ | UVA evaluation tool - Critical errors | Randomised controlled trial | Expert | Novices (all residents or fellows <25 hours of robotic experience) | 52 | Expert | UVA | Weak face validity - Created by one expert surgeon | Randomised, powered study Expert raters | Inter-rater reliability (unable to determine statistical test) GEARS 0.72 UVA 0.68 | Concurrent validity with GEARS. Construct*-** intervention group outperformed control. |  | 2a |

| Table S6: Simulator Automated Performance Metrics | | | | | | | | | | | | |
| --- | --- | --- | --- | --- | --- | --- | --- | --- | --- | --- | --- | --- |
| Study | Simulator platform | Study type | Participant  No. | Expertise level | Setting | Task | Test  Content | Response process | Internal Structure | Relationship other variables | Consequence | L  O  E |
| da Vinci Skills Simulator/da Vinci Surgical Skills Simulator | | | | | | | | | | | | |
| Hung 2011^215^ | dVSS | Observational - Non-randomised experimental study | 63 | 16 Novices (no surgical training), 32 Intermediate (surgical training with <100 robotic cases), 15 Experts (median 315 cases) | Laboratory setting | 10 VR exercises | Face and Content | Powered study Standardised orientation |  | Construct: Experts vs. intermediates and novices*** Intermediates vs. novices** |  | 2b |
| Finnegan 2012^227^ | dVSS | Observational - Non-randomised experimental study | 39 | "Arbitrary boundaries": Group I (0-20 robotic procedures),Group II (21-150), Group III (>150). No participant was a pure surgical novice | Laboratory setting | 24 VR exercises |  |  |  | Construct*** |  | 2b |
| Kelly 2012^216^ | dVSS | Observational - Non-randomised experimental study | 38 | 19 novice (0 robotic cases), 9 Intermediate (1-74 robotic cases), 10 expert (>75 robotic cases) | Laboratory setting | 5 VR tasks | Face and Content |  |  | Construct*-*** between novice and experienced (Intermediate and Expert combined) groups No significant difference between intermediate and expert groups |  | 2b |
| Hung 2012^200^ | dVSS | Randomised controlled trial | 24 | Robotic surgery trainees | Laboratory setting | 10 validated dVSS tasks |  | Randomised, powered study |  | Concurrent: Correlation between overall dVSS metrics and GOALS scores^Sp^  Predictive of console performance in the lab: Correlation between baseline simulator overall scores and final tissue performance^Sp^ but did not show significant difference between VR training (intervention) and control group in the animal tissue performance |  | 1b |
| Alzahrani 2013^217^ | dVSS | Observational - Non-randomised experimental study | 48 | 30 Novices (0 robotic cases), 12 Intermediates (1-74), 6 Experts (75+ cases) | Operating theatre; Laboratory setting | 9 VR tasks | Face and Content |  |  | Construct: Experts & Intermediates vs. Novices*. Experts outperformed intermediates 3/9 tasks* |  | 2b |
| Brinkman 2013^231^ | dVSS | Observational non-randomised experimental study | 17 | Medical students | Laboratory setting | Ring and rail II task |  | Data anonymously analysed |  | Learning curve exhibited* | Pass fail based on proficiency compared to expert score i.e. 90% criterion (9/17 novices reached within 10 repetitions) | 2b |
| Hung 2013^163^ | dVSS | Observational - Non-randomised experimental study | 49 | 38 Novice/trainees (<30 cases), 11 Experts (30+ robotic cases) | Laboratory setting | 4 VR tasks |  |  |  | Construct*** Concurrent: Strong correlation with GEARS and FIRST composite score^Sp^ |  | 2b |
| Vaccaro 2013^92^ | dVSS | Randomised controlled trial | 18 | Residents | Laboratory setting | 9 VR tasks |  | Randomised, powered study |  |  | Pass/Fail- 80% overall and more than 60% in all metrics | 3 |
| Lyons 2013^218^ | dVSS | Observational - Non-randomised experimental study | 43 | 25 novices (10 or less robotic procedures), 8 Intermediates (11-50), 13 Experts (>50) | Laboratory setting | 8 VR tasks | Face and Content | Orientation and familiarisation |  | Construct*-***- Yes between experts and intermediates vs. novices. 10/85 metrics performed well at distinguishing novice and expert No significant difference between intermediates and experts |  | 2b |
| Foell 2013^219^ | dVSS | Observational - Non-randomised experimental study | 37 | Novice; Other: 7 Junior and 12 senior resident, 15 fellows, 3 staff surgeons (all identified as robotic novices) | Laboratory setting | dVSS curriculum | Face | Standardised training |  |  | 80% score before progressing | 2b |
| Ramos 2014^164^ | dVSS | Observational - Non-randomised experimental study | 36 | 24 Novice- no cases as primary surgeon (medical students and surgical interns), 12 Expert - 30+ cases as primary surgeon (attendings and fellows) | Laboratory setting | 3 VR tasks | Face |  |  | Concurrent: - moderate to strong correlation with GEARS^Sp^ |  | 2b |
| Sheth 2014^224^ | dVSS | Observational - Non-randomised experimental study | 18 | Other: 9 Medical students, 22 Gynaecology residents PGY 1-2 (JUNIOR)  PGY 3-6, 3 Fellows (Senior) 34 but after exclusion 18 participants completed: 8 junior and 10 senior | Laboratory setting | 4 VR tasks |  | Orientation to robotic system |  | Construct** |  | 2b |
| Culligan 2014^201^ | dVSS | Observational - Non-randomised experimental study | 23 | 14 surgeon novices (study group), 5 experts to benchmark, 4 controls (no curriculum, novices) | Operating theatre; Laboratory setting | 10 VR tasks | Face | Orientation standardised Experts blinded to other participants scores  Powered study |  | Predictive of console performance in lab and OR/Construct: Expert and study (VR) surgeons outperformed control group (no VR curriculum) in terms of EBL and time ***in da Vinci wet porcine model and live surgery) | Best attempts defined optimal expert performance | 2b |
| Connolly 2014^225^ | dVSS | Observational - Non-randomised experimental study | 24 | Novice; Expert; Other: 20 novices (medical students), 4 credentialed robotic surgeons (>20) | Laboratory setting | 5 VR tasks |  | Same orientation to consoles |  | Construct** |  | 2b |
| Bric 2014^213^ | dVSS | Observational - Non-randomised experimental study | 14 | Medical students (initially 25 but data collection error and 1 dropout), 3 experienced robotic surgeons (FLS tasks only) | Laboratory setting | 5 VR tasks |  |  |  |  | Proficiency targets defined by mean performance of experienced robotic surgeons (time and overall score) | 3 |
| Carter 2015^177^ | dVSS | Randomised controlled trial | 41 | Other: Residents | Laboratory setting | Tubes |  | Randomised, powered study |  | Intervention group who received formative peer reviewed feedback using GEARS had significantly improved dVSS scores and outperformed control group |  | 2a |
| Kiely 2015^160^ | dVSS | Randomised controlled trial | 23 | Other: Residents and attendings | Laboratory setting | Pre- and post-test suture sponge task |  | Randomised. Did not reach sample size |  | Concurrent: Moderate correlation GOALS+ with suture sponge 1^Sp^  Learning curve: intervention (VR curriculum group) improved more than control in Suture sponge 1***, GOALS+*, GEARS* and knot tying* | All component scores >60% and overall score green checkmark, non consecutively, two separate occasions. | 2a |
| Liss 2015^228^ | dVSS | Observational - Non-randomised experimental study | 38 | Urology residents and surgeons. Novices 0 cases, intermediate 1-49, experienced 50-200, expert >200 | Laboratory setting | Tubes |  |  |  | Construct: Experts vs residents and novices*.  MScore 1st attempt moderate correlation with previous no. of robotic cases^Sp^  2nd attempt, very weak (no) correlation | MScoreTM- developed from mean and SD of 100 robotic surgeons (75+ cases) to facilitate credentialing and privileging | 2b |
| Valdis 2015^168^ | dVSS | Randomised controlled trial | 19 | Surgical trainees | Laboratory setting | 9 VR tasks | Content: Yes, assessed by experts | Randomised, powered study |  | Construct: VR intervention group outperformed control at post-test***. VR group comparable to experts after curriculum | 90% or over, with no critical error. Defined by expert performance to define proficiency | 2a |
| Vogell 2015^230^ | dVSS | Observational - Non-randomised experimental study | 8 | Third year residents | Operating theatre; Laboratory setting | VR tasks form Intuitive online module |  |  |  | Predictive of console performance in the OR: Strong correlation between rate of change in OR OSATS scores and total amount of simulation^P^ |  | 3 |
| Volpe 2015^73^ | dVSS | Observational - Non-randomised experimental study | 10 | Expert: Fellows | Operating theatre; Laboratory setting | 4 VR tasks |  |  |  | Learning curve: Scores over training programme improved**-*** |  | 3 |
| Yamany 2015^229^ | dVSS | Observational - Non-randomised experimental study | 13 | 4 senior and 9 junior residents | Laboratory setting | VR suturing/knot tying task |  |  |  | Construct: previous simulator experience and trainee level lower time to complete*. Fatigue associated with increased time*** |  | 2b |
| Aghazadeh 2016^146^ | dVSS | Observational - Non-randomised experimental study | 21 | 17 Trainees (urology residents, fellows), 4 Experts (attending physicians) | Operating theatre; Laboratory setting | 8 VR tasks |  |  |  | Predictive of console performance in the OR: Very Strong correlation of dVSS overall with GEARS overall intra-operative scores^Sp^ |  | 2b |
| Valdis 2016^169^ | dVSS | Randomised controlled trial | 40 | Surgical trainees | Laboratory setting | 9 VR tasks |  | Randomised, powered study |  | Learning curve: Intervention groups, including VR, reached proficiency and end scores no different to experts. | Proficiency defined by experts (90% or over with no critical errors) | 2a |
| Brown 2017^226^ | dVSS | Other: Prospective randomised study | 26 | Novic: Residents with no previous robotic experience | Laboratory setting | Skills test at beginning and end of 4 week practice period on the respective platforms: Group 1 dVSS, Group 2 on da Vinci Si generation dry models. |  |  |  | Concurrent: No difference between VR and dry model practice groups pre and post test scores |  | 2b |
| Mills 2017^181^ | dVSS | Observational - Non-randomised experimental study | 10 | Expert: Attending robotic surgeons | Operating theatre; Laboratory setting | 4 VR tasks |  |  |  | Construct: weak/moderate correlation of average simulator score to number of previous robotic operations^Sp^  No Predictive: No correlation of dVSS score with intraoperative GEARS score |  | 3 |
| Newcomb 2018^204^ | dVSS | Other: Prospective methods comparison study | 30 | Other: 19 residents, 7 fellows, 4 faculty | Laboratory setting | 5 VR tasks | Face and content |  |  | Construct** but no significant difference between fellows and faculty.  Concurrent: VR and Dry lab model equivalent showed strong correlation with overall score of VR and R-OSATS^Sp^ | Defined minimal competency scores for each task | 2b |
| Robison 2018^232^ | dVSS | Observational - Non-randomised experimental study | 15 | Other: PGY2-5 residents | Laboratory setting | 5 VR tasks |  |  |  | No significant difference in MScore when pre on-call and fatigued post on-call. | MScoreTM 90th centile pass mark | 2b |
| Havemann 2019^220^ | dVSS | Observational - Non-randomised experimental study | 32 | 1 novice (0 procedures), 11 intermediate (1-50 procedures), 10 experienced (>50). 28 completed all, 4 only 1st attempt | Laboratory setting | 5 VR tasks | Content |  | Excellent reliability 9th and 10th attempt average scores^ICC^ | Construct*** between groups. Intermediate vs novice***, expert vs. novice**. No difference intermediate vs. expert. Moderate correlation between 1st average score and expertise level^Sp^  Subjective Mental Effort Questionnaire (SMEQ)- reduced from 1st to 10th attempt | Pass/fail - average of expert group minus 1 SD (75%) | 2b |
| Gerull 2019^206^ | dVSS | Observational - Non-randomised experimental study | 31 | Novice: Residents novice to robotics | Operating theatre; Laboratory setting | VR tasks |  |  |  | Learning curve: after training for 2.3 hours on average, RO-SCORES improved |  | 2b |
| Almarzouq 2020^166^ | dVSS | Randomised controlled trial | 14 | Novice (robotic naïve residents) | Operating theatre; Laboratory setting | RARP (bladder mobilization (all), UV anastomosis (senior residents)) compared with nine dVSSS tasks | Construct validity from previous research guided choice of VR tasks | Randomised, powered study  Blinded crowd raters |  | Predictive of console performance in the OR: "energy & dissection" task on dVSS correlated strongly with GEARS efficiency during bladder mobilisation ^Sp^ . Moderate to Strong correlation force sensitivity in two VR tasks correlate with the same domain in bladder mobilization^Sp^. Very Strong correlation Total GEARS for two VR tasks with total GEARS for UVA ^Sp^ | Established competency cut-offs for 9 dVSS tasks (norm reference method)= mean scores of 5 experts minus 1 standard deviation | 2a |
| Ahmad 2021^194^ | dVSS | Observational - Non-randomised experimental study | 30 | Senior Fellows - Experience ranked none to high | Laboratory setting | 23 module VR curriculum |  |  |  | Learning curve: Pre-test and post-test outcomes from VR curriculum: VR scores improved, errors reduced, time reduced and OSATS scores improved  Predictive of console performance in the lab: Strong correlation of Time spent on VR with % mastery^P^ | Proficiency at >90% in modules Compliance to curriculum used to create quartiles | 3 |
| Kelly 2021^159^ | dVSS & dVLogger | Randomised controlled trial | 34 to 41 | Surgeons: Junior, Senior and Attending | Operating theatre; Laboratory setting | 1. 5 groups different warm up curricula, either one of or all 4 tasks dVSS  2. Operating room surgery - 434 recorded, GEARS ratings of 45 videos 347 with kinematics included |  | Randomised, no sample size calculation |  | Warm up- no significant difference between treatment arms and kinematics  Kinematics and events metrics not significantly associated with GEARS score |  | 2a |
| Gleason 2022^233^ | dVSS | Observational - Non-randomised experimental study | 21 out of 53 completed the curriculum | Trainees | Laboratory setting | 24 exercises developing a multidisciplinary robotic VR curriculum |  |  |  | Learning curve improvement pre to post-test** | Scores>90 on individual exercises and the post-test were required for successful completion | 2b |
| Combination: da Vinci Skills Simulator, dV-Trainer, RobotiX Mentor | | | | | | | | | | | | |
| Perrenot 2012^221^ | dVSS & dV-T | Observational - Non-randomised experimental study | 75 | Groups 1-5: 1 (>100 robotic cases), 2 (10-40 cases), 3 (no complete case/>4h at console), 4 (no robotic surgery), 5 (no experience in surgery) | Laboratory setting | 5 VR tasks | Face and Content for both | Standardised orientation/warm up | Strong correlation on test-retest reliability analysis of learning curve. 5th/6th and 6th/7th attempts^P^ | Construct**-***. Concurrent: Strong correlation of dV-T score and an expert rated non-validated Global Rating Scale score^P^ |  | 2b |
| Liss 2012^222^ | dVSS & dV-T | Observational - Non-randomised experimental study | 32 | 6 students (0 hours robotic surgery), 7 junior (<5 hours) and 6 senior resident/fellow (5-50 hours), attending surgeons: further categorised to 7 robotic novices (<5 hours) or 6 fellowship trained (>50 hours) | Laboratory setting | Same 3 VR tasks | Face and content both simulators- Yes. dVSS felt to be more realistic, and better to use*** |  |  | Construct -on tubes overall score dVSS***, dVT** Concurrent: Moderate to Strong correlation dVT and dVSS overall scores 3 tasks^P^ |  | 2b |
| Dubin 2017^165^ | dVSS & dV-T | Randomised controlled trial | 65 | 84% PY3, 20% fellow, 15% attending. Lap median 100 (range 0-2000), robotic median 50 (0-300) | Laboratory setting | Ring and Rail 1 |  | Randomised. Study not powered |  | Concurrent: dVT/dVSS Strong correlation with GEARS total scores^Sp^  Separate domain correlations varied from very weak (efficiency and master workspace range) to very strong (efficiency and time to complete) |  | 2a |
| Hoogenes 2018^83^ | dVSS & dV-T | Randomised controlled trial | 39 | 23 Junior trainees (medical students and junior residents), 16 experienced trainees (senior residents and fellows) | Laboratory setting | VR curriculum | Face and content both simulators -  dVSS group reported simulator provided significantly greater realistic experience | Randomised, powered study |  | Predictive of console performance in the lab: dVSS group had higher RACE scores on UVA model* dVSS scores in junior group statistically higher than dV-T, but similar in senior group |  | 1b |
| Hertz 2018^223^ | dVSS, dV-T, RobotiX Mentor | Observational - Non-randomised experimental study | 15 | 4 Interns, 4 PGY2, 2 Senior resident, 2 Attendings | Laboratory setting | 1 task on each simulator | Face: dVSS favoured over dV-T**. No difference between RobotiX Mentor and dVSS/dV-T.  Content dVSS>dV-T* and no difference between RobotiX Mentor and dVSS/dV-T |  |  |  |  | 4 |
| dV-Trainer | | | | | | | | | | | | |
| Kenney 2009^234^ | dV-T | Observational - Non-randomised experimental study | 26 | 19 Novices (<10 robotic cases)- 3 students, 11 residents and 5 attendings, 7 Experts (30+ robotic cases)- 2 residents and 5 senior staff | Laboratory setting | 4 VR tasks | Face and Content, but limitation noted in realism of needle driving |  |  | Construct** |  | 2b |
| Sethi 2009^240^ | dV-T | Observational - Non-randomised experimental study | 20 | Novice; Expert; Other: 15 novices (medical students, residents), 5 experts (>50 cases) | Laboratory setting | 3 VR tasks |  |  |  | Construct* in one exercise for time to complete and instruments out of view  Lower NASA TLX workload scores in experts |  | 2b |
| Lerner 2010^291^ | dV-T | Observational - Non-randomised experimental study | 23 | Medical students and interns | Laboratory setting | Dry model pre and post curriculum assessment.  dV-T training group vs da Vinci console training |  |  |  | Similar learning curve in pre to post assessment comparing console group vs VR |  | 3 |
| Korets 2011^193^ | dV-T | Observational - Non-randomised experimental study | 16 | Residents: 10 novices (<50 robotic cases), 6 Intermediate (50+) | Laboratory setting | 15 VR tasks | Face and content |  |  | Predictive of performance in the lab: VR simulation group showed significant* improvement in pre- and post-evaluation tests, non-VR group did not |  | 2b |
| Egi 2013^90^ | dV-T | Observational - Non-randomised experimental study | 12 | 3 Experts (post certification), 9 Intermediate (no licence with >50 lap surgeries) | Laboratory setting | 4 VR tasks | Face and Content - 100% participants stated Mimic dV-T is realistic and useful for training |  |  | Concurrent: Strong correlation of Mimic dV-T and da Vinci Surgical System on all 4 tasks when comparing time to complete^Sp^  Moderate correlation between dV-T suture sponge and OSATS score^Sp^  Construct: Stated significant difference between levels on simulator but "data not shown" |  | 2b |
| Cho 2013^237^ | dV-T | Randomised controlled trial | 11 | Expert-Certified General Surgeons, no robotic experience | Laboratory setting | 3 VR tasks |  | Randomised groups. Study not powered |  | Predictive of performance in the lab: VR intervention outperformed control group in the post-training test** on da Vinci robot | VR index score to indicate simulator competency= 1000 x 1/time x 1/distance). DV index competency score = 10/time x accuracy (errors used to help compute accuracy) Although no defined pass/fail benchmark. | 2a |
| Lendvay 2013^238^ | dV-T and SurgTrak™ Motion tracking and video capture | Randomised controlled trial | 51 | PGY 1-6, surgical fellows, faculty | Laboratory setting | 4 VR and 4 FLS tasks |  | Randomised  Powered study Standardised familiarisation/protocol |  | dV-T VR warm up does improve APMs/errors and the more experienced had improvements in time** and economy of motion** Decreased time and path length*-** | Benchmarked pass mark by experts | 2a |
| Kang 2014^235^ | dV-T | Observational - Non-randomised experimental study | 20 | 10 novices (general, obstetrics, urology residents- experienced laparoscopy, no robotics), 10 experienced surgeons (at least 10 robotic procedures) | Laboratory setting | Tube 3 module | Face and Content | Each novice was familiarised with console |  | Construct* |  | 2b |
| Ruparel 2014^239^ | dV-T | Observational - Non-randomised experimental study | 27 | Residents: 19 senior level (PGY3/4), 8 junior (PGY1/2) | Laboratory setting | 4 VR tasks |  | Training on the dVT console provided |  | Construct** on energy dissection module and on time to complete metric |  | 2b |
| Schreuder 2014^236^ | dV-T | Observational - Non-randomised experimental study | 42 | 15 Novices (no experience robotics), 14 Intermediates (5-50 cases performed), 13 Experts (70+ cases) | Laboratory setting | 4 VR tasks | Face |  |  | Construct*-*** |  | 2b |
| Kim 2015^241^ | dV-T | Observational - Non-randomised experimental study | 11 | 11 Urology residents, 3 fellows (No previous robotic experience) | Laboratory setting | Tube 3 module |  |  |  | Concurrent/Predictive of console performance in the lab: Tube 3 with UVA dry model (Limbs and Things, Bristol, England) using time to complete and end product. VR (intervention) group performed better on da Vinci console*-** |  | 3 |
| Whitehurst 2015^149^ | dV-T | Observational-prospective randomised study | 20 | Robotic naive: 7 residents, 8 fellows, 5 attending surgeons | Laboratory setting | 3 VR tasks  Post test cystostomy closure on live porcine |  | Randomised groups |  | No statistically significant difference between Mimic dV-T and FLS dry model group at live porcine post-curriculum test marked by GEARS i.e. dV-Trainer curriculum comparable to traditional da Vinci training. | Standard proficiency criteria for novices developed | 2a |
| Schommer 2017^242^ | dVT | Observational - Non-randomised experimental study | 27-32 | Other: Junior and senior residents | Laboratory setting | 4 VR tasks |  |  |  | No significant difference between junior and senior overall scores |  | 3 |
| RobotiX Mentor | | | | | | | | | | | | |
| Whittaker 2016^245^ | RobotiX Mentor | Observational - Non-randomised experimental study | 46 | 20 Novices (no operative experience, medical students), 15 Intermediates (some dual console experience, no independent cases), 11 Experts (at least 25 robotic procedures) | Laboratory setting | 9 VR tasks | Face/Content- Yes through questionnaire | Standardised familiarisation and practice |  | Construct: 17/25 metrics for expert vs. novice***, expert vs intermediate 7/25 metrics*-**, intermediate vs novice in 11/25 metrics*-*** |  | 2b |
| Watkinson 2018^243^ | RobotiX Mentor (3D systems, Simbionic Products, Cleveland, Ohio, USA) | Observational - Non-randomised experimental study | 123 | 84 Novices (no experience), 26 beginners (experienced assisting) and 9 advanced intermediates (1-99 cases independently), 4 experts (100+ independent robotic cases) | Laboratory setting | 5 VR tasks |  |  |  | Construct: Yes although advanced intermediates best performing on advanced exercises | Identified and benchmarked key metrics based on competent surgeons, defined here as advanced intermediate group, performances | 2c |
| Hovgaard 2018^246^ | RobotiX Mentor TM | Observational - Non-randomised experimental study | 22 | 11 robotic surgical novices, 11 experienced surgeons (>30 performed procedures) | Laboratory setting | Guided Vaginal Cuff closure | Content- 2 experienced surgeons assessed | Standardised instruction to all participants | Fair internal consistency for simulator metrics with discriminative ability  ^Cα^ | Construct*** | Pass/fail (75%) contrasting groups method. 4 novice passed (false positives) and 3 experienced failed (false negatives) | 2b |
| Alshuaibi 2019^247^ | RobotiX Mentor | Observational - Non-randomised experimental study | 37 | 8 Experts (>50 robotic cases), 11 Intermediates (5-50), 18 Beginners (<5) | Laboratory setting | FRS dome VR simulation | Face and Content |  |  | Construct** but experts and intermediates scored similarly.  Concurrent: RXM simulator with FRS metrics dry lab - FRS dome correlation varied from no (r=0.02 Puzzle dissection) to Strong (0.86 Ring Tower Transfer)^P^ | Defined threshold for RXM proficiency as 75th centile with mean expert scores of 77/100 | 2b |
| Leijte 2020^244^ | RobotiX Mentor (3D systems, Colorado, USA) | Observational - Non-randomised experimental study | 70 | 29 Novice (medical background no clinical experience), 26 Laparoscopic experienced group & 15 Robotic experience group (>10 procedures) | Laboratory setting | 3 VR tasks | Face and Content- Usability- good, Realism- Neutral to moderate positive opinion (lowest scores from behaviour of suture)  Didactic value- good | Standardised training from one instructor |  | Construct*-*** | Pass/Fail composite score developed | 2b |
| Scott 2020^143^ | RobotiX Mentor | Other: Delphi method | 13 to 22 | Delphi: 13 Experienced robotic surgeons from 4 specialties Validity evidence: 11 robotic novices and 11 experienced robotic surgeons (median 170 cases 30-600 range) | Laboratory setting | 6 VR tasks | Content-Delphi consensus |  | Internal consistency ranged unacceptable to good^Cα^ | Construct*-*** | Pass/Fail benchmarking using contrasting groups' method range 58-78% (Knot tying and horizontal defect respectively) | 2b |
| Ebbing 2021^102^ | RobotiX Mentor | Observational - Randomised experimental study | 51 | 17 Novices (console experience), 16 Intermediates (residents/fellows <100 cases), 18 Experts (at least 100 RARPs) | Laboratory setting | RARP | Face/Content- Yes in a questionnaire. Overall rated as valuable and feasible training tool. Developed from HD expert RARP videos. Defined phases, anatomy, visual cues, errors in to defined metrics incorporated to VR simulation | Powered study Novice familiarisation with RobotiX Mentor standardised |  | Construct: Bladder neck dissection -kinematics total moving distance, number of movements, instrument collisions and out of sight all lower in the expert group** Neurovascular bundle dissection*-** in kinematic and nerve sparing performance metrics. |  | 2b |
| Olsen 2021^103^ | RobotiX Mentor | Observational - Non-randomised experimental study | 27 | 10 Novices (assisted in RARP), 11 Intermediates (robotic but not RARP surgeons), 6 Experts (RARP surgeons) | Laboratory setting | RARP | Content - Yes- tested by RARP expert | Standardised orientation and participants blinded to metrics | Excellent test-retest reliability^Cα^  Three module scores had poor internal consistency reliability^Cα^  Unacceptable Internal consistency of the 6 metrics selected^Cα^ | Construct*-*** including Strong correlation between mean score of tests and total number of operations^P^  No difference between intermediates and experts | Pass/fail of RobotiX Mentor score defined using contrasting groups method | 2b |
| Raison 2021^173^ | RobotiX Mentor (3D Systems, Air-port City, Israel) | Randomised controlled trial | 26 | Novice: without experience in robotic surgery | Laboratory setting | Groups: VR basic, VR modular training and control (no training) |  | Randomised, not powered Familiarisation of novices with FRS tasks |  | Predictive of console performance in the lab: VR procedural training to cadaver task, more effective than both VR basic and control groups* Higher GEARS scores in VR Training groups compared to control** |  | 2a |
| ProMIS^TM^ hybrid surgical simulator | | | | | | | | | | | | |
| Chandra 2010^250^ | ProMIS(TM) hybrid surgical simulator | Observational - Non-randomised experimental study | 29 | 20 laparoscopic (<50 lap hours) novices, 9 substantial lap/robotic experience (50+) | Laboratory setting | Suturing |  |  | Good internal consistency between metrics of ProMIS^Cα^ | Construct*-*** |  | 2b |
| McDonough 2011^248^ | ProMIS(TM) hybrid surgical simulator | Observational - Non-randomised experimental study | 20 | 10 Novices (no robotic experience), 10 Experts (with robotic experience) | Laboratory setting | 3 FLS dry tasks | Face/Content- Yes | Standardised familiarisation and practice |  | Construct*** |  | 2b |
| Jonsson 2011^249^ | ProMIS(TM) hybrid surgical simulator - This study only the dry lab simulator part | Observational - Non-randomised experimental study | 24 | 19 Novices (13 consultants, 6 residents), 5 Experts (experienced robotic surgeons) | Laboratory setting | 4 Dry tasks |  | Standardised training |  | Construct*-*** |  | 2b |
| Robotic Surgery Simulator (RoSS) | | | | | | | | | | | | |
| Seixas-Mikelus 2010^251^ | Robotic Surgical Simulator (RoSS) console | Observational - Non-randomised experimental study | 30 | 6 Beginner/Novices (no experience with independent robotic procedures), 9 Competent (less than 150 robotic cases) and 15 Expert (Experienced surgeons 150+ robotic cases). | Laboratory setting | RoSS VR tasks | Face: majority rating several aspects' realism somewhat to very close |  |  |  |  | 4 |
| Seixas-Mikelus 2011^252^ | Robotic Surgical Simulator (RoSS) console | Observational - Non-randomised experimental study | 42 | 11 Beginner/Novices (no experience with independent robotic procedures),14 Competent (less than 150 robotic cases) and 17 Expert (Experienced surgeons 150+ robotic cases). | Laboratory setting | RoSS VR tasks | Content: Majority rated good-excellent |  |  |  |  | 4 |
| Chowriappa 2013^253^ | Robotic Surgical Simulator (RoSS) console | Observational - Non-randomised experimental study | 27 | 15 Novices (no experience)/ 12 experts (>150) in validation study | Laboratory setting; Other: Delphi | 5 RoSS tasks | Yes- Delphi consensus |  |  | Construct*** | Expert performance score range defined | 2b |
| Stegemann 2013^12^ | Robotic Surgical Simulator (RoSS) console | Randomised controlled trial | 53 | 9 medical students, 26 residents, 10 fellows, 8 practicing surgeons. All with no robotic experience | Laboratory setting | 3 Dry tasks | Yes- Survey | Randomised, powered study, Power calculation performed |  | Predictive of console performance in the lab: Intervention (VR) group who undertook FSRS curriculum outperformed control group in dry lab tasks* |  | 2a |
| Other Simulators | | | | | | | | | | | | |
| Balasundaram 2008^100^ | Sim Surgery version 1, Oslo, Norway | Observational - Non-randomised experimental study | 12 | 10 Novices- junior surgical residents (no MIS), 2 Experts (>50 robotic cases) | Laboratory setting | 5 VR tasks |  |  |  | Construct: Time to complete significant difference for two tasks only No significant improvement in error scores over 10 attempts |  | 2b |
| van Der Meijden 2010^101^ | SEP Robot (SimSurgery, Oslo, Norway) | Observational - Non-randomised experimental study | 16 to 17 | Novices (<50 MIS cases), Experienced (50+ MIS cases) | Laboratory setting | 1 VR task | Face |  |  | Construct (p=0.050) for tool tip trajectory only |  | 2b |
| Gavazzi 2011 ^292^ | SEP Robot (SimSurgery, Oslo, Norway) | Observational - Non-randomised experimental study | 30 | 18 Novices and 12 Experts | Laboratory setting | 2 VR tasks | Yes |  |  | Construct*-*** |  | 2b |
| Hung 2015^99^ | Novel simulation platform including Augmented and Virtual Reality | Randomised controlled trial | 42 | 15 novices (no surgical training), 13 intermediates (<100 robotic cases/any surgical training), 14 experts (100+) | Operating theatre; Laboratory setting | Robotic Partial Nephrectomy - AR =actual surgical footage - final module tumour resection has full VR task embedded | Face & Content- Yes |  |  | Construct: expert and intermediate vs novices*-** Concurrent: VR task and GEARS**-*** |  | 2b |
| Witthaus 2020^84^ | 3D printed hydrogel simulation- Clinically relevant performance metrics of simulation (CRPMS) | Observational - Non-randomised experimental study | 14 | Novice; Expert; Other: 9 novices (caseload <50), 5 experts (caseload >500) | Laboratory setting | Nerve Sparing RARP 3D printed hydrogel model | Face - Survey high fidelity 3D printed model Content- Pilot testing and revision with construct validity |  |  | Construct: task completion, surgical margin results*, nerve forces*-** Concurrent: Strong to very strong inverse correlation Total GEARS and force sensitivity domain with lower nerve forces^Sp^ Strong inverse correlation RACE scores and UVA leak rate^Sp^ |  | 2b |
| Ghazi 2021^52^ | 3D printed hydrogel simulation- Clinically Relevant Objective Metrics (CROMS) | Observational - Non-randomised experimental study | 43 | 27 Novices (1-30 upper tract robotic cases), 16 Experts (>150) | Laboratory setting | Robot-assisted partial nephrectomy (RAPN) 3D printed hydrogel model | Face - Survey high fidelity 3D printed model Content- Pilot testing and revision with construct validity | Pilot testing and accuracy tests with anatomical (CT scan) and mechanical validation (porcine kidney) tests |  | Construct*** GEARS and CROMS (except PSM).  Concurrent: Strong correlation between CROMS (exc. PSM) and total GEARS^P^ |  | 2b |
| Butterworth 2021^172^ | Versius trainer | Observational - Non-randomised experimental study | 17 | All senior surgeons. Novice (<5 procedures), Intermediate (5-29), Expert (30 +) | Laboratory setting | VR simulation |  |  |  | Learning curve: Decrease in path lengths with further attempts |  | 3 |
| Cowan 2021^254^ | da Vinci SimNow (VR) vs. da Vinci system event data recorder (dry lab) | Randomised controlled trial | 17 | 6 Experts (=>300 cases) 11 trainees (<300 cases) | Laboratory setting | UVA VR task UVA Dry model task by 3-DMed |  |  |  | Concurrent: VR and Dry Lab metrics selected. 8 metrics moderate to strong correlation^P^  Construct: VR 5/22 metrics distinguished expertise* compared to dry APMs 14/22*  Experts had lower Index of Cognitive Activity i.e. lower workload * |  | 2a |
| Turner 2021^104^ | “da Vinci simulator backpack” with 3D Systems (3DS) simulator by Simbionix | Observational - Non-randomised experimental study | 32 | Other: Residents | Laboratory setting | Robotic Hysterectomy | Weak face-Stated high fidelity VR simulation | Standardised curriculum |  | Learning curve: Improved metrics with practice |  | 3 |
| Simmonds 2021^293^ | Mimic Technologies- all their VR Robotic Surgery Simulators (e.g. dV-T, dVSS) | Observational - Non-randomised experimental study | 77 | Other: Residents to surgeons | Laboratory setting | 17, 648 sessions analysed from 77 students |  |  |  |  | Defined MScore Proficiency Index© (MPI©) indicating single, dynamic reference number that normalize all scores from all exercises to a mean value consistent with an average user's score | 2c |

| Table S7: Non-Simulator Automated Performance Metrics | | | | | | | | | | | | |
| --- | --- | --- | --- | --- | --- | --- | --- | --- | --- | --- | --- | --- |
| Study | Simulator platform | Study type | Participant  No. | Expertise level | Setting | Task | Test  Content | Response process | Internal Structure | Relationship other variables | Consequence | L  O  E |
| da Vinci Kinematic/System Event Recorders | | | | | | | | | | | | |
| Verner 2003^256^ | da Vinci Application Programming Interface (API) | Observational - Non-randomised experimental study | 6 | 2 Novices and 4 Experts | Laboratory setting | Bead placement on peg |  |  |  | Construct: flight path*** and time* |  | 2b |
| Hernandez 2004^189^ | da Vinci Application Programming Interface (API) | Observational - Non-randomised experimental study | 13 | 6 Experienced group (>100 lap chole and clinical practice intracorporeal knot tying), 7 non-experienced (<50 lap choles and no advanced lap experience) | Laboratory setting | Synthetic small bowel anastomoses (Limbs and Things) |  |  |  | Learning curve: Time***, number of movements*and path length* all improved with successive attempts. |  | 3 |
| Narazaki 2006^260^ | da Vinci Application Programming Interface (API) | Observational - Non-randomised experimental study | 7 | Novice | Laboratory setting | Suturing, Needle passing, Bimanual carrying |  |  |  | Learning curve: Shorter travelling distance of instruments post training compared to pre for Needle passing** and Suture Tying** and 30% change in mean absolute relative phase (MARP) i.e. bimanual dexterity measurable improvement** |  | 3 |
| Nazaraki 2007^257^ | da Vinci Application Programming Interface (API) | Observational - Non-randomised experimental study | 24 | Novice; Expert | Laboratory setting | Rope running, Bimanual carrying, Needle passing |  |  |  | Construct: Yes  Significant differences task completion times and mean absolute relative phase (MAPR) values i.e. bimanual dexterity)*  No significant difference in total instrument travel distance |  | 2b |
| Judkins 2009^258^ | da Vinci Application Programming Interface (API) | Observational - Non-randomised experimental study | 10 | 5 Novices (medical students), 5 Experts (advanced laparoscopists) | Laboratory setting | Suturing, Needle passing, Bimanual carrying |  |  |  | Construct validity: Task completion time, speed, curvature*-***  Learning curve: Novices APMs significantly improved after training |  | 2b |
| Suh 2011^89^ | da Vinci Application Programming Interface (API) | Observational - Non-randomised experimental study | 15 | Novices (Medical students) | Laboratory setting | Suturing |  |  |  | Learning curve: time to complete*** average instrument speed**, movement curvature* and EMG activation*.  Concurrent- Kinematics improved correlating with OSATS score improvements |  | 3 |
| Vedula 2016^198^ | da Vinci Application Programming Interface (API) | Observational - Non-randomised experimental study | 18 | 14 novices (surgical trainees) and 4 experts (attendings). From previous data set (Kumar et al. 2012) | Laboratory setting | Needle passing, suturing |  |  |  | Construct: task, maneuver and gesture level for all 3 metrics (time, path length, number of movements)*  Logistic regression- No significant difference in skill level predictions using metrics between task, maneuver and gesture level. |  | 2b |
| Lefor 2021^261^ | da Vinci Application Programming Interface (API) (ROVIMAS software) | Observational - Non-randomised experimental study | 8 (JIGSAWS) | Novice; Intermediate; Expert | Laboratory setting | JIGSAWS- Suturing, knot tying, needle passing |  |  |  | Learning curve: Path length significantly lower on 5th compared to 1st attempt for needle passing* Qualitative analysis shows a learning curve only for suturing. | Cumulative sum analysis suggests completion of the learning curve for suturing by trial 4 | 3 |
| Hung 2018^157^ | dVLogger | Observational - Non-randomised experimental study | 20 | 10 Novices (<100) and 10 Experts (100+ cases) | Operating theatre | RARP |  | Anonymised data |  | Construct*  Concurrent: Very weak to moderate correlation kinematics with GEARS^Sp^  Event metrics no statistical association |  | 2b |
| Oh 2018^154^ | dVLogger | Observational - Non-randomised experimental study | 11 | 1 chief resident, 4 advanced robotics and lap fellows, 6 faculty attendings performing 25 UVAs | Operating theatre | UVA |  |  |  | Concurrent: Total GEARS score discriminated APMs between higher and lower performing groups  Dependent on raters i.e. crowd source did distinguish 3, some experts none, 1 expert distinguished 8 APMs as better performing. |  | 2b |
| Chen 2019^37^ | dVLogger | Observational - Non-randomised experimental study | 26 | Surgeons (varying expertise), 78 cases. In this Super expert defined as >2000 prior robotic cases vs non super expert as <2000 (based on prior work), also states <100 is novice and >100 is expert | Operating theatre | RARP | Yes based on prior work |  |  | Construct/Predictive*: surgeon experience affected the greatest number of APMS. Urinary continence recovery associated with surgeon's prior experience  BMI, pelvic dimensions and prostate size also significantly affected APMs |  | 2b |
| Hung 2019^259^ | dVLogger | Observational - Non-randomised experimental study | 14 | 10 Experts (100-750 cases), 4 Super experts (2100-3500 cases) | Operating theatre | RARP |  |  |  | Construct/Predictive: Super experts performed better in APMS* and with better clinical outcomes (LN yield**, less blood loss**, fewer readmissions*  Similar non-significant trend for oncological and functional outcomes |  | 2b |
| Nguyen 2020^255^ | da Vinci systems events data recorder and Task-Evoked Pupillary Response (TEPR) measuring Cognitive mental workload by Tobii Pro Glasses 2 (Tobii Technology, Inc.) | Observational - Non-randomised experimental study | 26 | 9 Novices (no experience), 9 Intermediates (<100 cases), 8 Experts 8 (100+) | Laboratory setting | 4 Dry model tasks |  | Anonymised recordings for eye tracking sent for data processing to produces Index of Cognitive Activity (ICA) |  | Construct: time active of dominant and non-dominant instruments*, Linear velocity of instruments & camera*, Angular velocity of dominant instrument* Task Evoked Pupillary Response (TEPR) and Index of Cognitive Activity Decrease in ICA as experience increased in 3/4 tasks*-**  Correlation APMs and TEPR: Strong inverse correlation between APMs and TEPR for experts i.e. slower movements when higher cognitive workload, whilst novices strong positive correlation i.e. move faster in the same cognitive experience^Sp^ |  | 2b |
| Hung 2021^36^ | da Vinci systems event data recorder | Observational - Non-randomised experimental study | 20 | Faculty surgeons | Operating theatre | RARP |  |  |  | Predictive: Overall: weak to moderate correlation with clinical characteristics ^Sp^ APMs are significant and independent predictors of continence (10/20 were dexterity metrics during UVA). Using APMs and clinical factors random forest model AUC 0.75 and 0.67 for 3- and 6- month continence prediction.  Clinical factors confound surgeon APMs but 20 variables of importance affecting 3 month continence were all APMs, 11/20 were related to UVA. |  | 3 |
| Ghodoussipour 2021^35^ | da Vinci systems event data recorder ("Intuitive Surgical custom recording tool") | Observational - Non-randomised experimental study | 27 | 10 Trainees (Residents, fellows or RAPN <150), 7 Experts (faculty with >150 cases) | Operating theatre | RAPN |  |  |  | Construct* faster dominant movement and greater dominant instrument usage  Concurrent: APMs significantly correlated with R.E.N.A.L nephrotomy score, EBL and warm ischaemia time |  | 2b |
| Cowan 2021^254^ | da Vinci systems event data recorder from Intuitive (dry lab) vs. da Vinci SimNow (VR) | Randomised controlled trial | 17 | 6 Experts (=>300 cases) 11 Trainees (<300 cases) | Laboratory setting | UVA VR UVA by 3-Dmed |  | Randomised, low sample size |  | Concurrent: corresponding VR and Dry Lab 8 metrics moderate to strong correlation ^P^  Construct: VR 5/22 metrics distinguished expertise* compared to dry APMs 14/22* Concurrent/construct: Experts had lower Index of Cognitive Activity i.e. lower workload * |  | 2a |
| Electromagnetic motion tracker sensor (TrakStar; Ascension Technologies, Milton, VT, USA) | | | | | | | | | | | | |
| Tausch 2012^262^ | Electromagnetic motion tracker sensor (TrakStar; Ascension Technologies, Milton, VT, USA) | Observational - Non-randomised experimental study | 10 | 5 Novices (PGY1-4), 5 Experts (faculty) | Laboratory setting | 3 Dry model tasks |  | Standardised practice and orientation |  | Construct*-***time and economy of motion |  | 2b |
| Nisky 2013^265^ | Electromagnetic motion tracker sensor (TrakStar; Ascension Technologies, Milton, VT, USA) | Observational - Non-randomised experimental study | 13 | 10 engineering graduates with no experience, 3 experienced urology surgeons with >100 RAS cases | Laboratory setting | "Reach and Reversal" (two simple movements) |  |  |  | Construct*** experts move more smoothly |  | 2b |
| Nisky 2014^264^ | Electromagnetic motion tracker sensor (TrakStar; Ascension Technologies, Milton, VT, USA) and a force sensor (Nano-17; ATI Industrial Automation, Apex, NC, USA) | Observational - Non-randomised experimental study | 16 | 10 Novice non-medical users no da Vinci; 6 Expert (experienced robotic surgeons) | Laboratory setting | "Reach and Reversal" (two simple movements) |  |  |  | Construct*** experienced surgeons had smaller errors than novices |  | 2b |
| Walker 2017^263^ | Electromagnetic motion tracker sensor (TrakStar; Ascension Technologies, Milton, VT, USA) | Observational - Non-randomised experimental study | 8 | 7 PGY2-5 and 1 first year fellow | Laboratory setting | Female pelvis simulation model- Bleeding simulation |  | Expert reviewers of metrics |  | Wide variation in jerkinees, path length, idle time, bimanual dexterity |  | 3 |
| Hutchins 2018^209^ | Electromagnetic motion tracker sensor (TrakStar; Ascension Technologies, Milton, VT, USA) | Observational - Non-randomised experimental study | 16 | 10 residents, 6 fellows/faculty | Laboratory setting | 2 FLS tasks: Peg transfer, Precision cutting |  |  |  | Construct* Lower path length in more experienced group |  | 2b |
| Other APM recording devices | | | | | | | | | | | | |
| Lendvay 2013^238^ | SurgTrak™ Motion tracking and video capture & dV-T | Randomised controlled trial | 51 | PGY 1-6, surgical fellows, faculty | Laboratory setting | 4 FLS modules (block transfer, suturing, ring tower, rotating pegboard) and 4 VR tasks (Pick and place, ring walk level 1, pegboard level 1 and 3) |  | Randomised  Powered study Standardised familiarisation/protocol |  | Construct: dV-T VR warm up does improve APMs/errors and the more experienced had improvements in time** and economy of motion** Decreased time and path length*-** |  | 2a |
| Suh 2016^214^ | Electromyography (EMG) of forearm muscles and Kinematic instrument movement | Observational - Non-randomised experimental study | 15 | Other: 12 medical students, 3 residents | Laboratory setting | Robotic suturing whilst being distracted to different levels |  |  |  | Distraction affects performance*-** when measuring objective kinematic and EMG measurements as well as subjective NASA Task Load Index (NASA-TLX) |  | 3 |
| Gomez 2016^105^ | Instrument vibrations and forces | Observational - Non-randomised experimental study | 13 | 8 Novice robotic surgeons, 5 Experienced robotic (50-2400 cases) | Laboratory setting | 3 Dry tasks: Peg Transfer, Needle Passing. Suturing |  |  |  | Construct: vibrations and forces differed over all 3 tasks differ*-***  Concurrent: Negative correlation of with vibration/forces and completion time with OSATS/GEARS combined GRS*-** (excluding suturing task- no significant correlation)  Receiver operating curve analysis also how vibration and force magnitudes with completion time in skill prediction models improves objective classification of subjects as novice or experienced in all tasks ranging 85-100% sensitivity, 75-100% specificity |  | 2b |
| Jiang 2017^294^ | Instrument trajectory and dynamic time warping | Observational - Non-randomised experimental study | 10 | Novice; Expert | Laboratory setting | Peg transfer |  |  |  | Construct*-** "optimum" trajectory and divided it into different subtasks. Then, the optimum trajectory was compared with the real trajectories of the participants. Dynamic Time Warping was used to assess the similarity between optimal and actual. |  | 3 |
| Busch 2019^295^ | Pressure-measuring and image-processing device | Observational - Non-randomised experimental study | 13 | Novice | Laboratory setting | Suturing- intestinal anastomosis model |  |  |  | Knot tension was higher in robotic assisted vs. laparoscopy* |  | 3 |
| Beulens 2020^296^ | Software (Kinovea) | Observational - Non-randomised experimental study | 1 | Expert | Operating theatre | RARP |  |  |  | Attempted to evaluate relation between movements and post-op urinary continence. Results show on average it was only able to automatically assess track 22% of surgical duration | Kinovea only deemed valid if able to track >80% of duration of surgery. However, | 3 |

| Table S8: Artificial Intelligence Studies | | | | | | | | | | |
| --- | --- | --- | --- | --- | --- | --- | --- | --- | --- | --- |
| Study | Participant  No. | Setting S/D/OR (dataset) | Tasks No. | Dataset size | AI task | Model  ML/DL | Model  description | Model input | Model output | Performance |
| Jog 2011^266^ | 17 | S (private) | 1 | 41 trials | C | ML | SVM | K | SL | Acc: 65-87.5% |
| Kumar 2011^267^ | 8 | D (private) | 3 | 134 trials | C | ML | SVM | K, F, E | SL | Acc: 88.2-91.7% |
| Kumar 2012^279^ | 12 | D (private) | 4 | UTD | C | ML | SVM | K, E | SL | Acc: 76.24-83.33% |
| Tao 2012^268^ | 8 | D (JIGSAWS) | 3 | 139 trials | C | ML | HMM | K | SL | Acc: 46.2-97.4% |
| Ahmidi 2013^280^ | 8 \| 18 | D (JIGSAWS) | 2 | 39 trials \| 110 trials | C | ML | DCC, CSM | K | SL | Acc: 91.12% |
| Malpani 2015^281^ | 18 | D (private) | 2 | 135 trials | R | ML | SVM, LiR | K | AS | RMSE < SD of GRS |
| Vedula 2016^198^ | 18 | D (private) | 2 | 135 trials | C | ML | LoR | K | SL | AUC: 0.70-0.79 |
| Gomez 2016^105^ | 13 | D (private) | 3 | 156 trials | R | ML | Stepwise Regression | K, F | AS | R^2^: 0.76-0.85 |
| Brown 2017^152^ | 37 | D (private) | 1 | 110 trials | C, R | ML | SVM, ER, RT, RF, KNN | K, F | AS | Acc: 51.7-75% |
| Parmar 2017^131^ | 8 | D (JIGSAWS) | 3 | 103 videos | R | DL | CNN, LSTM | V | AS | SCC: 0.60-0.84 |
| Zia 2018^120^ | 8 | D (JIGSAWS) | 3 | 103 videos | C, R | ML | DCT, DFT, ApEn, NN | K | SL, AS | Acc 77.4-100%, SCC: 0.42-0.78 |
| Ershad 2018^125^ | 14 | S (private) | 3 | 84 trials | C | ML | LoR | K, O | AS | Acc: 69-89% |
| Fard 2018^269^ | 8 | D (JIGSAWS) | 2 | 103 videos | C | ML | KNN, LoR, SVM | K | SL | Acc : 62.6-89.9% |
| Dubin 2018^107^ | 74 | S (private) | 2 | UTD | R | ML | LoR | O | AS | no test data |
| Law 2018^297^ | 12 | OR (private) | 1 | 12 videos | C | DL | CNN | V | SL | Acc: 83.33-100% |
| Ershad 2018^126^ | 14 | S (private) | 2 | 84 trials | C | ML | Gaussian naive Bayes | K, O | SL | Acc: 75-100% |
| Xiang 2018^132^ | 8 | D (JIGSAWS) | 3 | 103 videos | R | DL | CNN | V | AS | SCC: 0.03-0.68 |
| Doughty 2018^121^ | 8 | D (JIGSAWS) | 3 | 103 videos | O | DL | CNN | V | O | Pairwise precision: 70.2% |
| Wang 2018 (EMBS)^137^ | 8 | D (JIGSAWS) | 3 | 139 trials | C | DL | TCN | K | SL | Acc: 96% and 100% |
| Wang 2018 (IJCARS)^134^ | 8 | D (JIGSAWS) | 3 | 139 trials | C | DL | TCN | K | SL | Acc: 91.3-95.4% |
| Baghdadi 2019^106^ | 1 | OR (private) | 1 | 20 videos | C | ML | LoR | V | AS | Accuracy: 83.3% |
| Ershad 2019^122^ | 14 | S (private) | 2 | 84 trials | C | ML | SVM | K | O | Acc: 47.79-98.5% |
| Fawaz 2019^118^ | 8 | D (JIGSAWS) | 3 | 103 videos | C, R | DL | TCN | K | SL, AS | Acc: 93.2-100%, SCC 0.57-0.66 |
| Ogul 2019^129^ | 8 | D (JIGSAWS) | 3 | 139 trials | O | DL | LSTM | K | O | Acc: 63.5-79.6% |
| Liu 2019^116^ | 1 | OR (private) | 1 | 57 videos | R | DL | CNN | V | AS | SCC: COF 0.647, OTS 0.55, OPS 0.41 |
| Pan 2019^270^ | 8 | D (JIGSAWS) | 3 | 103 videos | R | DL | CNN, GNN | V | AS | SCC: 0.36-0.75 |
| Anh 2019^135^ | 8 | D (JIGSAWS) | 3 | 139 trials | C | DL | DFT, DCT, CNN, CNN, LSTM, PCA, Codebook, LSTM, Autoencoder | K | SL | Acc: 90.17-96.84% |
| Nguyen 2019^136^ | 15 | D (private) | 2 | 75 trials | C | DL | TCN, LSTM | K | SL | Accuracy: 98.2% |
| Hung 2019^115^ | 8 | OR (private) | 1 | 100 cases | C | DL | MLP | K, E, O | CO | C-index: 0.6 |
| Funke 2019^271^ | 8 | D (JIGSAWS) | 3 | 103 videos | C | DL | CNN | V | SL | Acc: 95.8-100% |
| Brown 2020^284^ | UTD | D (private) | 1 | UTD | C | ML | LoR | K, E | SL | Acc: 80-98% |
| Wang 2020^127^ | 8 | D (JIGSAWS) | 3 | 103 videos | C, R, O | DL | CNN, LSTM, TCN | V | SL, AS, O | SCC 0.75-0.89, Acc: 97.5-100% |
| Tang 2020^273^ | 8 | D (JIGSAWS) | 3 | 103 videos | R | DL | CNN | V | AS | SCC: 0.69-0.71 |
| Lee 2020^117^ | UTD | D/OR (private) | 1 | 23 videos | C | DL | CNN | V | SL | Acc: 83% |
| Ming 2020^272^ | 8 | D (JIGSAWS) | 3 | 139 trials | C | ML | SVM | K | SL | Acc: 74.66-83.71% |
| Wu 2020^124^ | 7 | D (private) | 6 | 26 sessions | C | ML | LoR, Naïve Bayes, SVM | O | O | Acc: 72.5% |
| Chen 2021^33^ | 17 | OR (private) | 1 | 68 surgeries | C | ML | RF | K, E | SL | Acc: 67.24-80.1% |
| Liu 2021^110^ | 8 \| UTD | D (JIGSAWS) \| OR (private) | 3 \| 1 | 103 \| 20 videos | R | DL | CNN, GNN | V, K, O | AS | SCC: 0.45-0.83 \| 0.57 |
| Lyman 2021^274^ | 2 | D (private) | 1 | 25 trials | C | ML | LiR | K, E | SL | Acc: 89.3% |
| Yu 2021^275^ | 8 | D (JIGSAWS) | 3 | 103 videos | R | DL | CNN | V | AS | SCC: 0.84-0.86 |
| Takacs 2021^283^ | UTD | D (private) | 2 | UTD | C | ML | Neuro-fuzzy system | F | SL | UTD |
| Lavanchy 2021^108^ | UTD | OR (private) | 1 | 242 videos | R | DL | LiR | V | AS | Acc: 70-87% |
| Zhang 2021^276^ | 8 | D (JIGSAWS) | 1 | 39 videos | S, O | DL | CNN | V | SL, O | Acc: 100% |
| Soleymani 2021^130^ | 8 | D (JIGSAWS) | 3 | 103 videos | C | DL | CNN, TCN | V | SL | Acc: 97.27% |
| Juarez-Villalobos 2021^278^ | 8 | D (JIGSAWS) | 3 | 139 trials | C | ML | KNN, RF, SVM | K | SL | AcC: 83-100% |
| Lajko 2021^282^ | 8 | D (JIGSAWS) | 3 | 103 videos | C | DL | LSTM, CNN, LSTM, Autoencoder, FDT | V | SL | Acc: 81.58- 84.23% |
| Hung 2021^36^ | 20 | OR (private) | 1 | 193 cases | C | ML | RF | K, E | CO | AUC 0.67-0.74 |
| Li 2022^111^ | 8 \| UTD | D (JIGSAWS) \| OR (Heichole) | 3 \| 1 | 103 \| 33 videos | R | DL | CNN, LSTM | V | AS | SCC: 0.72-0.93 \| 0.46 |
| Soleymani 2022^277^ | 8 \| 5 | D (JIGSAWS) \| D (private) | 3 \| 1 | 139 \| 30 trials | C | ML | cPCA, t-SNE | K | SL | UTD |
| Ma 2022^114^ | 21 | OR (private) | 1 | 80 cases | C | DL | LSTM, Transformer | O | CO | AUC 0.68-0.77 |
| Benmansour 2023^119^ | 8 | D (JIGSAWS) | 3 | 139 trials | R | DL | CNN, LSTM | K | AS | SCC 0.65-0.82 |
| Anastasiou 2023^128^ | 8 | D (JIGSAWS) | 3 | 103 videos | R | DL | CNN, TCN, Transformer | V | AS | SCC: 0.65-0.89 |
| Kiyasseh 2023^138^ | 42 | OR | 2 | 158 videos | C | DL | Transformer | V | SL | AUC: 0.719-0.880 |

Table S9: Summary of all other tools identified in assessment domains

| Global Rating Scale Tools | | | | | | | | | |
| --- | --- | --- | --- | --- | --- | --- | --- | --- | --- |
| Tool | Study type | Setting | Test  Content | Response process | Internal Structure | Relationship to other variables | Consequences | LoE | LoR |
| **ARCS**^205^ | 1 Observational | 1 Lab | Consensus development by 3 expert da Vinci Surgical System training professionals | Yes | Moderate to high inter-rater reliability | **Construct validity** |  | Level 2b | Level 3 recommendation |
| **Modified Surgical**  **Skill Index**^54^ | 1 Observational | Lab |  | Yes |  | **Construct validity** |  | Level 2b | Level 3 recommendation |
| **RO-SCORE**^206^ | 1 Observational | 1 Lab & OR |  | Yes |  | **Concurrent validity with cognitive load tool** |  | Level 3 | Level 4 recommendation |
| **SARMS**^207^ | 1 Observational | 1 Lab |  | Yes | **Intra-rater reliability-** Moderate  **Internal consistency-**Good to excellent | **Construct validity** |  | Level 3 | Level 4 recommendation |
| Procedure- and Task-Specific Tools | | | | | | | | | |
| **A-OSATS**^85^ | 1 Delphi with video-rater analysis | Delphi and OR | Delphi consensus and thorough literature review | Yes | **Inter-rater reliability**  Excellent  **Intra-rater reliability**  Strong | **Concurrent Validity with OSATS**  **Construct validity** | Arbitrary cut offs indicating expertise level | Level 2a | Level 3 recommendation |
| **UVA Evaluative score**^82^ | 1 RCT | Lab | Weak – created by expert surgeon | Yes | **Inter-rater reliability**  0.68 (UTD statistical test) | **Concurrent validity with GEARS**  **Construct validity** |  | Level 2a | Level 3 recommendation |
| **CASE**^56^ | 1 Delphi | Delphi and OR | Delphi consensus | Yes | **Inter-rater reliability**  0.68-0.81 (linearly weighted agreement) |  |  | Level 2b | Level 3 recommendation |
| **DART**^96^ | 1 Delphi with video-rater analysis | Delphi and OR | Delphi consensus | Yes | **Inter-rater reliability:**  Fair to Good | **Construct validity** |  | Level 2b | Level 3 recommendation |
| **EASE**^208^ | 1 Delphi with video-rater analysis | Delphi and OR | Delphi consensus | Yes | **Inter-rater reliability:**  Moderate to high | **Concurrent validity with RACE**  **Construct validity** |  | Level 2b | Level 3 recommendation |
| **PACE** | 1 Delphi  1 Observational | 1 OR  1 Delphi and OR | Delphi consensus | Both | **Inter-rater reliability:**  Fair to excellent | **Construct validity**^66^ |  | Level 2b^66^  Level 4^64^ | Level 3 recommendation |
| **PLACE**^79^ | 1 Delphi | Delphi consensus | Delphi consensus | Yes | **Inter-rater reliability:**  Low to moderate | **Concurrent validity** |  | Level 2b | Level 3 recommendation |
| **Proficiency Score**^80^ | 1 Observational | OR | Delphi | Yes | **Inter-rater reliability:**  Fair to excellent | **Concurrent validity with GEARS**  **Construct validity** |  | Level 2b | Level 3 recommendation |
| **RHAS**^65^ | 1 Observational | Delphi and operating room | Delphi consensus | Yes | **Inter-rater reliability:**  Good | **Construct validity** |  | Level 2b | Level 3 recommendation |
| **SpaN**^74^ | 1 Delphi | Delphi consensus | Delphi consensus | Yes | **Inter-rater reliability:**  75% linearly weighted percent agreement |  |  | Level 2b | Level 3 recommendation |
| **Competency Assessment Tools**  Tools are for separate procedures/tasks. | 1 Delphi  2 Observational | Delphi and OR | Only one tool had good test content validity^87^ | Yes 2 of 3^58,87^ | **Internal Consistency**:  High^87^ | **Contrast validity**^87^ |  | Level 3^87^  Level 4^58,59^ | Level 4 recommendation |
| **GAS**^60^ | 1 Case series | OR |  | Yes |  |  | CUSUM defined proficiency level | Level 3 | Level 4 recommendation |
| **Pancreaticojejunostomy score card**^86^ | 1 Observational | OR |  | Yes | **Inter-rater reliability:**  Weak to Moderate with PJV and OSATS (combined within same score  **Intra-rater reliability**  Strong to very strong | **Predictive validity** |  | Level 3 | Level 4 recommendation |
| **RAPN-Specific Scoring System**^71^ | 1 Observational | Lab |  | Yes |  |  |  | Level 3 | Level 4 recommendation |
| **RARP Assessment Score** | 2 Observational | 2 OR | Consensus using HFMEA^70^ | Yes^70^ | **Inter-rater reliability:**  No/Poor^70^ |  | Learning curve defined in 5 out of 17 steps^70^ | Level 3^70^  Level 4^69^ | Level 4 recommendation |
| **RARP procedure-specific scoring scale**^73^ | 1 Observational | OR | Expert panel development | Yes |  |  | Defined pass mark | Level 3 | Level 4 recommendation |
| **rOSATS**^92^ | 1 RCT | Lab |  | Yes |  |  |  | Level 3 | Level 4 recommendation |
| Non-Simulator Automated Performance Metrics | | | | | | | | | |
| **Instrument vibrations and forces**^105^ | 1 Observational | Lab |  |  |  | **Construct validity**  **Concurrent with GRS** |  | Study Level 2b | Level of recommendation 3 |

Table S10: PRISMA Abstract and Full Checklist

| **Section and Topic** | **Item #** | **Checklist item** | **Reported (Yes/No)** |
| --- | --- | --- | --- |
| **TITLE** | | |  |
| Title | 1 | Identify the report as a systematic review. | Yes |
| **BACKGROUND** | | |  |
| Objectives | 2 | Provide an explicit statement of the main objective(s) or question(s) the review addresses. | Yes |
| **METHODS** | | |  |
| Eligibility criteria | 3 | Specify the inclusion and exclusion criteria for the review. | Yes |
| Information sources | 4 | Specify the information sources (e.g. databases, registers) used to identify studies and the date when each was last searched. | Yes |
| Risk of bias | 5 | Specify the methods used to assess risk of bias in the included studies. | Yes |
| Synthesis of results | 6 | Specify the methods used to present and synthesise results. | Yes |
| **RESULTS** | | |  |
| Included studies | 7 | Give the total number of included studies and participants and summarise relevant characteristics of studies. | Yes |
| Synthesis of results | 8 | Present results for main outcomes, preferably indicating the number of included studies and participants for each. If meta-analysis was done, report the summary estimate and confidence/credible interval. If comparing groups, indicate the direction of the effect (i.e. which group is favoured). | Yes |
| **DISCUSSION** | | |  |
| Limitations of evidence | 9 | Provide a brief summary of the limitations of the evidence included in the review (e.g. study risk of bias, inconsistency and imprecision). | Yes |
| Interpretation | 10 | Provide a general interpretation of the results and important implications. | Yes |
| **OTHER** | | |  |
| Funding | 11 | Specify the primary source of funding for the review. | Yes- N/A |
| Registration | 12 | Provide the register name and registration number. | Yes |

*From:*  Page MJ, McKenzie JE, Bossuyt PM, Boutron I, Hoffmann TC, Mulrow CD, et al. The PRISMA 2020 statement: an updated guideline for reporting systematic reviews. BMJ 2021;372:n71. doi: 10.1136/bmj.n71

For more information, visit: <http://www.prisma-statement.org/>

| **Section and Topic** | **Item #** | **Checklist item** | **Location where item is reported** |
| --- | --- | --- | --- |
| **TITLE** | | |  |
| Title | 1 | Identify the report as a systematic review. | Page 1 |
| **ABSTRACT** | | |  |
| Abstract | 2 | See the PRISMA 2020 for Abstracts checklist. | Labelled as such in a supplementary file |
| **INTRODUCTION** | | |  |
| Rationale | 3 | Describe the rationale for the review in the context of existing knowledge. | Page 4 & 5 |
| Objectives | 4 | Provide an explicit statement of the objective(s) or question(s) the review addresses. | Page 5 |
| **METHODS** | | |  |
| Eligibility criteria | 5 | Specify the inclusion and exclusion criteria for the review and how studies were grouped for the syntheses. | Page 6 |
| Information sources | 6 | Specify all databases, registers, websites, organisations, reference lists and other sources searched or consulted to identify studies. Specify the date when each source was last searched or consulted. | Page 5 |
| Search strategy | 7 | Present the full search strategies for all databases, registers and websites, including any filters and limits used. | Figure 1 |
| Selection process | 8 | Specify the methods used to decide whether a study met the inclusion criteria of the review, including how many reviewers screened each record and each report retrieved, whether they worked independently, and if applicable, details of automation tools used in the process. | Page 6 |
| Data collection process | 9 | Specify the methods used to collect data from reports, including how many reviewers collected data from each report, whether they worked independently, any processes for obtaining or confirming data from study investigators, and if applicable, details of automation tools used in the process. | Page 6-7 |
| Data items | 10a | List and define all outcomes for which data were sought. Specify whether all results that were compatible with each outcome domain in each study were sought (e.g. for all measures, time points, analyses), and if not, the methods used to decide which results to collect. | Page 6-7 |
|  | 10b | List and define all other variables for which data were sought (e.g. participant and intervention characteristics, funding sources). Describe any assumptions made about any missing or unclear information. | Page 6-7 |
| Study risk of bias assessment | 11 | Specify the methods used to assess risk of bias in the included studies, including details of the tool(s) used, how many reviewers assessed each study and whether they worked independently, and if applicable, details of automation tools used in the process. | Page 7 |
| Effect measures | 12 | Specify for each outcome the effect measure(s) (e.g. risk ratio, mean difference) used in the synthesis or presentation of results. | Page 7 and Table 1 |
| Synthesis methods | 13a | Describe the processes used to decide which studies were eligible for each synthesis (e.g. tabulating the study intervention characteristics and comparing against the planned groups for each synthesis (item #5)). | Page 6 |
|  | 13b | Describe any methods required to prepare the data for presentation or synthesis, such as handling of missing summary statistics, or data conversions. | Page 7 |
|  | 13c | Describe any methods used to tabulate or visually display results of individual studies and syntheses. | Page 8 |
|  | 13d | Describe any methods used to synthesize results and provide a rationale for the choice(s). If meta-analysis was performed, describe the model(s), method(s) to identify the presence and extent of statistical heterogeneity, and software package(s) used. | As above |
|  | 13e | Describe any methods used to explore possible causes of heterogeneity among study results (e.g. subgroup analysis, meta-regression). | NA |
|  | 13f | Describe any sensitivity analyses conducted to assess robustness of the synthesized results. | NA |
| Reporting bias assessment | 14 | Describe any methods used to assess risk of bias due to missing results in a synthesis (arising from reporting biases). | Page 7 |
| Certainty assessment | 15 | Describe any methods used to assess certainty (or confidence) in the body of evidence for an outcome. | NA |
| **RESULTS** | | |  |
| Study selection | 16a | Describe the results of the search and selection process, from the number of records identified in the search to the number of studies included in the review, ideally using a flow diagram. | Page 8 |
|  | 16b | Cite studies that might appear to meet the inclusion criteria, but which were excluded, and explain why they were excluded. | Figure 3 PRISMA Flow chart |
| Study characteristics | 17 | Cite each included study and present its characteristics. | Supplementary tables 1-6 |
| Risk of bias in studies | 18 | Present assessments of risk of bias for each included study. | For manual and APM we used modified Downs-Black checklist  For AI studies, no tool exists- see body of text for explanation (bottom of page 8) |
| Results of individual studies | 19 | For all outcomes, present, for each study: (a) summary statistics for each group (where appropriate) and (b) an effect estimate and its precision (e.g. confidence/credible interval), ideally using structured tables or plots. | See Tables and supplementary tables |
| Results of syntheses | 20a | For each synthesis, briefly summarise the characteristics and risk of bias among contributing studies. | Pages 8-13 |
|  | 20b | Present results of all statistical syntheses conducted. If meta-analysis was done, present for each the summary estimate and its precision (e.g. confidence/credible interval) and measures of statistical heterogeneity. If comparing groups, describe the direction of the effect. | NA |
|  | 20c | Present results of all investigations of possible causes of heterogeneity among study results. | NA |
|  | 20d | Present results of all sensitivity analyses conducted to assess the robustness of the synthesized results. | NA |
| Reporting biases | 21 | Present assessments of risk of bias due to missing results (arising from reporting biases) for each synthesis assessed. | NA |
| Certainty of evidence | 22 | Present assessments of certainty (or confidence) in the body of evidence for each outcome assessed. | Level of evidence and recommendation in tables and supplementary table |
| **DISCUSSION** | | |  |
| Discussion | 23a | Provide a general interpretation of the results in the context of other evidence. | Page 14-20 |
|  | 23b | Discuss any limitations of the evidence included in the review. | Page 20-21 |
|  | 23c | Discuss any limitations of the review processes used. | Page 20-21 |
|  | 23d | Discuss implications of the results for practice, policy, and future research. | Page 14-21 |
| **OTHER INFORMATION** | | |  |
| Registration and protocol | 24a | Provide registration information for the review, including register name and registration number, or state that the review was not registered. | Page 2 acknowledgements |
|  | 24b | Indicate where the review protocol can be accessed, or state that a protocol was not prepared. | Prospero, page 2 |
|  | 24c | Describe and explain any amendments to information provided at registration or in the protocol. | NA |
| Support | 25 | Describe sources of financial or non-financial support for the review, and the role of the funders or sponsors in the review. | NA |
| Competing interests | 26 | Declare any competing interests of review authors. | Page 1 |
| Availability of data, code and other materials | 27 | Report which of the following are publicly available and where they can be found: template data collection forms; data extracted from included studies; data used for all analyses; analytic code; any other materials used in the review. | NA |

*From:*  Page MJ, McKenzie JE, Bossuyt PM, Boutron I, Hoffmann TC, Mulrow CD, et al. The PRISMA 2020 statement: an updated guideline for reporting systematic reviews. BMJ 2021;372:n71. doi: 10.1136/bmj.n71

For more information, visit: <http://www.prisma-statement.org/>

**References**

1. Khajeh E, Aminizadeh E, Dooghaie Moghadam A, Nikbakhsh R, Goncalves G, Carvalho C, et al. Outcomes of Robot-Assisted Surgery in Rectal Cancer Compared with Open and Laparoscopic Surgery. Vol. 15, Cancers. MDPI; 2023.

2. Hopkins MB, Geiger TM, Bethurum AJ, Ford MM, Muldoon RL, Beck DE, et al. Comparing pathologic outcomes for robotic versus laparoscopic Surgery in rectal cancer resection: a propensity adjusted analysis of 7616 patients. Surg Endosc. 2020 Jun 1;34(6):2613–22.

3. Markar SR, Karthikesalingam AP, Venkat-Ramen V, Kinross J, Ziprin P. Robotic vs. laparoscopic Roux-en-Y gastric bypass in morbidly obese patients: Systematic review and pooled analysis. Vol. 7, International Journal of Medical Robotics and Computer Assisted Surgery. John Wiley and Sons Ltd; 2011. p. 393–400.

4. Safiejko K, Tarkowski R, Koselak M, Juchimiuk M, Tarasik A, Pruc M, et al. Robotic-assisted vs. Standard laparoscopic surgery for rectal cancer resection: A systematic review and meta-analysis of 19,731 patients. Vol. 14, Cancers. MDPI; 2022.

5. Kamarajah SK, Bundred J, Marc O Saint, Jiao LR, Manas D, Abu Hilal M, et al. Robotic versus conventional laparoscopic pancreaticoduodenectomy a systematic review and meta-analysis. Vol. 46, European Journal of Surgical Oncology. W.B. Saunders Ltd; 2020. p. 6–14.

6. Curtis NJ, Dennison G, Brown CSB, Hewett PJ, Hanna GB, Stevenson ARL, et al. Clinical evaluation of intraoperative near misses in laparoscopic rectal cancer surgery. Ann Surg. 2021 Apr 1;273(4):778–84.

7. Collins JW, Dell’Oglio P, Hung AJ, Brook NR. The Importance of Technical and Non-technical Skills in Robotic Surgery Training [Figure presented]. Vol. 4, European Urology Focus. Elsevier B.V.; 2018. p. 674–6.

8. Vincent C, Neale G, Woloshynowych M. Adverse events in British hospitals: preliminary retrospective record review. BMJ. 2001 Mar 3;322(7285):517–9.

9. Collins JW, Levy J, Stefanidis D, Gallagher A, Coleman M, Cecil T, et al. Utilising the Delphi Process to Develop a Proficiency-based Progression Train-the-trainer Course for Robotic Surgery Training. Vol. 75, European Urology. Elsevier B.V.; 2019. p. 775–85.

10. ECRI. Top 10 Health Technology Hazards for 2015 [Internet]. 2014. Available from: www.ecri.org/2015hazards,

11. Raza SJ, Froghi S, Chowriappa A, Ahmed K, Field E, Stegemann AP, et al. Construct validation of the key components of fundamental skills of robotic surgery (FSRS) curriculum - A multi-institution prospective study. J Surg Educ. 2014;71(3):316–24.

12. Stegemann AP, Ahmed K, Syed JR, Rehman S, Ghani K, Autorino R, et al. Fundamental skills of robotic surgery: A multi-institutional randomized controlled trial for validation of a simulation-based curriculum. Urology. 2013 Apr;81(4):767–74.

13. Satava R, Smith R, Patel V, Advincula A, Aggarwal ; R, al Ansari ; A, et al. Fundamentals of Robotic Surgery: Outcomes Measures and Curriculum Development Principle Investigators. Society of Laproendoscopic Surgeons. 2012;

14. Goh AC, Aghazadeh MA, Mercado MA, Hung AJ, Pan MM, Desai MM, et al. Multi-institutional validation of fundamental inanimate robotic skills tasks. Journal of Urology. 2015 Dec 1;194(6):1751–6.

15. Satava RM, Stefanidis D, Levy JS, Smith R, Martin JR, Monfared S, et al. Proving the effectiveness of the fundamentals of robotic surgery (FRS) skills curriculum: A single-blinded, multispecialty, multi-institutional randomized control trial. Ann Surg. 2020 Aug 1;272(2):384–92.

16. S Schmiederer I, Torices-Dardon A, M Ferrari-Light D, Charbel Abboud E, Villani V, N Lau J, et al. Developing a Robotic General Surgery Training Curriculum: Identifying Key Elements Through a Delphi Process. J Surg Educ. 2021 Nov 1;78(6):e129–36.

17. Smith R, Patel V, Satava R. Fundamentals of robotic surgery: A course of basic robotic surgery skills based upon a 14-society consensus template of outcomes measures and curriculum development. International Journal of Medical Robotics and Computer Assisted Surgery. 2014 Sep 1;10(3):379–84.

18. Challacombe BM, Urol F, Ahmed MRCS K, Dasgupta P, Shamim Khan OBE M, Cross FRCS Urol W, et al. British Association of Urological Surgeons (BAUS) Robotic Surgery Curriculum- Guidelines for Training [Internet]. [cited 2021 Sep 29]. Available from: https://www.baus.org.uk/professionals/baus_business/publications/83/robotic_surgery_curriculum/

19. Veronesi G, Dorn P, Dunning J, Cardillo G, Schmid RA, Collins J, et al. Outcomes from the Delphi process of the Thoracic Robotic Curriculum Development Committee. European Journal of Cardio-thoracic Surgery. 2018 Jun 1;53(6):1173–9.

20. Szold A, Bergamaschi R, Broeders I, Dankelman J, Forgione A, Langø T, et al. European association of endoscopic surgeons (EAES) consensus statement on the use of robotics in general surgery. Surg Endosc. 2015 Jan 14;29(2):253–88.

21. Vanlander AE, Mazzone E, Collins JW, Mottrie AM, Rogiers XM, van der Poel HG, et al. Orsi Consensus Meeting on European Robotic Training (OCERT): Results from the First Multispecialty Consensus Meeting on Training in Robot-assisted Surgery. Vol. 78, European Urology. Elsevier B.V.; 2020. p. 713–6.

22. Gómez Ruiz M, Alfieri S, Becker T, Bergmann M, Boggi U, Collins J, et al. Expert consensus on a train-the-trainer curriculum for robotic colorectal surgery. Colorectal Disease. 2019;21(8):903–8.

23. Palagonia E, Mazzone E, De Naeyer G, D’Hondt F, Collins J, Wisz P, et al. The safety of urologic robotic surgery depends on the skills of the surgeon. World J Urol. 2020 Jun 1;38(6):1373–83.

24. Stefanidis D, Huffman EM, Collins JW, Martino MA, Satava RM, Levy JS. Expert Consensus Recommendations for Robotic Surgery Credentialing. Ann Surg. 2022 Jul 1;276(1):88–93.

25. Birkmeyer JD, Finks JF, O’Reilly A, Oerline M, Carlin AM, Nunn AR, et al. Surgical Skill and Complication Rates after Bariatric Surgery. New England Journal of Medicine. 2013 Oct 10;369(15):1434–42.

26. Hanna GB, Mackenzie H, Miskovic D, Ni M, Wyles S, Aylin P, et al. Laparoscopic Colorectal Surgery Outcomes Improved After National Training Program (LAPCO) for Specialists in England. Ann Surg. 2020 Oct 19;

27. Curtis NJ, Foster JD, Miskovic D, Brown CSB, Hewett PJ, Abbott S, et al. Association of Surgical Skill Assessment with Clinical Outcomes in Cancer Surgery. JAMA Surg. 2020 Jul 1;155(7):590–8.

28. Chen J, Cheng N, Cacciamani G, Oh P, Lin-Brande M, Remulla D, et al. Objective Assessment of Robotic Surgical Technical Skill: A Systematic Review. Journal of Urology. 2019 Mar 1;201(3):461–9.

29. Vaidya A, Aydin A, Ridgley J, Raison N, Dasgupta P, Ahmed K. Current Status of Technical Skills Assessment Tools in Surgery: A Systematic Review. Vol. 246, Journal of Surgical Research. Academic Press Inc.; 2020. p. 342–78.

30. Levin M, McKechnie T, Khalid S, Grantcharov TP, Goldenberg M. Automated Methods of Technical Skill Assessment in Surgery: A Systematic Review. Vol. 76, Journal of Surgical Education. Elsevier Inc.; 2019. p. 1629–39.

31. Lam K, Chen J, Wang Z, Iqbal FM, Darzi A, Lo B, et al. Machine learning for technical skill assessment in surgery: a systematic review. NPJ Digit Med [Internet]. 2022 Dec 3;5(1):24. Available from: https://www.nature.com/articles/s41746-022-00566-0

32. Kutana S, Bitner DP, Addison P, Chung PJ, Talamini MA, Filicori F. Objective assessment of robotic surgical skills: review of literature and future directions. Surg Endosc [Internet]. 2021 [cited 2022 Mar 30];1:3. Available from: https://doi.org/10.1007/s00464-022-09134-9

33. Chen AB, Liang S, Nguyen JH, Liu Y, Hung AJ. Machine learning analyses of automated performance metrics during granular sub-stitch phases predict surgeon experience HHS Public Access. Surgery. 2021;169(5):1245–9.

34. Hung AJ, Chen J, Gill IS. Automated performance metrics and machine learning algorithms to measure surgeon performance and anticipate clinical outcomes in robotic surgery. Vol. 153, JAMA Surgery. American Medical Association; 2018. p. 770–1.

35. Ghodoussipour S, Reddy SS, Ma R, Huang D, Nguyen J, Hung AJ. An Objective Assessment of Performance during Robotic Partial Nephrectomy: Validation and Correlation of Automated Performance Metrics with Intraoperative Outcomes. J Urol. 2021 May 1;205(5):1294–302.

36. Hung AJ, Ma R, Cen S, Nguyen JH, Lei X, Wagner C. Surgeon Automated Performance Metrics as Predictors of Early Urinary Continence Recovery After Robotic Radical Prostatectomy—A Prospective Bi-institutional Study. Eur Urol Open Sci. 2021 May 1;27:65–72.

37. Chen J, Chu T, Ghodoussipour S, Bowman S, Patel H, King K, et al. Effect of surgeon experience and bony pelvic dimensions on surgical performance and patient outcomes in robot-assisted radical prostatectomy. BJU Int. 2019 Nov 1;124(5):828–35.

38. Kumar A, Smith R, Patel VR. Current status of robotic simulators in acquisition of robotic surgical skills. Vol. 25, Current Opinion in Urology. Lippincott Williams and Wilkins; 2015. p. 168–74.

39. Moglia A, Ferrari V, Morelli L, Ferrari M, Mosca F, Cuschieri A. A Systematic Review of Virtual Reality Simulators for Robot-assisted Surgery. Vol. 69, European Urology. Elsevier B.V.; 2016. p. 1065–80.

40. Julian D, Tanaka A, Mattingly P, Truong M, Perez M, Smith R. A comparative analysis and guide to virtual reality robotic surgical simulators. International Journal of Medical Robotics and Computer Assisted Surgery. 2018 Feb 1;14(1).

41. Moher D, Liberati A, Tetzlaff J, Altman DG. Preferred reporting items for systematic reviews and meta-analyses: The PRISMA statement. Vol. 339, BMJ (Online). 2009. p. 332–6.

42. Goldenberg MG, Lee JY, Kwong JCC, Grantcharov TP, Costello A. Implementing assessments of robot-assisted technical skill in urological education: a systematic review and synthesis of the validity evidence. Vol. 122, BJU International. Blackwell Publishing Ltd; 2018. p. 501–19.

43. Messick S. Foundations of Validity: Meaning and Consequences in Psychological Assessment. 1993.

44. Carter FJ, Schijven MP, Aggarwal R, Grantcharov T, Francis NK, Hanna GB, et al. Consensus guidelines for validation of virtual reality surgical simulators. Surgical Endoscopy and Other Interventional Techniques [Internet]. 2005 [cited 2022 Oct 4]; Available from: http://www.eaes-eur.org

45. Downs SH, Black N. The feasibility of creating a checklist for the assessment of the methodological quality both of randomised and non-randomised studies of health care interventions. J Epidemiol Community Health (1978). 1998;52(6):377–84.

46. Cook DA, Reed DA. Appraising the Quality of Medical Education Research Methods: The Medical Education Research Study Quality Instrument and the Newcastle-Ottawa Scale-Education. Academic Medicine. 2015 Aug 31;90(8):1067–76.

47. Holst D, Kowalewski TM, White LW, Brand TC, Harper JD, Sorenson MD, et al. Crowd-Sourced Assessment of Technical Skills: An Adjunct to Urology Resident Surgical Simulation Training. J Endourol. 2015 May;29(5).

48. Holst D, Kowalewski TM, White LW, Brand TC, Harper JD, Sorensen MD, et al. Crowd-sourced assessment of technical skills: Differentiating animate surgical skill through the wisdom of crowds. J Endourol. 2015 Oct 1;29(10):1183–8.

49. Powers MK, Boonjindasup A, Pinsky M, Dorsey P, Maddox M, Su LM, et al. Crowdsourcing Assessment of Surgeon Dissection of Renal Artery and Vein during Robotic Partial Nephrectomy: A Novel Approach for Quantitative Assessment of Surgical Performance. J Endourol. 2016 Apr 1;30(4):447–52.

50. Ghani KR, Miller DC, Linsell S, Brachulis A, Lane B, Sarle R, et al. Measuring to improve: Peer and crowd-sourced assessments of technical skill with robot-assisted radical prostatectomy. Eur Urol. 2016 Apr 1;69(4):547–50.

51. Vernez SL, Huynh V, Osann K, Okhunov Z, Landman J, Clayman R V. C-SATS: Assessing surgical skills among urology residency applicants. J Endourol. 2017 Apr 1;31:S95–100.

52. Ghazi A, Melnyk R, Hung AJ, Collins J, Ertefaie A, Saba P, et al. Multi-institutional validation of a perfused robot-assisted partial nephrectomy procedural simulation platform utilizing clinically relevant objective metrics of simulators (CROMS). BJU Int. 2021 Jun 1;127(6):645–53.

53. Ghani KR, Comstock B, Miller DC, Dunn RL, Kim T, Linsell S, et al. Technical Skill Assessment of Surgeons Performing Robotic-Assisted Radical Prostatectomy: Relationship between Crowdsourced Review and Patient Outcomes. Journal of Urology [Internet]. 2017 Apr;197(4S). Available from: http://www.jurology.com/doi/10.1016/j.juro.2017.02.3221

54. Tunitsky E, Murphy A, Barber MD, Simmons M, Jelovsek JE. Development and validation of a ureteral anastomosis simulation model for surgical training. Female Pelvic Med Reconstr Surg. 2013;19(6):346–51.

55. Siddiqui NY, Tarr ME, Geller EJ, Advincula AP, Galloway ML, Green IC, et al. Establishing Benchmarks For Minimum Competence With Dry Lab Robotic Surgery Drills. J Minim Invasive Gynecol. 2016 May 1;23(4):633–8.

56. Hussein AA, Sexton KJ, May PR, Meng M V., Hosseini A, Eun DD, et al. Development and validation of surgical training tool: cystectomy assessment and surgical evaluation (CASE) for robot-assisted radical cystectomy for men. Surg Endosc. 2018 Nov 1;32(11):4458–64.

57. Hussein AA, Abaza R, Rogers C, Boris R, Porter J, Allaf M, et al. Development and validation of an objective scoring tool for minimally invasive partial nephrectomy: Scoring for Partial Nephrectomy (SPaN). Journal of Urology. 2018 Apr;199(4S).

58. Stefanidis D, Anderson-Montoya B, Higgins R V., Pimentel ME, Rowland P, Scarborough MO, et al. Developing a coaching mechanism for practicing surgeons. Surgery . 2016 Sep 1;160(3):536–45.

59. Petz W, Spinoglio G, Choi GS, Parvaiz A, Santiago C, Marecik S, et al. Structured training and competence assessment in colorectal robotic surgery. Results of a consensus experts round table. International Journal of Medical Robotics and Computer Assisted Surgery. 2016 Dec 1;12(4):634–41.

60. Panteleimonitis S, Popeskou S, Aradaib M, Harper M, Ahmed J, Ahmad M, et al. Implementation of robotic rectal surgery training programme: importance of standardisation and structured training. Langenbecks Arch Surg. 2018 Sep 1;403(6):749–60.

61. Eddahchouri Y, van Workum F, van den Wildenberg FJH, van Berge Henegouwen MI, Polat F, van Goor H, et al. European consensus on essential steps of Minimally Invasive Ivor Lewis and McKeown Esophagectomy through Delphi methodology. Surg Endosc. 2022 Jan 1;36(1):446–60.

62. Sobel RH, Blanco R, Ha PK, Califano JA, Kumar R, Richmon JD. Implementation of a comprehensive competency-based transoral robotic surgery training curriculum with ex vivo dissection models. Head Neck. 2016 Oct 1;38(10):1553–63.

63. Willuth E, Hardon SF, Lang F, Haney CM, Felinska EA, Kowalewski KF, et al. Robotic-assisted cholecystectomy is superior to laparoscopic cholecystectomy in the initial training for surgical novices in an ex vivo porcine model: a randomized crossover study. Surg Endosc. 2022 Feb 1;36(2):1064–79.

64. Ghani K, Guru K, Aly A, Lane B, Sarle R, Linsell S, et al. Variation in technical skill of surgeons performing robot-assisted prostatectomy. Journal of Urology. 2016 Apr;195(4S).

65. Frederick PJ, Szender JB, Hussein AA, Kesterson JP, Shelton JA, Anderson TL, et al. Surgical Competency for Robot-Assisted Hysterectomy: Development and Validation of a Robotic Hysterectomy Assessment Score (RHAS). J Minim Invasive Gynecol. 2017 Jan 1;24(1):55–61.

66. Hussein AA, Ghani KR, Peabody J, Sarle R, Abaza R, Eun D, et al. Development and Validation of an Objective Scoring Tool for Robot-Assisted Radical Prostatectomy: Prostatectomy Assessment and Competency Evaluation. Journal of Urology. 2017 May 1;197(5):1237–44.

67. Beulens AJW, Brinkman · W M, Van Der Poel · H G, Vis · A N, Van Basten · J P, Meijer · R P, et al. Linking surgical skills to postoperative outcomes: a Delphi study on the robot-assisted radical prostatectomy. J Robot Surg [Internet]. 2019;13:675–87. Available from: https://doi.org/10.1007/s11701-018-00916-9

68. Lovegrove C, Bruce E, Raison N, Challacombe B, Novara G, Mottrie A, et al. Development and content validation of a training and assessment tool for RAPN. Journal of Urology. 2017 Apr;197(4S).

69. Lovegrove C, Ahmed K, Novara G, Guru K, Mottrie A, Challacombe B, et al. Modular Training for Robot-Assisted Radical Prostatectomy: Where to Begin? J Surg Educ [Internet]. 2017 May 1 [cited 2022 Mar 10];74(3):486–94. Available from: http://dx.doi.org/10.1016/j.jsurg.2016.11.002

70. Lovegrove C, Novara G, Mottrie A, Guru KA, Brown M, Challacombe B, et al. Structured and Modular Training Pathway for Robot-assisted Radical Prostatectomy (RARP): Validation of the RARP Assessment Score and Learning Curve Assessment. Eur Urol. 2016 Mar 1;69(3):526–35.

71. Chow AK, Wong R, Monda S, Bhatt R, Sands KG, Vetter J, et al. Ex Vivo Porcine Model for Robot-Assisted Partial Nephrectomy Simulation at a High-Volume Tertiary Center: Resident Perception and Validation Assessment Using the Global Evaluative Assessment of Robotic Skills Tool. J Endourol. 2021 Jun 1;35(6):878–84.

72. Davis JW, Kamat A, Munsell M, Pettaway C, Pisters L, Matin S. Initial experience of teaching robot-assisted radical prostatectomy to surgeons-in-training: Can training be evaluated and standardized? BJU Int. 2010 Apr;105(8):1148–54.

73. Volpe A, Ahmed K, Dasgupta P, Ficarra V, Novara G, Van Der Poel H, et al. Pilot Validation Study of the European Association of Urology Robotic Training Curriculum. Eur Urol. 2015 Aug 1;68(2):292–9.

74. Iqbal U, Jing Z, Ahmed Y, Elsayed AS, Rogers C, Boris R, et al. Development and Validation of an Objective Scoring Tool for Robot-Assisted Partial Nephrectomy: Scoring for Partial Nephrectomy. J Endourol. 2022 May 1;36(5):647–53.

75. Tou S, Gómez Ruiz M, Gallagher AG, Matzel KE, Amin S, Bianchi P, et al. European expert consensus on a structured approach to training robotic-assisted low anterior resection using performance metrics. Colorectal Disease. 2020;

76. Mottrie A, Mazzone E, Wiklund P, Graefen M, Collins JW, De Groote R, et al. Objective assessment of intraoperative skills for robot-assisted radical prostatectomy (RARP): results from the ERUS Scientific and Educational Working Groups Metrics Initiative. BJU Int [Internet]. 2021;128:103–11. Available from: www.bjui.org

77. Gómez Ruiz M, Tou S, Gallagher AG, Cagigas Fernández C, Cristobal Poch L, Matzel KE. Intraoperative robotic-assisted low anterior rectal resection performance assessment using procedure-specific binary metrics and a global rating scale. BJS Open. 2022 May 2;6(3).

78. Khan H, Kozlowski JD, Hussein AA, Sharif M, Ahmed Y, May P, et al. Use of Robotic Anastomosis Competency Evaluation (RACE) tool for assessment of surgical competency during urethrovesical anastomosis. Canadian Urological Association Journal. 2019 Jan 1;13(1):E10–6.

79. Hussein AA, Hinata N, Dibaj S, May PR, Kozlowski JD, Abol-Enein H, et al. Development, validation and clinical application of Pelvic Lymphadenectomy Assessment and Completion Evaluation: intraoperative assessment of lymph node dissection after robot-assisted radical cystectomy for bladder cancer. BJU Int. 2017 Jun 1;119(6):879–84.

80. Hung AJ, Bottyan T, Clifford TG, Serang S, Nakhoda ZK, Shah SH, et al. Structured learning for robotic surgery utilizing a proficiency score: a pilot study. World J Urol. 2017;35:27–34.

81. Raza SJ, Field E, Jay C, Eun D, Fumo M, Hu JC, et al. Surgical competency for urethrovesical anastomosis during robot-assisted radical prostatectomy: Development and validation of the robotic anastomosis competency evaluation. Urology. 2015 Jan 1;85(1):27–32.

82. Chowriappa A, Raza SJ, Fazili A, Field E, Malito C, Samarasekera D, et al. Augmented-reality-based skills training for robot-assisted urethrovesical anastomosis: A multi-institutional randomised controlled trial. BJU Int. 2015 Feb 1;115(2):336–45.

83. Hoogenes J, Wong N, Al-Harbi B, Kim KS, Vij S, Bolognone E, et al. A Randomized Comparison of 2 Robotic Virtual Reality Simulators and Evaluation of Trainees’ Skills Transfer to a Simulated Robotic Urethrovesical Anastomosis Task. Urology. 2018 Jan;111:110–5.

84. Witthaus MW, Farooq S, Melnyk R, Campbell T, Saba P, Mathews E, et al. Incorporation and validation of clinically relevant performance metrics of simulation (CRPMS) into a novel full-immersion simulation platform for nerve-sparing robot-assisted radical prostatectomy (NS-RARP) utilizing three-dimensional printing and hydrogel casting technology Professional Innovation Introduction. BJU International © [Internet]. 2020;125:322–32. Available from: www.bjui.org

85. Schmidt MW, Haney CM, Kowalewski KF, Bintintan V V., Abu Hilal M, Arezzo A, et al. Development and validity evidence of an objective structured assessment of technical skills score for minimally invasive linear-stapled, hand-sewn intestinal anastomoses: the A-OSATS score. Surg Endosc. 2022 Jun 1;36(6):4529–41.

86. Hogg ME, Zenati M, Novak S, Chen Y, Jun Y, Steve J, et al. Grading of surgeon technical performance predicts postoperative pancreatic fistula for pancreaticoduodenectomy independent of patient-related variables. In: Annals of Surgery. Lippincott Williams and Wilkins; 2016. p. 482–9.

87. Moloney K, Janda M, Frumovitz M, Leitao M, Abu-Rustum NR, Rossi E, et al. Development of a surgical competency assessment tool for sentinel lymph node dissection by minimally invasive surgery for endometrial cancer. International Journal of Gynecological Cancer. 2021 May 1;31(5):647–55.

88. Willems JIP, Shin AM, Shin DM, Bishop AT, Shin AY. A comparison of robotically assisted microsurgery versus manual microsurgery in challenging situations. Plast Reconstr Surg. 2016;137(4):1317–24.

89. Suh I, Mukherjee M, Oleynikov D, Siu KC. Training program for fundamental surgical skill in robotic laparoscopic surgery. International Journal of Medical Robotics and Computer Assisted Surgery. 2011 Sep;7(3):327–33.

90. Egi H, Hattori M, Tokunaga M, Suzuki T, Kawaguchi K, Sawada H, et al. Face, Content and Concurrent Validity of the Mimic ® dV-Trainer for Robot-Assisted Endoscopic Surgery: A Prospective Study. Eur Surg Res [Internet]. 2013 [cited 2022 Aug 18];50:292–300. Available from: www.karger.com/esrwww.karger.com/esr

91. Møller SG, Dohrn N, Brisling SK, Larsen JCR, Klein M. Laparoscopic Versus Robotic-assisted Suturing Performance Among Novice Surgeons: A Blinded, Cross-Over Study. Surgery, Laparoscopy, Endoscopy and Percutaneous Techniques [Internet]. 2020; Available from: www.surgical-laparoscopy.com

92. Vaccaro CM, Crisp CC, Fellner AN, Jackson C, Kleeman SD, Pavelka J. Robotic virtual reality simulation plus standard robotic orientation versus standard robotic orientation alone: A randomized controlled trial. In: Female Pelvic Medicine and Reconstructive Surgery. Lippincott Williams and Wilkins; 2013. p. 266–70.

93. Puliatti S, Mazzone E, Amato M, De Groote R, Mottrie A, Gallagher AG. Development and validation of the objective assessment of robotic suturing and knot tying skills for chicken anastomotic model. Surg Endosc. 2021 Aug 1;35(8):4285–94.

94. Chang L, Satava RM, Pellegrini CA, Sinanan MN. Robotic surgery Identifying the learning curve through objective measurement of skill. Surg Endosc. 2003;

95. Singh H, Modi HN, Ranjan S, Dilley JWR, Airantzis D, Yang GZ, et al. Robotic Surgery Improves Technical Performance and Enhances Prefrontal Activation During High Temporal Demand. Ann Biomed Eng [Internet]. 2018;1621–36. Available from: https://doi.org/10.1007/s10439-018-2049-z

96. Vanstrum EB, Ma R, Maya-Silva J, Sanford D, Nguyen JH, Lei X, et al. Development and Validation of an Objective Scoring Tool to Evaluate Surgical Dissection: Dissection Assessment for Robotic Technique (DART). Urol Pract. 2021 Sep;8(5):596–604.

97. Puliatti S, Amato M, Mazzone E, Rosiello G, De Groote R, Piazza P, et al. Development and validation of the metric-based assessment of a robotic vessel dissection, vessel loop positioning, clip applying and bipolar coagulation task on an avian model. J Robot Surg. 2022 Jun 1;16(3):677–85.

98. Menhadji A, Abdelshehid C, Osann K, Alipanah R, Lusch A, Graversen J, et al. Tracking and assessment of technical skills acquisition among urology residents for open, laparoscopic, and robotic skills over 4 years: Is there a trend? J Endourol. 2013 Jun 1;27(6):783–8.

99. Hung AJ, Shah SH, Dalag L, Shin D, Gill IS. Development and Validation of a Novel Robotic Procedure Specific Simulation Platform: Partial Nephrectomy. Journal of Urology. 2015;194(2):520–6.

100. Balasundaram I, Aggarwal R, Darzi A. Short-phase training on a virtual reality simulator improves technical performance in tele-robotic surgery. International Journal of Medical Robotics and Computer Assisted Surgery. 2008;4(2):139–45.

101. Van Der Meijden O, Schijven M, Broeders I, Der Meijden V. The SEP ‘Robot’^TM^: A Valid Virtual Reality Robotic Simulator for the Da Vinci Surgical System? Surgical Technology International XIX - Surgical Overview. 2010;51–8.

102. Ebbing J, Wiklund PN, Akre O, Carlsson S, Olsson MJ, Höijer J, et al. Development and validation of non-guided bladder-neck and neurovascular-bundle dissection modules of the RobotiX-Mentor® full-procedure robotic-assisted radical prostatectomy virtual reality simulation. International Journal of Medical Robotics and Computer Assisted Surgery. 2021 Apr 1;17(2).

103. Olsen RG, Bjerrum F, Konge L, Jepsen JV, Azawi NH, Bube SH. Validation of a Novel Simulation-Based Test in Robot-Assisted Radical Prostatectomy. J Endourol. 2021 Aug 1;35(8):1265–72.

104. Turner TB, Kim KH. Mapping the robotic hysterectomy learning curve and re-establishing surgical training metrics. J Gynecol Oncol. 2021;32(4).

105. Gomez ED, Aggarwal R, McMahan W, Bark K, Kuchenbecker KJ. Objective assessment of robotic surgical skill using instrument contact vibrations. Surg Endosc. 2016 Apr 1;30(4):1419–31.

106. Baghdadi A, Hussein AA, Ahmed Y, Cavuoto LA, Khurshid ·, Guru A. A computer vision technique for automated assessment of surgical performance using surgeons’ console-feed videos. Int J Comput Assist Radiol Surg [Internet]. 2019;14:697–707. Available from: https://doi.org/10.1007/s11548-018-1881-9

107. Dubin AK, Julian D, Tanaka A, Mattingly P, Smith · Roger. A model for predicting the GEARS score from virtual reality surgical simulator metrics. Surg Endosc [Internet]. 2018;32:3576–81. Available from: https://doi.org/10.1007/s00464-018-6082-7

108. Lavanchy JL, Zindel J, Kirtac K, Twick I, Hosgor E, Candinas D, et al. Automation of surgical skill assessment using a three-stage machine learning algorithm. Sci Rep. 2021 Dec 1;11(1).

109. Ahmidi N, Tao L, Sefati S, Gao Y, Lea C, Haro BB, et al. A Dataset and Benchmarks for Segmentation and Recognition of Gestures in Robotic Surgery. IEEE Trans Biomed Eng. 2017 Sep 1;64(9):2025–41.

110. Liu D, Li Q, Jiang T, Wang Y, Miao R, Shan F, et al. Towards Unified Surgical Skill Assessment. In: Proceedings of the IEEE Computer Society Conference on Computer Vision and Pattern Recognition. 2021.

111. Li Z, Gu L, Wang W, Nakamura R, Sato Y. Surgical Skill Assessment via Video Semantic Aggregation. In: Lecture Notes in Computer Science (including subseries Lecture Notes in Artificial Intelligence and Lecture Notes in Bioinformatics). 2022.

112. Law H, Ghani K, Deng J. Surgeon Technical Skill Assessment using Computer Vision based Analysis. Proc Mach Learn Res. 2017;

113. Hung AJ, Chen J, Che Z, Nilanon T, Jarc A, Titus M, et al. Utilizing Machine Learning and Automated Performance Metrics to Evaluate Robot-Assisted Radical Prostatectomy Performance and Predict Outcomes. J Endourol. 2018 May 1;32(5):438–44.

114. Ma R, Ramaswamy A, Xu J, Trinh L, Kiyasseh D, Chu TN, et al. Surgical gestures as a method to quantify surgical performance and predict patient outcomes. NPJ Digit Med. 2022 Dec 1;5(1).

115. Hung AJ, Chen J, Ghodoussipour S, Oh PJ, Liu Z, Nguyen J, et al. A deep-learning model using automated performance metrics and clinical features to predict urinary continence recovery after robot-assisted radical prostatectomy. BJU Int. 2019;124(3):487–95.

116. Liu D, Jiang T, Wang Y, Miao R, Shan F, Li Z. Surgical Skill Assessment on In-Vivo Clinical Data via the Clearness of Operating Field. In: Lecture Notes in Computer Science (including subseries Lecture Notes in Artificial Intelligence and Lecture Notes in Bioinformatics). 2019.

117. Lee D, Won Yu H, Kwon H, Kong HJ, Eun Lee K, Chan Kim H. Evaluation of Surgical Skills during Robotic Surgery by Deep Learning-Based Multiple Surgical Instrument Tracking in Training and Actual Operations. J Clin Med [Internet]. 2020; Available from: www.mdpi.com/journal/jcm

118. Fawaz HI, Forestier G, Weber J, Lhassane Idoumghar ·, Muller PA. Accurate and interpretable evaluation of surgical skills from kinematic data using fully convolutional neural networks. Int J Comput Assist Radiol Surg [Internet]. 2019;14:1611–7. Available from: https://doi.org/10.1007/s11548-019-02039-4

119. Benmansour M, Malti A, Jannin P. Deep neural network architecture for automated soft surgical skills evaluation using objective structured assessment of technical skills criteria. Int J Comput Assist Radiol Surg. 2023 Jan 25;

120. Zia A, Essa I. Automated surgical skill assessment in RMIS training. Int J Comput Assist Radiol Surg [Internet]. 2018;13:731–9. Available from: https://doi.org/10.1007/s11548-018-1735-5

121. Doughty H, Damen D, Mayol-Cuevas W. Who’s Better? Who’s Best? Pairwise Deep Ranking for Skill Determination. In: Proceedings of the IEEE Computer Society Conference on Computer Vision and Pattern Recognition. 2018.

122. Ershad M, Rege R, Majewicz Fey A. Automatic and near real-time stylistic behavior assessment in robotic surgery. Int J Comput Assist Radiol Surg. 2019 Apr 1;14(4):635–43.

123. Ma R, Vanstrum EB, Lee R, Chen J, Hung AJ. Machine learning in the optimization of robotics in the operative field. Vol. 30, Current opinion in urology. NLM (Medline); 2020. p. 808–16.

124. Wu C, Cha J, Sulek J, Sundaram CP, Wachs J, Proctor RW, et al. Sensor-based indicators of performance changes between sessions during robotic surgery training. Appl Ergon. 2021 Jan 1;90.

125. Ershad M, Rege R, Fey AM. Automatic Surgical Skill Rating Using Stylistic Behavior Components. IEEE Xplore. 2018;

126. Ershad M, Rege R, Majewicz Fey A. Meaningful Assessment of Robotic Surgical Style using the Wisdom of Crowds. Int J Comput Assist Radiol Surg [Internet]. 2018;13:1037–48. Available from: https://doi.org/10.1007/s11548-018-1738-2

127. Wang T, Wang Y, Li M. Towards Accurate and Interpretable Surgical Skill Assessment: A Video-Based Method Incorporating Recognized Surgical Gestures and Skill Levels. In: Lecture Notes in Computer Science (including subseries Lecture Notes in Artificial Intelligence and Lecture Notes in Bioinformatics). 2020.

128. Anastasiou D, Jin Y, Stoyanov D, Mazomenos E. Keep Your Eye on the Best: Contrastive Regression Transformer for Skill Assessment in Robotic Surgery. IEEE Robot Autom Lett. 2023;

129. Oğul BB, Gilgien MF, Şahin PD. Ranking Robot-Assisted Surgery Skills Using Kinematic Sensors. Chatzigiannakis I, De Ruyter B, Mavrommati I, editors. Ambient Intelligence [Internet]. 2019;11912. Available from: http://link.springer.com/10.1007/978-3-030-34255-5

130. Soleymani A, Sadat Asl AA, Yeganejou M, Dick S, Tavakoli M, Li X. Surgical Skill Evaluation from Robot-Assisted Surgery Recordings. In: 2021 International Symposium on Medical Robotics, ISMR 2021. Institute of Electrical and Electronics Engineers Inc.; 2021.

131. Parmar P, Morris BT. Learning to Score Olympic Events. In: IEEE Computer Society Conference on Computer Vision and Pattern Recognition Workshops. 2017.

132. Xiang X, Tian Y, Reiter A, Hager GD, Tran TD. S3D: Stacking Segmental P3D for Action Quality Assessment. In: Proceedings - International Conference on Image Processing, ICIP. 2018.

133. Fawaz H, Forestier G, Weber J, Idoumghar L, Muller PA. Evaluating Surgical Skills from Kinematic Data Using Convolutional Neural Networks. In: Lecture Notes in Computer Science (including subseries Lecture Notes in Artificial Intelligence and Lecture Notes in Bioinformatics). 2018.

134. Wang Z, Majewicz Fey A. Deep learning with convolutional neural network for objective skill evaluation in robot-assisted surgery. Int J Comput Assist Radiol Surg. 2018 Dec 1;13(12):1959–70.

135. Anh NX, Nataraja RM, Chauhan S. Towards near real-time assessment of surgical skills: A comparison of feature extraction techniques. Comput Methods Programs Biomed [Internet]. 2020 [cited 2022 Feb 15];187:105234. Available from: https://doi.org/10.1016/j.cmpb.2019.105234

136. Nguyen XA, Ljuhar D, Pacilli M, Nataraja RM, Chauhan S. Surgical skill levels: Classification and analysis using deep neural network model and motion signals. Comput Methods Programs Biomed. 2019 Aug 1;177:1–8.

137. Wang Z, Fey AM. Improving Surgical Skill Assessment and Task Recognition in Robot-assisted Surgery with Deep Neural Networks. IEEE Xplore. 2018;

138. Kiyasseh D, Ma R, Haque TF, Miles BJ, Wagner C, Donoho DA, et al. A vision transformer for decoding surgeon activity from surgical videos. Nat Biomed Eng [Internet]. 2023 Mar 30; Available from: http://www.ncbi.nlm.nih.gov/pubmed/36997732

139. Kiyasseh D, Laca J, Haque TF, Miles BJ, Wagner C, Donoho DA, et al. A multi-institutional study using artificial intelligence to provide reliable and fair feedback to surgeons. Communications Medicine [Internet]. 2023 Mar 30;3(1):42. Available from: https://www.nature.com/articles/s43856-023-00263-3

140. Kiyasseh D, Laca J, Haque TF, Otiato M, Miles BJ, Wagner C, et al. Human visual explanations mitigate bias in AI-based assessment of surgeon skills. NPJ Digit Med [Internet]. 2023 Mar 30;6(1):54. Available from: https://www.nature.com/articles/s41746-023-00766-2

141. Collins JW, Marcus HJ, Ghazi A, Sridhar A, Hashimoto D, Hager G, et al. Ethical implications of AI in robotic surgical training: A Delphi consensus statement. Eur Urol Focus. 2022 Mar;8(2):613–22.

142. World Health Organization. World Health Organization . 2023 [cited 2023 May 26]. WHO calls for safe and ethical AI for health. Available from: https://www.who.int/news/item/16-05-2023-who-calls-for-safe-and-ethical-ai-for-health

143. Scott SI, Dalsgaard T, Jepsen JV, von Buchwald C, Andersen SAW. Design and validation of a cross-specialty simulation-based training course in basic robotic surgical skills. International Journal of Medical Robotics and Computer Assisted Surgery. 2020 Oct 1;16(5):1–10.

144. Cook DA, Hatala R. Validation of educational assessments: a primer for simulation and beyond. Advances in Simulation. 2016 Jan;1(1).

145. Goh AC, Goldfarb DW, Sander JC, Miles BJ, Dunkin BJ. Global evaluative assessment of robotic skills: Validation of a clinical assessment tool to measure robotic surgical skills. Journal of Urology. 2012 Jan;187(1):247–52.

146. Aghazadeh MA, Mercado MA, Pan MM, Miles BJ, Goh AC. Performance of robotic simulated skills tasks is positively associated with clinical robotic surgical performance. BJU Int. 2016 Sep 1;118(3):475–81.

147. von Rundstedt FC, Aghazadeh MA, Scovell J, Slawin J, Armstrong J, Silay S, et al. Validation of a Simulation-training Model for Robotic Intracorporeal Bowel Anastomosis Using a Step-by-step Technique. Urology. 2018 Oct 1;120:125–30.

148. Aghazadeh MA, Jayaratna IS, Hung AJ, Pan MM, Desai MM, Gill IS, et al. External validation of Global Evaluative Assessment of Robotic Skills (GEARS). Surg Endosc. 2015 Nov 1;29(11):3261–6.

149. Whitehurst S V., Lockrow EG, Lendvay TS, Propst AM, Dunlow SG, Rosemeyer CJ, et al. Comparison of Two Simulation Systems to Support Robotic-Assisted Surgical Training: A Pilot Study (Swine Model). J Minim Invasive Gynecol. 2015 Mar 1;22(3):483–8.

150. Sánchez R, Rodríguez O, Rosciano J, Vegas L, Bond V, Rojas A, et al. Robotic surgery training: construct validity of Global Evaluative Assessment of Robotic Skills (GEARS). J Robot Surg. 2016 Sep 1;10(3):227–31.

151. Bur AM, Gomez ED, Newman JG, Weinstein GS, O’Malley BW, Rassekh CH, et al. Evaluation of high-fidelity simulation as a training tool in transoral robotic surgery. Laryngoscope. 2017 Dec 1;127(12):2790–5.

152. Brown JD, O Brien CE, Leung SC, Dumon KR, Lee DI, Kuchenbecker KJ. Using Contact Forces and Robot Arm Accelerations to Automatically Rate Surgeon Skill at Peg Transfer. IEEE Trans Biomed Eng. 2017 Sep 1;64(9):2263–75.

153. Vargas M V., Moawad G, Denny K, Happ L, Misa NY, Margulies S, et al. Transferability of Virtual Reality, Simulation-Based, Robotic Suturing Skills to a Live Porcine Model in Novice Surgeons: A Single-Blind Randomized Controlled Trial. In: Journal of Minimally Invasive Gynecology. Elsevier B.V.; 2017. p. 420–5.

154. Oh PJ, Chen J, Hatcher D, Djaladat H, Hung AJ. Crowdsourced versus expert evaluations of the vesico-urethral anastomosis in the robotic radical prostatectomy: is one superior at discriminating differences in automated performance metrics? J Robot Surg. 2018 Dec 1;12(4):705–11.

155. Raison N, Gavazzi A, Abe T, Ahmed K, Dasgupta P. Virtually Competent: A Comparative Analysis of Virtual Reality and Dry-Lab Robotic Simulation Training. J Endourol [Internet]. 2020 [cited 2022 Aug 23];34:379–84. Available from: www.liebertpub.com

156. Fukuoka K, Teishima J, Inoue S, Hayashi T, Matsubara A. The influence of reviewer’s occupation on the skill assessment of urethrovesical anastomosis in robot-assisted radical prostatectomy. Asian J Endosc Surg. 2021 Jul 1;14(3):451–7.

157. Hung AJ, Chen J, Jarc A, Hatcher D, Djaladat H, Gill IS. Development and Validation of Objective Performance Metrics for Robot-Assisted Radical Prostatectomy: A Pilot Study. Journal of Urology. 2018 Jan;199(1):296–304.

158. Yu N, Saadat H, Finelli A, Lee JY, Singal RK, Grantcharov TP, et al. Quantifying the “Assistant Effect” in Robotic-Assisted Radical Prostatectomy (RARP): Measures of Technical Performance. Journal of Surgical Research. 2021 Apr 1;260:307–14.

159. Kelly JD, Kowalewski TM, Brand T, French A, Nash M, Meryman L, et al. Virtual Reality Warm-up Before Robot-assisted Surgery: A Randomized Controlled Trial. Journal of Surgical Research. 2021 Aug 1;264:107–16.

160. Kiely DJ, Gotlieb WH, Lau S, Zeng X, Samouelian V, Agnihotram •, et al. Virtual reality robotic surgery simulation curriculum to teach robotic suturing: a randomized controlled trial. J Robot Surg [Internet]. 2015;9:179–86. Available from: www.clinicaltrials.gov.

161. Guni A, Raison N, Challacombe · Ben, Khan S, Prokar Dasgupta ·, Ahmed K. Development of a technical checklist for the assessment of suturing in robotic surgery. Surg Endosc [Internet]. 2018;32:4402–7. Available from: https://doi.org/10.1007/s00464-018-6407-6

162. Goldenberg MG, Goldenberg L, Grantcharov TP. Surgeon Performance Predicts Early Continence After Robot-Assisted Radical Prostatectomy. J Endourol. 2017;31(9):858–63.

163. Hung AJ, Jayaratna IS, Teruya K, Desai MM, Gill IS, Goh AC. Comparative assessment of three standardized robotic surgery training methods. BJU Int. 2013 Oct;112(6):864–71.

164. Ramos P, Montez J, Tripp A, Ng CK, Gill IS, Hung AJ. Face, content, construct and concurrent validity of dry laboratory exercises for robotic training using a global assessment tool. BJU Int. 2014;113(5):836–42.

165. Dubin AK, Smith R, Julian D, Tanaka A, Mattingly P. A Comparison of Robotic Simulation Performance on Basic Virtual Reality Skills: Simulator Subjective Versus Objective Assessment Tools. J Minim Invasive Gynecol. 2017 Nov 1;24(7):1184–9.

166. Almarzouq A, Hu J, Noureldin YA, Yin A, Anidjar M, Bladou F, et al. Are basic robotic surgical skills transferable from the simulator to the operating room? A randomized, prospective, educational study. Canadian Urological Association Journal. 2020 Jun 1;14(12).

167. Ross T, Raison N, Wallace L, Wood T, Lovegrove C, Van der Poel H, et al. Robot-assisted training - Expert performance in full immersion simulation, setting the benchmark (concurrent validity). Journal of Urology. 2017 Apr;197(4S).

168. Valdis M, Chu MWA, Schlachta CM, Kiaii B. Validation of a Novel Virtual Reality Training Curriculum for Robotic Cardiac Surgery A Randomized Trial. 2015.

169. Valdis M, Chu MWA, Schlachta C, Kiaii B. Evaluation of robotic cardiac surgery simulation training: A randomized controlled trial. Journal of Thoracic and Cardiovascular Surgery. 2016 Jun 1;151(6):1498-1505.e2.

170. Monda SM, Weese JR, Anderson BG, Vetter JM, Venkatesh R, Du K, et al. Development and Validity of a Silicone Renal Tumor Model for Robotic Partial Nephrectomy Training. Urology. 2018 Apr 1;114:114–20.

171. Timberlake MD, Garbens A, Schlomer BJ, Kavoussi NL, Kern AJM, Peters CA, et al. Design and validation of a low-cost, high-fidelity model for robotic pyeloplasty simulation training. J Pediatr Urol. 2020 Jun 1;16(3):332–9.

172. Butterworth J, Sadry M, Julian D, Haig F. Assessment of the training program for Versius, a new innovative robotic system for use in minimal access surgery. BMJ Surg Interv Health Technologies [Internet]. 2021;3:57. Available from: http://sit.bmj.com/

173. Raison N, Harrison · Patrick, Abe T, Aydin A, Ahmed K, Dasgupta · Prokar. Procedural virtual reality simulation training for robotic surgery: a randomised controlled trial. Surg Endosc [Internet]. 2021;35:6897–902. Available from: https://doi.org/10.1007/s00464-020-08197-w

174. Tarr ME, Anderson-Montoya BL, Vilasagar S, Myers EM. Validation of a Simulation Model for Robotic Sacrocolpopexy. Female Pelvic Med Reconstr Surg. 2022 Jan 1;28(1):14–9.

175. Sarcona J, Mikhail D, Tabibzadeh A, Nassau D, Kozel Z, Vira M, et al. Correlating Crowd-Sourced Assessment of Technical Skills (CSATS) with Post-Operative Complication Rates in Urological Surgery. J Urol. 2020;

176. Liang MI, McCann GA, Rath KS, Backes FJ, Cansino C, Salani R. Training the next generation of robotic surgeons using guided mentorship: A randomized controlled trial. J Minim Invasive Gynecol. 2014;21(6):1075–9.

177. Carter SC, Chiang A, Shah G, Kwan L, Montgomery JS, Karam A, et al. Video-based Peer Feedback Through Social Networking for Robotic Surgery Simulation: A Multicenter Randomized Controlled Trial. Ann Surg. 2015 May 1;261(5):870–5.

178. Bendre HH, Rajender A, Philip ·, Barbosa V, Wason SEL. Robotic dismembered pyeloplasty surgical simulation using a 3D-printed silicone-based model: development, face validation and crowdsourced learning outcomes assessment. J Robot Surg [Internet]. 2020;14:897–902. Available from: https://doi.org/10.1007/s11701-020-01072-9

179. Chen C, White L, Kowalewski T, Aggarwal R, Lintott C, Comstock B, et al. Crowd-sourced assessment of technical skills: A novel method to evaluate surgical performance. Journal of Surgical Research. 2014 Mar;187(1):65–71.

180. Goldenberg MG, Nabhani J, Wallis CJD, Chopra S, Hung AJ, Schuckman A, et al. Feasibility of expert and crowd-sourced review of intraoperative video for quality improvement of intracorporeal urinary diversion during robotic radical cystectomy. Canadian Urological Association Journal. 2017 Oct 1;11(10):331–6.

181. Mills JT, Hougen HY, Bitner D, Krupski TL, Schenkman NS. Does Robotic Surgical Simulator Performance Correlate With Surgical Skill? J Surg Educ. 2017 Nov 1;74(6):1052–6.

182. Addison P, Yoo A, Duarte-Ramos J, Addy J, Dechario S, Husk G, et al. Correlation between operative time and crowd-sourced skills assessment for robotic bariatric surgery. Surg Endosc. 2021 Sep 1;35(9):5303–9.

183. Tarr ME, Rivard C, Petzel AE, Summers S, Mueller ER, Rickey LM, et al. Robotic objective structured assessment of technical skills: A randomized multicenter dry laboratory training pilot study. Female Pelvic Med Reconstr Surg. 2014;20(4):228–36.

184. Knab LM, Zureikat AH, Zeh HJ, Hogg ME. Towards standardized robotic surgery in gastrointestinal oncology. Vol. 402, Langenbeck’s Archives of Surgery. Springer Verlag; 2017. p. 1003–14.

185. Tam V, Zenati M, Novak S, Chen Y, Zureikat AH, Zeh HJ, et al. Robotic Pancreatoduodenectomy Biotissue Curriculum has Validity and Improves Technical Performance for Surgical Oncology Fellows. J Surg Educ. 2017 Nov;74(6):1057–65.

186. Rice MJK, Zenati MS, Novak SM, Al Abbas AI, Zureikat AH, Zeh HJ, et al. Crowdsourced Assessment of Inanimate Biotissue Drills: A Valid and Cost-Effective Way to Evaluate Surgical Trainees. J Surg Educ. 2019 May 1;76(3):814–23.

187. Curry M, Malpani A, Li R, Tantillo T, Jog A, Blanco R, et al. Objective assessment in residency-based training for transoral robotic surgery. Laryngoscope. 2012;122(10):2184–92.

188. Alemozaffar M, Narayanan R, Percy AA, Minnillo BB, Steinberg P, Haleblian G, et al. Validation of a novel, tissue-based simulator for robot-assisted radical prostatectomy. J Endourol. 2014 Aug 1;28(8):995–1000.

189. Hernandez JD, Bann SD, Munz Y, Moorthy K, Datta V, Martin S, et al. Qualitative and quantitative analysis of the learning curve of a simulated surgical task on the da Vinci system. Surgical Endoscopy and Other Interventional Techniques. 2004 Mar;18(3):372–8.

190. Hutchinson K, Li Z, Cantrell LA, Schenkman NS, Alemzadeh H. Analysis of Executional and Procedural Errors in Dry‐lab Robotic Surgery Experiments. The International Journal of Medical Robotics and Computer Assisted Surgery [Internet]. 2022 Feb 3; Available from: https://onlinelibrary.wiley.com/doi/10.1002/rcs.2375

191. Vogell A, Wright V, Wright K. An Evaluation of the Utility of Robotic Virtual Reality Simulation in Gynecologic Resident Surgical Education. Journal of minimally invasive gynaecology. 2014;21(6 supplement).

192. Chen QY, Zhong Q, Liu ZY, Li P, Wang JB, Lin JX, et al. Surgical Outcomes, Technical Performance and Surgery Burden of Robotic Total Gastrectomy for Locally Advanced Gastric Cancer: A Prospective Study. Ann Surg [Internet]. 2021; Available from: http://links.lww.com/SLA/C930

193. Korets R, Mues AC, Graversen JA, Gupta M, Benson MC, Cooper KL, et al. Validating the use of the Mimic dV-trainer for robotic surgery skill acquisition among urology residents. Urology. 2011 Dec;78(6):1326–30.

194. Ahmad SB, Rice M, Chang C, Hamad A, Kingham TP, He J, et al. Will It Play in Peoria? A Pilot Study of a Robotic Skills Curriculum for Surgical Oncology Fellows. Ann Surg Oncol [Internet]. 2021;28:6273–82. Available from: https://doi.org/10.1245/s10434-021-09913-z

195. Zwart MJW, Jones LR, Fuente I, Balduzzi A, Takagi K, Novak S, et al. Performance with robotic surgery versus 3D- and 2D­laparoscopy during pancreatic and biliary anastomoses in a biotissue model: pooled analysis of two randomized trials. Surg Endosc. 2022 Jun 1;36(6):4518–28.

196. Moncayo S, Compagnon R, Caire F, Grosos C, Bahans C, Ilhero P, et al. Transition effects from laparoscopic to robotic surgery skills in small cavities. J Robot Surg. 2020 Jun 1;14(3):525–30.

197. Lee JY, Mattar T, Parisi TJ, Carlsen BT, Bishop AT, Shin AY. Learning curve of robotic-assisted microvascular anastomosis in the rat. J Reconstr Microsurg. 2012;28(7):451–6.

198. Vedula SS, Malpani A, Ahmidi N, Khudanpur S, Hager G, Chen CCG. Task-Level vs. Segment-Level Quantitative Metrics for Surgical Skill Assessment. J Surg Educ. 2016 May;73(3):482–9.

199. Hung AJ, Ng CK, Patil MB, Zehnder P, Huang E, Aron M, et al. Validation of a novel robotic-assisted partial nephrectomy surgical training model. BJU Int. 2012 Sep;110(6):870–4.

200. Hung AJ, Patil MB, Zehnder P, Cai J, Ng CK, Aron M, et al. Concurrent and predictive validation of a novel robotic surgery simulator: A prospective, randomized study. Journal of Urology. 2012;187(2):630–7.

201. Culligan P, Gurshumov E, Lewis C, Priestley J, Komar J, Salamon C. Predictive validity of a training protocol using a robotic surgery simulator. Female Pelvic Med Reconstr Surg. 2014;20(1):48–51.

202. Siddiqui NY, Galloway ML, Geller EJ, Green IC, Hur HC, Langston K, et al. Validity and reliability of the robotic objective structured assessment of technical skills. Obstetrics and Gynecology. 2014;123(6):1193–9.

203. Polin MR, Siddiqui NY, Comstock BA, Hesham H, Brown C, Lendvay TS, et al. Crowdsourcing: a valid alternative to expert evaluation of robotic surgery skills. In: American Journal of Obstetrics and Gynecology. Mosby Inc.; 2016. p. 644.e1-644.e7.

204. Newcomb LK, Bradley MS, Truong T, Tang M, Comstock B, Li YJ, et al. Correlation of Virtual Reality Simulation and Dry Lab Robotic Technical Skills. J Minim Invasive Gynecol. 2018 May;25(4):689–96.

205. Liu M, Purohit S, Mazanetz J, Allen W, Kreaden US, Curet M. Assessment of Robotic Console Skills (ARCS): construct validity of a novel global rating scale for technical skills in robotically assisted surgery. Surg Endosc. 2018 Jan 1;32(1):526–35.

206. Gerull W, Zihni A, Awad M. Operative performance outcomes of a simulator-based robotic surgical skills curriculum. Surg Endosc [Internet]. 2020;34:4543–8. Available from: https://doi.org/10.1007/s00464-019-07243-6

207. Selber JC, Alrasheed T. Robotic microsurgical training and evaluation. Semin Plast Surg. 2014 Feb;28(1):5–10.

208. Haque TF, Hui A, You J, Ma R, Nguyen JH, Lei X, et al. An Assessment Tool to Provide Targeted Feedback to Robotic Surgical Trainees: Development and Validation of the End-to-End Assessment of Suturing Expertise (EASE). Urol Pract. 2022 Nov;9(6):532–9.

209. Hutchins AR, Manson RJ, Lerebours R, Farjat AE, Cox ML, Mann BP, et al. Objective Assessment of the Early Stages of the Learning Curve for the Senhance Surgical Robotic System. J Surg Educ. 2018 Jan 1;76(1):201–14.

210. Arain NA, Dulan G, Hogg DC, Rege R V., Powers CE, Tesfay ST, et al. Comprehensive proficiency-Based inanimate training for robotic surgery: Reliability, feasibility, and educational benefit. Surg Endosc. 2012;26(10):2740–5.

211. Dulan G, Rege R V., Hogg DC, Gilberg-Fisher KM, Arain NA, Tesfay ST, et al. Proficiency-based training for robotic surgery: Construct validity, workload, and expert levels for nine inanimate exercises. Surg Endosc. 2012;26(6):1516–21.

212. Dulan G, Rege R V., Hogg DC, Gilberg-Fisher KM, Arain NA, Tesfay ST, et al. Developing a comprehensive, proficiency-based training program for robotic surgery. Surgery (United States). 2012 Sep;152(3):477–88.

213. Bric J, Connolly M, Kastenmeier A, Goldblatt M, Gould JC. Proficiency training on a virtual reality robotic surgical skills curriculum. Surg Endosc. 2014 Dec 1;28(12):3343–8.

214. Suh IH, Lagrange CA, Oleynikov D, Siu KC. Evaluating robotic surgical skills performance under distractive environment using objective and subjective measures. Surg Innov. 2016 Feb 1;23(1):78–89.

215. Hung AJ, Zehnder P, Patil MB, Cai J, Ng CK, Aron M, et al. Face, content and construct validity of a novel robotic surgery simulator. Journal of Urology. 2011;186(3):1019–25.

216. Kelly DC, Margules AC, Kundavaram CR, Narins H, Gomella LG, Trabulsi EJ, et al. Face, content, and construct validation of the da Vinci Skills Simulator. Urology. 2012 May;79(5):1068–72.

217. Alzahrani T, Haddad R, Alkhayal A, Delisle J, Drudi L, Gotlieb W, et al. Validation of the da vinci surgical skill simulator across three surgical disciplines:A pilot study. Journal of the Canadian Urological Association. 2013;7(7–8).

218. Lyons C, Goldfarb D, Jones SL, Badhiwala N, Miles B, Link R, et al. Which skills really matter? proving face, content, and construct validity for a commercial robotic simulator. Surg Endosc. 2013;27(6):2020–30.

219. Foell K, Finello A, Yasufuku K, Benardini M, Waddell T, Pace K, et al. Robotic surgery basic skills training: Evaluation of a pilot multidisciplinary simulation-based curriculum. Canadian Urological Association Journal. 2013;

220. Cecilie Havemann M, Dalsgaard T, Led Sørensen J, Røssaak K, Brisling S, Jul Mosgaard B, et al. Examining validity evidence for a simulation-based assessment tool for basic robotic surgical skills. J Robot Surg [Internet]. 2019;13:99–106. Available from: https://doi.org/10.1007/s11701-018-0811-8

221. Perrenot C, Perez M, Tran N, Jehl JP, Felblinger J, Bresler L, et al. The virtual reality simulator dV-Trainer is a valid assessment tool for robotic surgical skills. Surg Endosc. 2012;2587–93.

222. Liss MA, Abdelshehid C, Quach S, Lusch A, Graversen J, Landman J, et al. Validation, correlation, and comparison of the da Vinci Trainer ^TM^ and the da Vinci surgical skills simulator^TM^ using the Mimic^TM^ software for urologic robotic surgical education. J Endourol. 2012 Dec 1;26(12):1629–34.

223. Hertz AM, George EI, Vaccaro CM, Brand TC. Head-to-Head Comparison of Three Virtual-Reality Robotic Surgery Simulators. Journal of the Society of Laparoendoscopic Surgeons. 2018 Jan 1;22(1).

224. Sheth SS, Fader AN, Tergas AI, Kushnir CL, Green IC. Virtual reality robotic surgical simulation: An analysis of gynecology trainees. J Surg Educ. 2014 Jan;71(1):125–32.

225. Connolly M, Seligman J, Kastenmeier A, Goldblatt M, Gould JC. Validation of a virtual reality-based robotic surgical skills curriculum. Surg Endosc. 2014;28(5):1691–4.

226. Brown K, Mosley N, Tierney J. Battle of the bots: a comparison of the standard da Vinci and the da Vinci Surgical Skills Simulator in surgical skills acquisition. J Robot Surg. 2017;11.

227. Finnegan KT, Meraney AM, Staff I, Shichman SJ. Da vinci skills simulator construct validation study: Correlation of prior robotic experience with overall score and time score simulator performance. Urology. 2012;80(2):330–6.

228. Liss MA, Kane CJ, Chen T, Baumgartner J, Derweesh IH. Virtual reality suturing task as an objective test for robotic experience assessment. BMC Urol. 2015 Jul 3;15(1).

229. Yamany T, Woldu SL, Korets R, Badani KK. Effect of postcall fatigue on surgical skills measured by a robotic simulator. J Endourol. 2015 Apr 1;29(4):479–84.

230. Vogell A, Gurjal H, Wright K, Ruthazer R. Impact of a robotic simulation program on resident surgical performance. American Journal of Obstetrics and Gynaecology [Internet]. 2015; Available from: http://dx.doi.org/10.1016/j.ajog.

231. Brinkman WM, Luursema JM, Kengen B, Schout BMA, Witjes JA, Bekkers RL. Da vinci skills simulator for assessing learning curve and criterion-based training of robotic basic skills. Urology. 2013 Mar;81(3):562–6.

232. Robison W, Patel SK, Mehta A, Senkowski T, Allen J, Shaw E, et al. Can fatigue affect acquisition of new surgical skills? A prospective trial of pre- and post-call general surgery residents using the da Vinci surgical skills simulator. Surg Endosc. 2018 Mar 1;32(3):1389–96.

233. Gleason A, Servais E, Quadri S, Manganiello M, Cheah YL, Simon CJ, et al. Developing basic robotic skills using virtual reality simulation and automated assessment tools: a multidisciplinary robotic virtual reality-based curriculum using the Da Vinci Skills Simulator and tracking progress with the Intuitive Learning platform. J Robot Surg. 2022;

234. Kenney PA, Wszolek MF, Gould JJ, Libertino JA, Moinzadeh A. Face, Content, and Construct Validity of dV-Trainer, a Novel Virtual Reality Simulator for Robotic Surgery. Urology. 2009 Jun;73(6):1288–92.

235. Kang SG, Cho S, Kang SH, Haidar AM, Samavedi S, Palmer KJ, et al. The tube 3 module designed for practicing vesicourethral anastomosis in a virtual reality robotic simulator: Determination of face, content, and construct validity. Urology. 2014;84(2):345–50.

236. Schreuder HWR, Persson JEU, Wolswijk RGH, Ihse I, Schijven MP, Verheijen RHM. Validation of a novel virtual reality simulator for robotic surgery. The Scientific World Journal. 2014;2014.

237. Cho JS, Hahn KY, Kwak JM, Kim J, Baek SJ, Shin JW, et al. Virtual reality training improves da vinci performance: A prospective trial. Vol. 23, Journal of Laparoendoscopic and Advanced Surgical Techniques. Mary Ann Liebert Inc.; 2013. p. 992–8.

238. Lendvay TS, Brand TC, White L, Kowalewski T, Jonnadula S, Mercer LD, et al. Virtual reality robotic surgery warm-up improves task performance in a dry laboratory environment: A prospective randomized controlled study. J Am Coll Surg. 2013 Jun;216(6):1181–92.

239. Ruparel RK, Taylor AS, Patel J, Patel VR, Heckman MG, Rawal B, et al. Assessment of virtual reality robotic simulation performance by urology resident trainees. J Surg Educ. 2014;71(3):302–8.

240. Sethi AS, Peine WJ, Mohammadi Y, Sundaram CP. Validation of a Novel Virtual Reality Robotic Simulator. J Endourol [Internet]. 2009;23(3):503–8. Available from: www.liebertpub.com

241. Kim JY, Kim S Bin, Pyun JH, Kim HK, Cho S, Lee JG, et al. Concurrent and predictive validation of robotic simulator tube 3 module. Korean J Urol. 2015 Nov 1;56(11):756–61.

242. Schommer E, Patel VR, Mouraviev V, Thomas C, Thiel DD. Diffusion of Robotic Technology Into Urologic Practice has Led to Improved Resident Physician Robotic Skills. J Surg Educ. 2017 Jan 1;74(1):55–60.

243. Watkinson W, Raison N, Abe T, Harrison P, Khan S, Van der Poel H, et al. Establishing objective benchmarks in robotic virtual reality simulation at the level of a competent surgeon using the RobotiX Mentor simulator. Postgrad Med J. 2018 May 1;94(1111):270–7.

244. Leijte E, De Blaauw I, Rosman C, Botden SMBI. Assessment of validity evidence for the RobotiX robot assisted surgery simulator on advanced suturing tasks. BMC Surg [Internet]. 2020 [cited 2022 Jun 21]; Available from: https://doi.org/10.1186/s12893-020-00839-z

245. Whittaker G, Aydin A, Raison N, Kum F, Challacombe B, Khan MS, et al. Validation of the RobotiX Mentor Robotic Surgery Simulator. J Endourol. 2016 Mar 1;30(3):338–46.

246. Hovgaard LH, Arild S, Andersen W, Konge L, Dalsgaard T, Christian ·, et al. Validity evidence for procedural competency in virtual reality robotic simulation, establishing a credible pass/fail standard for the vaginal cuff closure procedure. Surg Endosc [Internet]. 2018;32:4200–8. Available from: https://doi.org/10.1007/s00464-018-6165-5

247. Alshuaibi M, Perrenot C, Hubert J, Perez M. Concurrent, face, content, and construct validity of the RobotiX Mentor simulator for robotic basic skills. International Journal of Medical Robotics and Computer Assisted Surgery. 2020 Jun 1;16(3).

248. McDonough PS, Tausch TJ, Peterson AC, Brand TC. Initial validation of the ProMIS surgical simulator as an objective measure of robotic task performance. J Robot Surg. 2011 Sep;5(3):195–9.

249. Jonsson M, Mahmood M, Askerud T, Hellborg H, Ramel S, Wiklund P, et al. PromisTM Can Serve as a da Vinci Simulator - A Construct Validity Study. J Endourol. 2011;25(2):345–50.

250. Chandra V, Nehra D, Parent R, Woo R, Reyes R, Hernandez-Boussard T, et al. A comparison of laparoscopic and robotic assisted suturing performance by experts and novices. Surgery. 2010;147:830–9.

251. Seixas-Mikelus SA, Kesavadas T, Srimathveeravalli G, Chandrasekhar R, Wilding GE, Guru KA. Face Validation of a Novel Robotic Surgical Simulator. Urology. 2010 Aug 1;76(2):357–60.

252. Seixas-Mikelus SA, Stegemann AP, Kesavadas T, Srimathveeravalli G, Sathyaseelan G, Chandrasekhar R, et al. Content validation of a novel robotic surgical simulator. BJU Int. 2011 Apr;107(7):1130–5.

253. Chowriappa AJ, Shi Y, Raza SJ, Ahmed K, Stegemann A, Wilding G, et al. Development and validation of a composite scoring system for robot-assisted surgical training - the Robotic Skills Assessment Score. Journal of Surgical Research. 2013;185(2):561–9.

254. Cowan A, Chen J, Mingo S, Reddy SS, Ma R, Marshall S, et al. Virtual Reality vs Dry Laboratory Models: Comparing Automated Performance Metrics and Cognitive Workload during Robotic Simulation Training. J Endourol. 2021 Oct 1;35(10):1571–6.

255. Nguyen JH, Chen J, Marshall SP, Ghodoussipour S, Chen A, Gill IS, et al. Using objective robotic automated performance metrics and task-evoked pupillary response to distinguish surgeon expertise. World J Urol [Internet]. 2020 [cited 2022 Feb 23]; Available from: https://www.springer.com/aam-

256. Verner L, Oleynikov D, Holtmann S, Haider H, Zhukov L. Measurements of the level of surgical expertise using flight path analysis from da Vinci Robotic Surgical System. Stud Health Technol Inform [Internet]. 2003; Available from: https://www.researchgate.net/publication/8259434

257. Narazaki K, Oleynikov D, Stergiou N. Objective assessment of proficiency with bimanual inanimate tasks in robotic laparoscopy. Journal of Laparoendoscopic and Advanced Surgical Techniques. 2007 Feb;17(1):47–52.

258. Judkins TN, Oleynikov D, Stergiou N. Objective evaluation of expert and novice performance during robotic surgical training tasks. Surgical Endoscopy and Other Interventional Techniques. 2009 Mar;23(3):590–7.

259. Hung AJ, Oh PJ, Chen J, Ghodoussipour S, Lane C, Jarc A, et al. Experts vs super-experts: differences in automated performance metrics and clinical outcomes for robot-assisted radical prostatectomy. BJU Int. 2019 May 1;123(5):861–8.

260. Narazaki K, Oleynikov D, Stergiou N. Robotic surgery training and performance Identifying objective variables for quantifying the extent of proficiency. Surg Endosc. 2006;

261. Lefor AK, Harada K, Dosis A, Mitsuishi M. Motion analysis of the JHU–ISI Gesture and Skill Assessment Working Set II: learning curve analysis. Int J Comput Assist Radiol Surg. 2021 Apr 1;16(4):589–95.

262. Tausch TJ, Kowalewski TM, White LW, McDonough PS, Brand TC, Lendvay TS. Content and construct validation of a robotic surgery curriculum using an electromagnetic instrument tracker. Journal of Urology. 2012 Sep;188(3):919–23.

263. Walker JL, Nathwani JN, Mohamadipanah H, Laufer S, Jocewicz FF, Gwillim E, et al. Residents’ response to bleeding during a simulated robotic surgery task. Journal of Surgical Research. 2017 Dec 1;220:385–90.

264. Nisky I, Okamura AM, Hsieh MH. Effects of robotic manipulators on movements of novices and surgeons. Surg Endosc. 2014;28(7):2145–58.

265. Nisky I, Hsieh MH, Okamura AM. The effect of a robot-assisted surgical system on the kinematics of user movements. In: Proceedings of the Annual International Conference of the IEEE Engineering in Medicine and Biology Society, EMBS. 2013. p. 6257–60.

266. Jog A, Itkowitz B, Liu M, DiMaio S, Hager G, Curet M, et al. Towards integrating task information in skills assessment for dexterous tasks in surgery and simulation. In: Proceedings - IEEE International Conference on Robotics and Automation. 2011. p. 5273–8.

267. Kumar R, Jog A, Malpani A, Vagvolgyi B, Yuh D, Nguyen H, et al. Assessing system operation skills in robotic surgery trainees. International Journal of Medical Robotics and Computer Assisted Surgery. 2011;

268. Tao L, Elhamifar E, Khudanpur S, Hager GD, Vidal R. Sparse hidden Markov models for surgical gesture classification and skill evaluation. In: Lecture Notes in Computer Science (including subseries Lecture Notes in Artificial Intelligence and Lecture Notes in Bioinformatics). 2012.

269. Fard MJ, Ameri S, Darin Ellis R, Chinnam RB, Pandya AK, Klein MD. Automated robot-assisted surgical skill evaluation: Predictive analytics approach. International Journal of Medical Robotics and Computer Assisted Surgery. 2018 Feb 1;14(1).

270. Pan JH, Gao J, Zheng WS. Action assessment by joint relation graphs. In: Proceedings of the IEEE International Conference on Computer Vision. 2019.

271. Funke I, Mees ST, Weitz J, Speidel S. Video-based surgical skill assessment using 3D convolutional neural networks. Int J Comput Assist Radiol Surg. 2019;14(7).

272. Ming Y, Cheng Y, Chunchen W, Meng L, Guang Z, Feng C. Automated Objective Basic Surgical Skills Assessment: Overall Kinematic Performance Assessment Method. In: IEEE Xplore. Institute of Electrical and Electronics Engineers Inc.; 2020. p. 74–8.

273. Tang Y, Ni Z, Zhou J, Zhang D, Lu J, Wu Y, et al. Uncertainty-Aware Score Distribution Learning for Action Quality Assessment. In: Proceedings of the IEEE Computer Society Conference on Computer Vision and Pattern Recognition. 2020.

274. Lyman WB, Passeri MJ, Murphy · Keith, Siddiqui IA, Adeel ·, Khan S, et al. An objective approach to evaluate novice robotic surgeons using a combination of kinematics and stepwise cumulative sum (CUSUM) analyses. Surg Endosc [Internet]. 2021;35:2765–72. Available from: https://doi.org/10.1007/s00464-020-07708-z

275. Yu X, Rao Y, Zhao W, Lu J, Zhou J. Group-aware Contrastive Regression for Action Quality Assessment. In: Proceedings of the IEEE International Conference on Computer Vision. 2021.

276. Zhang J, Nie Y, Lyu Y, Yang X, Chang J, Zhang JJ. SD-Net: joint surgical gesture recognition and skill assessment. Int J Comput Assist Radiol Surg. 2021;16(10).

277. Soleymani A, Li X, Tavakoli M. A Domain-Adapted Machine Learning Approach for Visual Evaluation and Interpretation of Robot-Assisted Surgery Skills. IEEE Robot Autom Lett. 2022 Jul;7(3):8202–8.

278. Juarez-Villalobos L, Hevia-Montiel N, Perez-Gonzalez J. Machine Learning based Classification of Local Robotic Surgical Skills in a Training Tasks Set. In: Proceedings of the Annual International Conference of the IEEE Engineering in Medicine and Biology Society, EMBS. Institute of Electrical and Electronics Engineers Inc.; 2021. p. 4596–9.

279. Kumar R, Jog A, Vagvolgyi B, Nguyen H, Hager G, Chen CCG, et al. Objective measures for longitudinal assessment of robotic surgery training. Journal of Thoracic and Cardiovascular Surgery. 2012 Mar;143(3):528–34.

280. Ahmidi N, Gao Y, Béjar B, Vedula SS, Khudanpur S, Vidal R, et al. String motif-based description of tool motion for detecting skill and gestures in robotic surgery. In: Lecture Notes in Computer Science (including subseries Lecture Notes in Artificial Intelligence and Lecture Notes in Bioinformatics). 2013.

281. Malpani A, Swaroop Vedula · S, Chiung C, Chen G, Hager GD. A study of crowdsourced segment-level surgical skill assessment using pairwise rankings. Int J CARS. 2015;10:1435–47.

282. Lajkó G, Elek RN, Haidegger T. Endoscopic Image-Based Skill Assessment in Robot-Assisted Minimally Invasive Surgery. Sensors [Internet]. 2021;21:5412. Available from: https://doi.org/10.3390/s21165412.https://doi.org/10.3390/s21165412https://www.mdpi.com/journal/sensors

283. Takács K, Haidegger T. Adaptive Neuro-fuzzy Inference System for Automated Skill Assessment in Robot-Assisted Minimally Invasive Surgery. In: INES 2021 - IEEE 25th International Conference on Intelligent Engineering Systems, Proceedings. 2021.

284. Brown KC, Bhattacharyya KD, Kulason S, Zia A, Jarc A. How to bring surgery to the next level: Interpretable skills assessment in robotic-assisted surgery. Visc Med. 2020 Dec 1;36(6):463–70.

285. Goldenberg MG, Grantcharov TP. A Novel Method of Setting Performance Standards in Surgery Using Patient Outcomes. Ann Surg. 2019 Jan 1;269(1):79–82.

286. Abdelaal AE, Avinash A, Kalia M, Hager GD, Salcudean SE. A multi-camera, multi-view system for training and skill assessment for robot-assisted surgery. Int J Comput Assist Radiol Surg. 2020 Aug 1;15(8):1369–77.

287. Altok M, Achim MF, Matin SF, Pettaway CA, Chapin BF, Davis JW. A decade of robot-assisted radical prostatectomy training: Time-based metrics and qualitative grading for fellows and residents. Urologic Oncology: Seminars and Original Investigations. 2018 Jan 1;36(1):13.e19-13.e25.

288. Harris DJ, Vine SJ, Wilson MR, McGrath JS, LeBel ME, Buckingham G. The effect of observing novice and expert performance on acquisition of surgical skills on a robotic platform. PLoS One. 2017 Nov 1;12(11).

289. Elhage O, Challacombe B, Shortland A, Dasgupta P. An assessment of the physical impact of complex surgical tasks on surgeon errors and discomfort: A comparison between robot-assisted, laparoscopic and open approaches. BJU Int. 2015 Feb 1;115(2):274–81.

290. Guru KA, Esfahani ET, Raza SJ, Bhat R, Wang K, Hammond Y, et al. Cognitive skills assessment during robot-assisted surgery: Separating the wheat from the chaff. BJU Int. 2015 Jan 1;115(1):166–74.

291. Lerner MA, Ayalew M, Peine WJ, Sundaram CP. Does Training on a Virtual Reality Robotic Simulator Improve Performance on the da Vinci Ò Surgical System? J Endourol [Internet]. 2010; Available from: www.liebertpub.com

292. Gavazzi A, Bahsoun A, Van Haute W, Ahmed K, Elhage O, Jaye P, et al. Face, content and construct validity of a virtual reality simulator for robotic surgery (SEP Robot). The Annals of The Royal College of Surgeons of England. 2011;93:152–6.

293. Simmonds C, Brentnall M, Lenihan J. Evaluation of a novel universal robotic surgery virtual reality simulation proficiency index that will allow comparisons of users across any virtual reality simulation curriculum. Surg Endosc. 2021 Oct 1;35(10):5867–75.

294. Jiang J, Xing Y, Wang S, Liang K. Evaluation of robotic surgery skills using dynamic time warping. Comput Methods Programs Biomed. 2017 Dec 1;152:71–83.

295. Busch C, Nakadate R, Uemura M, Obata S, Jimbo T, Hashizume M. Objective assessment of robotic suturing skills with a new computerized system: A step forward in the training of robotic surgeons. Asian J Endosc Surg. 2019 Oct 1;12(4):388–95.

296. Beulens AJ, Namba HF, Brinkman WM, Meijer RP, Koldewijn EL, M Hendrikx AJ, et al. Analysis of the video motion tracking system ‘Kinovea’ to assess surgical movements during robot-assisted radical prostatectomy. The International Journal of Medical Robotics and Computer Assisted Surgery [Internet]. 2020; Available from: https://doi.org/10.1002/rcs.2090

297. Zhang Y, Law H, Tae-Kyung K, Miller D, Montie J, Deng J. Surgeon technical skill assessment using computer vision-based analysis. Journal of Urology. 2018;199(4 Supplement 1).
